# Rumen microbiome structure and metabolites activity in dairy cows with clinical and subclinical mastitis

**DOI:** 10.1186/s40104-020-00543-1

**Published:** 2021-02-08

**Authors:** Yue Wang, Xuemei Nan, Yiguang Zhao, Linshu Jiang, Mengling Wang, Hui Wang, Fan Zhang, Fuguang Xue, Dengke Hua, Jun Liu, Junhu Yao, Benhai Xiong

**Affiliations:** 1 State Key Laboratory of Animal Nutrition, Institute of Animal Science, Chinese Academy of Agricultural Sciences, Beijing, 100193 China; 2 College of Animal Science and Technology, Northwest A&F University, Yangling, 712100 China; 3 Beijing Key Laboratory for Dairy Cow Nutrition, Beijing University of Agriculture, Beijing, 102206 China; 4 Engineering Research Center of Feed Development, Jiangxi Province Key Laboratory of Animal Nutrition, Jiangxi Agricultural University, Nanchang, 330045 China; 5 Langfang Academy of Agriculture and Forestry, Langfang, 065000 China

**Keywords:** Dairy cows, Lactation performance, Mastitis, Rumen fermentation, Ruminal metabolisms, Ruminal microbiota

## Abstract

**Background:**

Due to the high prevalence and complex etiology, bovine mastitis (BM) is one of the most important diseases to compromise dairy cow health and milk quality. The shift in milk compositions has been widely investigated during mastitis, but recent studies suggested that gastrointestinal microorganism also has a crucial effect on the inflammation of other peripheral tissues and organs, including the mammary gland. However, research focused on the variation of rumen inner-environment during mastitis is still limited. Therefore, the ruminal microbial profiles, metabolites, and milk compositions in cows with different udder health conditions were compared in the present study. Furthermore, the correlations between udder health status and ruminal conditions were investigated. Based on the somatic cell counts (SCC), California mastitis test (CMT) parameters and clinical symptoms of mastitis, 60 lactating Holstein dairy cows with similar body conditions (excepted for the udder health condition) were randomly divided into 3 groups (*n* = 20 per group) including the healthy (H) group, the subclinical mastitis (SM) group and the clinical mastitis (CM) group. Lactation performance and rumen fermentation parameters were recorded. And rumen microbiota and metabolites were also analyzed via 16S rRNA amplicon sequencing and untargeted metabolomics, respectively.

**Results:**

As the degree of mastitis increased, rumen lactic acid (LA) (*P* < 0.01), acetate, propionate, butyrate, valerate (*P* < 0.001), and total volatile fatty acids (TVFAs) (*P* < 0.01) concentrations were significantly decreased. In the rumen of CM cows, the significantly increased bacteria related to intestinal and oral inflammation, such as Lachnospiraceae (FDR-adjusted *P* = 0.039), *Moraxella* (FDR-adjusted *P* = 0.011) and Neisseriaceae (FDR-adjusted *P* = 0.036), etc., were accompanied by a significant increase in 12-oxo-20-dihydroxy-leukotriene B4 (FDR-adjusted *P* = 5.97 × 10^− 9^) and 10beta-hydroxy-6beta-isobutyrylfuranoeremophilane (FDR-adjusted *P* = 3.88 × 10^− 10^). Meanwhile, in the rumen of SM cows, the *Ruminiclostridium_9* (FDR-adjusted *P* = 0.042) and *Enterorhabdus* (FDR-adjusted *P* = 0.043) were increased along with increasing methenamine (FDR-adjusted *P* = 6.95 × 10^− 6^), 5-hydroxymethyl-2-furancarboxaldehyde (5-HMF) (FDR-adjusted *P* = 2.02 × 10^− 6^) and 6-methoxymellein (FDR-adjusted *P* = 2.57 × 10^− 5^). The short-chain fatty acids (SCFAs)-producing bacteria and probiotics in rumen, including *Prevoterotoella_1* (FDR-adjusted *P* = 0.045) and *Bifidobacterium* (FDR-adjusted *P* = 0.035), etc., were significantly reduced, with decreasing 2-phenylbutyric acid (2-PBA) (FDR-adjusted *P* = 4.37 × 10^− 6^).

**Conclusion:**

The results indicated that there was a significant shift in the ruminal microflora and metabolites associated with inflammation and immune responses during CM. Moreover, in the rumen of cows affected by SM, the relative abundance of several opportunistic pathogens and the level of metabolites which could produce antibacterial compounds or had a competitive inhibitory effect were all increased.

**Supplementary Information:**

The online version contains supplementary material available at 10.1186/s40104-020-00543-1.

## Background

Bovine mastitis (BM) is commonly recognized as an intramammary infection (IMI) caused by pathogens. The main manifestations of clinical mastitis (CM) are increased body temperature, udder redness and pain, inflammatory cell infiltration, acinar duct edema, as well as interstitial bleeding, which result in elevated milk somatic cell counts (SCC) and decreased milk yield [1]. In contrast, the subclinical mastitis (SM) has high concealment and long incubation period. Although the udders of cows with SM do not have visible changes in the appearance, the increase of milk SCC does occur [1]. Usually, mastitis treated by antibiotics is easy to relapse, leading to a continuous decline in milk yield and quality. In addition, nipple necrosis accelerates the culling of cows [2]. Although SCC has been widely applied to diagnose the udder health, multitudinous factors can affect SCC [3]. Therefore, integrated approaches are used to improve the accuracy of IMI diagnosis, for instance, the combination of California mastitis test (CMT), milk pH value, conductivity, and enzyme test etc. [4].

Notably, recent studies have shown that gastrointestinal microbiota played a vital role in inflammation of tissues outside the gut, such as mammary gland [5–14]. First of all, dysbiosis in gastrointestinal microbiota may be one of the causes of BM. Intestinal bacteria may be derived from milk, and in turn affect the microorganism in the udder after the formation of intestinal flora [6]. Ma et al. [7] transplanted fecal bacteria from CM cows to sterile mice, and consequently led to mastitis in mice. Secondly, immune regulation and metabolism of endogenous substances are both potential related factors of mastitis, which also associated gastrointestinal microbiome [8]. Several studies in human have confirmed the direct or indirect relationships between gastrointestinal microorganisms and breast inflammation and immune regulation [9–11]. Besides, a considerable number of symbiotic bacteria distributed in the gastrointestinal tract are closely related to the immune regulation in dairy cows [12]. Probiotics can penetrate the intestinal epithelium, and reach the udder tissue through internal circulation [13], acting as an effective immune enhancer [7]. Thirdly, diet was also reported to play a vital role in determining the structure of microbiome and bioactivity of bacterial metabolites in the mammary gland. For example, Mediterranean diet can increase the abundance of *Lactobacillus*, bile acids and bacterial-modified metabolites and decrease reactive oxygen species and pro-inflammatory metabolites in the mammary gland of monkeys [14]. The previous studies have triggered the speculation on the relationship between the gastrointestinal environment and the health status of mammary gland, which should be verified by further investigation.

Unlike monogastric animals, ruminal microorganisms are the largest microbial group in the gastrointestinal tract. Ruminal bacteria fermentation in dairy cows produces large amounts of volatile fatty acids (VFAs), also called short-chain fatty acids (SCFAs), which are not only the energy source, but also the precursors of milk compositions [15]. Milk fat, lactose and protein are formed through acetate, butyrate, propionate and microbial proteins, which are produced by microbial fermentation of feed in the rumen [16]. Therefore, milk compositions are indirectly derived from the fermentation products of ruminal microorganisms. Therefore, rumen inner-environment has a decisive influence on milk quality. However, the correlation between the changes in rumen microorganisms and metabolites and the udder health of dairy cows is still in short of knowledge.

The present study hypothesized that the rumen inner-environment, including ruminal microorganism profile, fermentation parameters and metabolites activities, varied during IMI. Accordingly, we identified the ruminal microorganisms and metabolites in cows with SM, CM and healthy (H) udder by 16S rRNA sequencing and liquid chromatography-mass spectrometry (LC-MS), respectively. The current study probed into the possible shift in the profile of ruminal microflora and metabolites activities during mastitis, thereby improved the understanding of the relationship between rumen inner-environment and mastitis.

## Materials and methods

### Ethics statement

All experimental designs and protocols were approved by the Animal Ethics Committee of the Chinese Academy of Agricultural Sciences (Beijing, China; approval number: IAS-2019-6) and were in accordance with the recommendations of the academy’s guidelines for animal research.

### Animals, diets and experimental design

This study was carried out in a suburban dairy farm in Beijing, China. Total mixed rations (TMR) with a concentrate to forage ratio of 4:6 were offered to the Holstein cows three times a day at 07:00, 13:00, and 19:00, respectively. Cows were fed ad libitum and had free access to water. The TMR ingredients and nutritional compositions are shown in Additional file 1: Table S1. In the current study, the udder health status of cows was comprehensively judged according to the degree of inflammation in each quarter sample from each cow based on the clinical manifestations of the udder and the results of the CMT and SCC in milk [1]. CMT detection methods and judgment basis are listed in Additional file 1: Table S2. According to the results, 60 Holstein cows were selected and divided into 3 groups: 20 cows with healthy udder (H group) (SCC < 100,000 cells/mL; no clinical symptoms in udders; CMT results were negative); 20 cows with SM (SM group) (600,000 < SCC < 1,000,000 cells/mL; no obvious clinical symptoms of mastitis in the udder; CMT results were weakly positive); and 20 cows with CM (CM group) (SCC > 3000,000 cells/mL, with obvious signs of inflammation in udders, including udder swelling, redness and milk clots, etc., as well as positive or strongly positive CMT results). The basic information of cows including parity, days in milk, average daily milk yield, clinical symptoms in udders and milk SCC are displayed in Additional file 1: Table S3.

### Milk and rumen fluid sampling

Throughout the test period, the milk yield of selected cows was recorded for 3 consecutive days after confirmation of udder health status. Milking was performed 3 times a day using an automatic milking system (Ruishengyuan Machinery Assembly Co., Ltd., Hebei, China). Milk samples from H group were mixed from 4 quarters, while milk samples from cows with IMI (SM and CM groups) were only collected from inflammatory quarters. Milk samples from each cow were collected 3 times a day in the morning, noon and evening, respectively, with 50 mL each time, and mixed in a ratio of 4:3:3 [17]. Each milk sample was added 0.6 mg/mL potassium dichromate as a preservative and were then stored at 4 °C for analysis of milk compositions. Milk fat, protein, lactose and SCC were analyzed by a MilkoScan FT2 milk composition analyzer (FOSS, Copenhagen, Denmark) [17].

Before the morning feeding on the sampling day, a stomach tube (GCYQ-1-A, Guidi scientific instrument Co., Ltd., Shanghai, China) and a 200-mL syringe were used to collect rumen fluid samples from each cow. The first tube of rumen fluid was discarded to minimize saliva contamination and a 50-mL of rumen fluid was collected per cow. The pH value of the rumen fluid was immediately measured by a pH meter (8362sc Portable pH meter; Biyuntian biotechnology Co., Ltd., Shanghai, China) [18]. The remaining rumen fluid samples were poured into sterile test tubes and immediately stored in liquid nitrogen. These samples were then used to detect VFAs (GC-6890 N, Agilent, Palo Alto, USA), ammonia nitrogen (NH_3_-N) (HBS-1101 ELIASA, Huawei Delang Instrument Co., Ltd., Wuxi, China), ruminal fluid urea nitrogen (RUN) (GF-D200 semi-automatic biochemical analyzer; Gaomi Rainbow Biological Co., Ltd., Shandong, China), lactic acid (LA) (MultiskanAsc ELIASA, Thermo, USA) [15], microorganisms composition and metabolites.

### DNA extraction, PCR amplification and sequencing

Microbial community genomic DNA was extracted from rumen fluid samples using the FastDNA® Spin Kit for Soil (MP Biomedicals, California, USA) according to the manufacturer’s instructions. In brief, 1 mL rumen fluid, 978 μL sodium phosphate buffer and 122 μL methyltransferase (MT) buffer were added to a lysing matrix E tube, and mixed thoroughly. The tube was then centrifuged at 14,000 r/min for 10 min. Subsequently, the supernatant was transferred to a 1.5-mL centrifuge tube with a supplementation of 250 μL PPS. After centrifuging at 14,000 r/min at room temperature for 5 min, the supernatant was discarded. Then 500 μL 5.5 mol/L guanidine isothiocyanate solution was added and the mixture was transferred to the Spin Filter. After the addition of 500 μL salt/ethanol wash solution (SEWS-M), the Spin Filter was centrifuged at 14,000 r/min for 1 min and the filtrate was discarded. After a 3-min drying process, 100 μL DNA elution solution-ultra pure water (DES), which was preheated at 55 °C for 5 min, was added to the Spin Filter. Finally, total DNA was obtained after centrifuging at 14,000 r/min for 2 min [19]. The DNA extract was checked on 1% agarose gel, and DNA concentration and purity were determined by a NanoDrop 2000 UV-vis spectrophotometer (Thermo Scientific, Wilmington, USA). A total of 10 ng DNA were then used in the following sequencing analysis. The hypervariable V3-V4 region of the bacterial 16S rRNA gene was amplified with primer pairs 338F (5′-ACTCCTACGGGAGGCAGCAG-3′) and 806R (5′-GGACTACHVGGGTWTCTAAT-3′) by an ABI GeneAmp® 9700 PCR thermocycler (Thermo Fisher Scientific, Waltham, Mass, USA). PCR reactions were performed in triplicate. The PCR product was extracted from 2% agarose gel and purified using an AxyPrep DNA gel extraction kit (Axygen Biosciences, Union City, CA, USA) according to the manufacturer’s instructions and quantified using a Quantus™ Fluorometer (Promega, Beijing, China) [19]. The purified amplicons were pooled in equimolar and paired-end sequenced (2 × 300) on an Illumina MiSeq platform (Illumina, San Diego, USA) [20]. The raw sequences have been submitted to NCBI Sequence Read Archive (SRA) database (Accession Number: PRJNA669201).

### Processing, annotation, and statistical analysis of sequencing data

The raw 16S rRNA gene sequencing reads were de-multiplexed, quality-filtered by Trimmomatic and merged by FLASH. Operational taxonomic units (OTUs) with a 97% similarity cutoff were clustered using UPARSE (version 7.1, http://drive5.com/uparse/), and the chimeric sequences were identified and removed [21]. The taxonomy of each OTU representative sequence was analyzed by RDP Classifier (http://rdp.cme.msu.edu/) against the 16S rRNA database (Silva SSU128) using a confidence threshold of 0.7 [21]. The alpha diversity (Shannon, Simpson, Ace, Chao index) of the rumen microbiota based on OTU level was analyzed through MOTHUR (version 1.30.2). Beta diversity (principal co-ordinates analysis, PCoA) and other analyses were also conducted through QIIME (version 1.9.1). According to the abundance of microbial community, Kruskal-Wallis H test was used to conduct hypothesis testing for the microflora in the three groups, to evaluate the effects of ruminal bacterial abundance on the udder health status. False discovery rate (FDR) was used to conduct multiple testing for the correction of *P*-value. Hierarchical cluster analysis (HCA) and intergroup correlation analysis were performed using the OmicShare tools (http://www.omicshare.com/tools). Phylogenetic Investigation of Communities by Reconstruction of Unobserved States (PICRUSt), a technique that uses evolutionary modeling to predict metagenomes from 16S data and a reference genome database, was conducted to perform functional prediction analysis of differential microbiota. The analysis was operated using the PICRUSt software package (https://picrust.github. io/picrust/install.html#install) [21]. The accuracy of the prediction was assessed by the value of the nearest sequenced taxon index (NSTI) presenting as mean ± standard error of mean (SEM). The NSTI represents the average phylogenetic distance between the sequenced genomes with the closest relationship between OTU and all microorganisms in a sample. Therefore, the smaller of this value is, the more reliable of the prediction result will be [21].

### Metabolite extraction and LC-MS/MS analysis

A 100-μL rumen liquid sample was measured, and mixed with a 400-μL methanol: water (4:1, v/v) solution to extract the metabolites. The mixture was settled at −20 °C and treated by a high throughput tissue crusher Wonbio-96c (Wanbo Biotechnology Co., Ltd., Shanghai, China) at 50 Hz for 6 min, followed by a 30-s vortex, and then ultrasound at 40 kHz for 30 min at 5 °C. The samples were then placed at −20 °C for 30 min to precipitate proteins. After centrifugation under 13,000 × *g* at 4 °C for 15 min, the supernatant was carefully transferred to sample vials for LC-MS/MS analysis. Chromatographic separation of the metabolites was performed on an ExionLC™ AD system (AB Sciex, USA) equipped with an ACQUITY UPLC BEH C18 column (100 mm × 2.1 mm i.d., 1.7 μm; Waters, Milford, USA). The mobile phases consisted of 0.1% formic acid in water with formic acid (0.1%) (solvent A) and 0.1% formic acid in acetonitrile: isopropanol (1:1, v/v) (solvent B). The solvent gradient processing was in accordance with the study of Ogunade et al. [22]. The sample injection volume was 20 μL and the flow rate was set to 0.4 mL/min. The column temperature was maintained at 40 °C. During the period of analysis, all samples were stored at 4 °C. The UPLC system was coupled to a quadrupole-time-of-flight mass spectrometer (Triple TOF™ 5600+, AB Sciex, USA) equipped with an electrospray ionization (ESI) source operating in positive mode and negative mode. The optimal conditions were set as follows: source temperature, 500 °C; curtain gas (CUR), 30 psi; both Ion Source GS1 and GS2, 50 psi; ion-spray voltage floating (ISVF), − 4000 V in negative mode and 5000 V in positive mode, respectively; declustering potential, 80 V; a collision energy (CE), 20–60 V rolling for MS/MS. Data acquisition was performed with the Data Dependent Acquisition (DDA) mode. The detection was carried out over a mass range of 50–1000 m/z [22].

### Metabolomics data preprocessing and annotation

After LC-MS/MS analyses, the raw data were imported into the Progenesis QI 2.3 (Nonlinear Dynamics, Waters, USA) for peak detection and alignment. The preprocessing results generated a data matrix that consisted of the retention time (RT), mass-to-charge ratio (m/z) values, and peak intensity. Metabolic features detected at least 50% in any set of samples were retained. After filtering, minimum metabolite values were imputed for specific samples, in which the metabolite levels fell below the lower limit of quantitation and each metabolic features were normalized by sum. The internal standard was used for data quality control (QC) (reproducibility). Metabolic features that the relative standard deviation of QC > 30% were discarded. Following normalization procedures and imputation, statistical analysis was performed by log transformed data to identify significant differences in metabolite levels among the three groups. Mass spectra of these metabolic features were identified using the accurate mass, MS/MS fragments spectra and isotope ratio difference with searching in reliable biochemical databases as Human metabolome database (HMDB) (http://www.hmdb.ca/) and Metlin database (https://metlin.scripps.edu/). Concretely, the mass tolerance between the measured m/z values and the exact mass of the components of interest was ±10 ppm. Metabolites with MS/MS fragments score above 30 were considered as confidently identified. Otherwise, metabolites had only tentative assignments [23].

### Data analysis of metabolomics

A multivariate statistical analysis was performed using ropls (R packages) (version 1.6.2) (http://bioconductor.org/packages/release/bioc/html/ropls.html) from Bioconductor on Majorbio Cloud Platform (https://cloud.majorbio.com). Principle component analysis (PCA) using an unsupervised method was applied to obtain an overview of the metabolic data, and general clustering, trends, or outliers were visualized. All the variables of metabolites were scaled to unit-variances prior to conducting the PCA. Orthogonal partial least squares discriminate analysis (OPLS-DA) was used to determine the metabolic changes among the three groups. All of the metabolite variables were scaled to Pareto scaling prior to conducting the OPLS-DA. The model validity was evaluated by the model parameters R^2^ and Q^2^ to avoid the risk of over-fitting. Variable importance in the projection (VIP) was calculated in OPLS-DA model. *P*-values were estimated with paired Student’s *t*-test on single dimensional statistical analysis. Significance was declared at VIP value > 1 and FDR-adjusted *P*-value < 0.05. Differential metabolites between 2 groups were summarized, and mapped into their biochemical pathways through metabolic enrichment and pathway analysis based on the KEGG database search (http://www.genome.jp/kegg/). These metabolites were classified according to the involved pathways or the performed functions. Scipy (Python packages, https://docs.scipy.org/doc/scipy/) was used to identify significantly enriched pathways using Fisher’s exact test [22]. Receiver operator characteristic (ROC) curve of SPSS Statistics 22 (IBM, Chicago, USA) software was used to conduct the ROC analysis, which was used to evaluate the performance of biomarkers. Area under curve (AUC) was used to indicate the accuracy of the prediction. The higher the AUC value was and the closer to the upper left corner of the curve represented, the higher the prediction accuracy would be [23]. The Spearman correlation coefficient was calculated using the OmicShare tools, which were used to analyze the correlation between rumen microbes, metabolites and rumen fermentation parameters. The coefficients range from − 1.0 to 1.0, with variables close to 0 being irrelevant, and variables close to 1 or − 1 being strongly correlated.

### Statistical analysis

Data of the basic information of cows, milk yield and compositions, rumen fermentation variables and alpha diversity indices of the three groups were analyzed using one-way ANOVA and Student’s t-test of SPSS Statistics 22 (IBM, Chicago, USA) software. Significance was declared at *P* < 0.05, and 0.05 < *P* < 0.10 represented a tendency.

## Results

### Lactation and rumen fermentation

As the degree of mastitis increased, milk yield, milk fat and lactose contents were significantly reduced (*P* < 0.01), while SCC and milk protein were significantly increased (*P* < 0.01). There were no significant differences in pH-values (*P* = 0.214), the concentrations of RUN (*P* = 0.851), isobutyrate (*P* = 0.325), isovalerate (*P* = 0.213) and acetate to propionate ratio (A/P) (*P* = 0.111) in rumen fluid among the 3 groups. However, the LA, TVFAs (*P* < 0.01), acetate, propionate, butyrate and valerate (*P* < 0.001) contents in the CM group were significantly lower than those of the H and SM groups. In addition, the NH_3_-N concentration in rumen fluid tended to decline as the degree of mastitis increased (*P* = 0.061) (Table 1).

**Table 1:** Milk yield, compositions and rumen fermentation parameters in cows with different udder health status

Items	Experimental treatments			SEM	*P*-value
	H (*n* = 20)	SM (*n* = 20)	CM (*n* = 20)		
Lactation parameters					
Milk yield, kg/d	42.6^a^	32.7^b^	28.5^b^	1.43	< 0.010
Milk fat, %	3.58^a^	2.80^b^	2.14^c^	0.14	< 0.010
Milk protein, %	3.10^b^	3.06^b^	3.42^a^	0.10	< 0.010
Lactose, %	4.92^a^	3.95^b^	2.57^c^	0.18	< 0.010
Milk SCC, ×10^3^ cells/mL	42.8^c^	1002^b^	7053^a^	409	< 0.010
Rumen fermentation parameters					
pH	6.68	6.71	6.73	0.03	0.214
NH_3_-N, mg/dL	10.5	8.64	8.40	0.40	0.061
RUN, mg/dL	5.90	5.73	6.02	0.20	0.851
LA, mmol/L	0.86^b^	0.90^a^	0.80^c^	0.01	< 0.010
Acetate, mmol/L	64.5^a^	60.1^a^	41.0^b^	1.67	< 0.010
Propionate, mmol/L	27.7^a^	25.2^a^	19.2^b^	0.87	< 0.010
A/P	2.63	2.35	2.45	0.06	0.111
Isobutyrate, mmol/L	0.91	0.85	0.83	0.02	0.325
Butyrate, mmol/L	12.1^a^	12.1^a^	8.36^b^	0.36	< 0.001
Isovalerate, mmol/L	1.58	1.46	1.38	0.05	0.213
Valerate, mmol/L	1.54^a^	1.55^a^	1.15^b^	0.05	< 0.010
TVFAs, mmol/L	109^a^	105^a^	76.2^b^	3.00	< 0.010

Abbreviations: *SCC* Somatic cell counts, *NH*_*3*_*-N* Ammonia nitrogen, *RUN* Ruminal urea nitrogen, *LA* Lactic acid, *A/P* Acetate to propionate ratio, *TVFAs* Total volatile fatty acids, *H* Healthy, *SM* Subclinical mastitis, *CM* Clinical mastitis, *SEM* Standard error of mean. ^a, b, c^ values within a row, different letters denote differences between treatment combinations (*P* < 0.05)

### Rumen microbial diversity

**Fig. 1:**
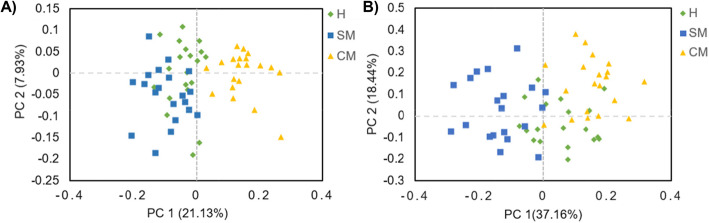
Principal co-ordinates analysis (PCoA) of ruminal microbiota based on a unweighted unifrac and b weighted normalized unifrac of OTUs among H, SM and CM cows. H, healthy; SM, subclinical mastitis; CM, clinical mastitis

A total of 3,072,603 high-quality sequences were generated from the 60 rumen fluid samples. We obtained 2973 OTUs ≥97% identity, with the Good’s coverage of H, SM and CM groups all above 98% (98.9% ± 0.0013, 98.7% ± 0.0020 and 98.6% ± 0.0011; mean ± standard deviation). Pan OTU was used to observe the increase of the total number of OTU with the increasing number of samples. The pan species curve exhibited that the curve gradually flattened as the number of samples increased, indicating that the sample size of this sequencing was sufficient (Additional file 1: Fig. S1). Similarly, the rarefaction curve showed that the rate of increase in OTU number slowed down with the increasing reads per sample, illustrating that the sequencing depth was adequate (Additional file 1: Fig. S2). Alpha diversity indices analysis suggested that there were significant differences in all the indexes at the OTU level, except for the Simpson index (*P* = 0.10). The Shannon (*P* = 0.02), Ace (*P* = 0.01) and Chao (*P* = 0.01) indexes in the SM and CM groups were significantly lower than those in the H group (Table 2). The PCoA based on unweighted Unifrac distance (*P* = 0.003) (Fig. 1a), weighted normalized Unifrac distance (*P* = 0.022) (Fig. 1b) and OTU classification level showed that ruminal microbiota in the CM group was distinguishable from the H and SM groups. The ruminal microbiota could also be slightly distinguished between H and SM groups but there were still some intersections.

**Table 2:** Rumen microbial alpha diversity indices in cows with different udder health status

Items	Experimental treatments			SEM	*P*-value
	H (*n* = 20)	SM (*n* = 20)	CM (*n* = 20)		
Shannon	5.65^a^	5.39^b^	5.44^b^	0.04	0.021
Simpson	0.01	0.02	0.02	0.00	0.103
Ace	1836^a^	1671^b^	1729^b^	22.3	0.011
Chao	1858^a^	1683^b^	1749^b^	23.4	0.010

Abbreviations: *H* Healthy, *SM* Subclinical mastitis, *CM* Clinical mastitis, *SEM* Standard error of mean. ^a, b^ values within a row, different letters denote differences between treatment combinations (*P* < 0.05)

### Rumen microorganism composition and structure

In total, 21 phyla and 356 genera of bacteria were obtained (Additional file 1: Table S4 and S5). Bacteria with relative abundance < 1% of all samples were classified as others. The dominating bacteria at phylum level in the 3 groups were Firmicutes (49.69 ± 0.57%), Proteobacteria (39.14 ± 0.42%), Bacteroidetes (4.65 ± 0.28%), Actinobacteria (3.22 ± 0.06%) and Cyanobacteria (1.29 ± 0.04%) (Additional file 1: Fig. S3a). At the genus level, *Prevotella_1* (23.89 ± 0.43%), *Succiniclasticum* (10.74 ± 0.11%), *Mollicutes_RF39* (8.77 ± 0.10%), *Pseudobutyrivibrio* (4.67 ± 0.27%), *Succinivibrionaceae_UCG-001* (3.82 ± 0.16%) and *Ruminococcaceae_NK4A214_group* (3.08 ± 0.25%) were the top 6 in all 3 groups (Additional file 1: Fig. S3b).

Hierarchical cluster analysis conducted for taxa at genus level (top 55 species in total relative abundance) further demonstrated the distribution of ruminal bacteria identified in different udder health status. Through average-linkage clustering techniques, the rumen microorganisms in the 60 dairy cows were aggregated into 3 clusters. Consistent with the PCoA results, ruminal bacteria in the CM group gathered together and separated from the H and SM groups. Although the H and the SM groups were generally differentiated, they still had some intersections, such as SM13, SM4, SM20, SM18 and SM9. There were 25 major bacteria in H group, including *Ruminococcus_2*, *Pseudoscardovia*, *Acetitomaculum*, *Bacteroidales*, *Bifidobacterium*, etc. Twelve bacteria, including *Succiniclasticum*, *Ruminiclostridium_9*, *vadinBE97*, *Prevotellaceae_UCG-001* etc., were detected in SM group. The CM group contained 17 genera of bacteria, such as *Herbaspirillum*, *Moraxella*, *Neisseriaceae*, etc. (Additional file 1: Fig. S4).

### Differentially abundant ruminal microbiota

Kruskal-Wallis H test was used to perform the analysis of significant differences of ruminal bacteria among the 3 groups. The significantly diverse microbes at genus level were assembled in 6 phylum, incorporating in Firmicutes, Bacteroidetes, Proteobacteria, and Cyanobacteria, etc. (Table 3 and Additional file 1: Fig. S5). The ruminal bacteria with high abundance in the CM group were mainly *Pseudobutyrivibrio*, Gastranaerophilales, *Moraxella*, Neisseriaceae, etc. In SM group, *Ruminiclostridium_9*, *Bacteroidales_UCG-001* and *Enterorhabdus*, etc., were most abundant. Comparing with the other two groups, *Lachnospiraceae*, *Prevotella_1*, *Mollicutes_RF39*, *Bifidobacterium*, etc., differed significantly in H group.

**Table 3:** The significantly differential bacteria (relative abundance) among cows with different udder health status

Items	Experimental treatments			SEM	*P*-value	FDR
	H (*n* = 20)	SM (*n* = 20)	CM (*n* = 20)			
Firmicutes	43.4^c^	48.3^b^	54.0^a^	11.3	0.001	0.030
Lachnospiraceae	0.48^a^	0.33^b^	0.23^bc^	0.27	0.002	0.039
*Syntrophomonas*	0.02^b^	0.01^c^	0.03^a^	0.00	0.003	0.041
*Lachnobacterium*	0.16^a^	0.09^b^	0.16^a^	0.09	0.002	0.035
*CAG-352*	0.19^a^	0.11^b^	0.12^a^	0.13	0.003	0.042
*Pseudobutyrivibrio*	0.51^b^	0.39^c^	0.66^a^	0.31	0.003	0.047
*Lachnospiraceae_UCG-006*	0.05^b^	0.04^c^	0.06^a^	0.02	0.003	0.047
*Ruminiclostridium_9*	0.13^b^	0.23^a^	0.10^b^	0.18	0.003	0.042
*Ornithinibacillus*	< 0.01^b^	0.01^a^	< 0.01^b^	0.00	0.003	0.043
Bacteroidetes	38.1^a^	36.4^b^	40.3^a^	6.94	0.002	0.035
Bacteroidales	0.08^a^	0.05^b^	0.05^b^	0.06	0.001	0.031
*Bacteroidales_UCG-001*	0.03^b^	0.05^a^	0.03^b^	0.03	0.002	0.038
*Prevotella_1*	25.1^a^	21.2^b^	17.3^b^	5.35	0.003	0.045
Proteobacteria	10.3^a^	8.60^b^	7.45^b^	10.8	0.002	0.032
*Moraxella*	< 0.01^b^	< 0.01^b^	0.04^a^	0.00	< 0.001	0.011
Neisseriaceae	0.02^b^	0.01^c^	0.05^a^	0.00	0.002	0.036
*Ralstonia*	0.01^b^	0.01^b^	0.05^a^	0.00	0.003	0.041
*Herbaspirillum*	0.004^b^	0.004^b^	0.013^a^	0.00	0.003	0.043
Pasteurellaceae	< 0.01^b^	< 0.01^b^	0.03^a^	0.00	0.002	0.036
Cyanobacteria	4.39^b^	3.73^c^	5.38^a^	0.63	0.002	0.033
Gastranaerophilales	0.21^c^	0.33^b^	0.44^a^	0.27	0.002	0.037
Tenericutes	1.11^a^	0.82^c^	0.92^b^	0.39	0.002	0.031
Izimaplasmatales	0.07^a^	0.04^b^	0.02^c^	0.03	0.003	0.045
*Mollicutes_RF39*	0.91^a^	0.66^c^	0.70^b^	0.35	0.003	0.047
Actinobacteria	0.85^a^	0.55^b^	0.38^c^	0.00	0.003	0.034
Enterorhabdus	0.08^b^	0.11^a^	0.05^c^	0.06	0.003	0.043
*Bifidobacterium*	0.49^a^	0.24^b^	0.12^c^	0.38	0.002	0.035

Abbreviations: *H* Healthy, *SM* Subclinical mastitis, *CM* Clinical mastitis, *SEM* Standard error of mean. ^a, b, c^ values within a row, different letters denote differences between treatment combinations (FDR-adjusted *P* < 0.05)

### Prediction of rumen microbial function

**Fig. 2:**
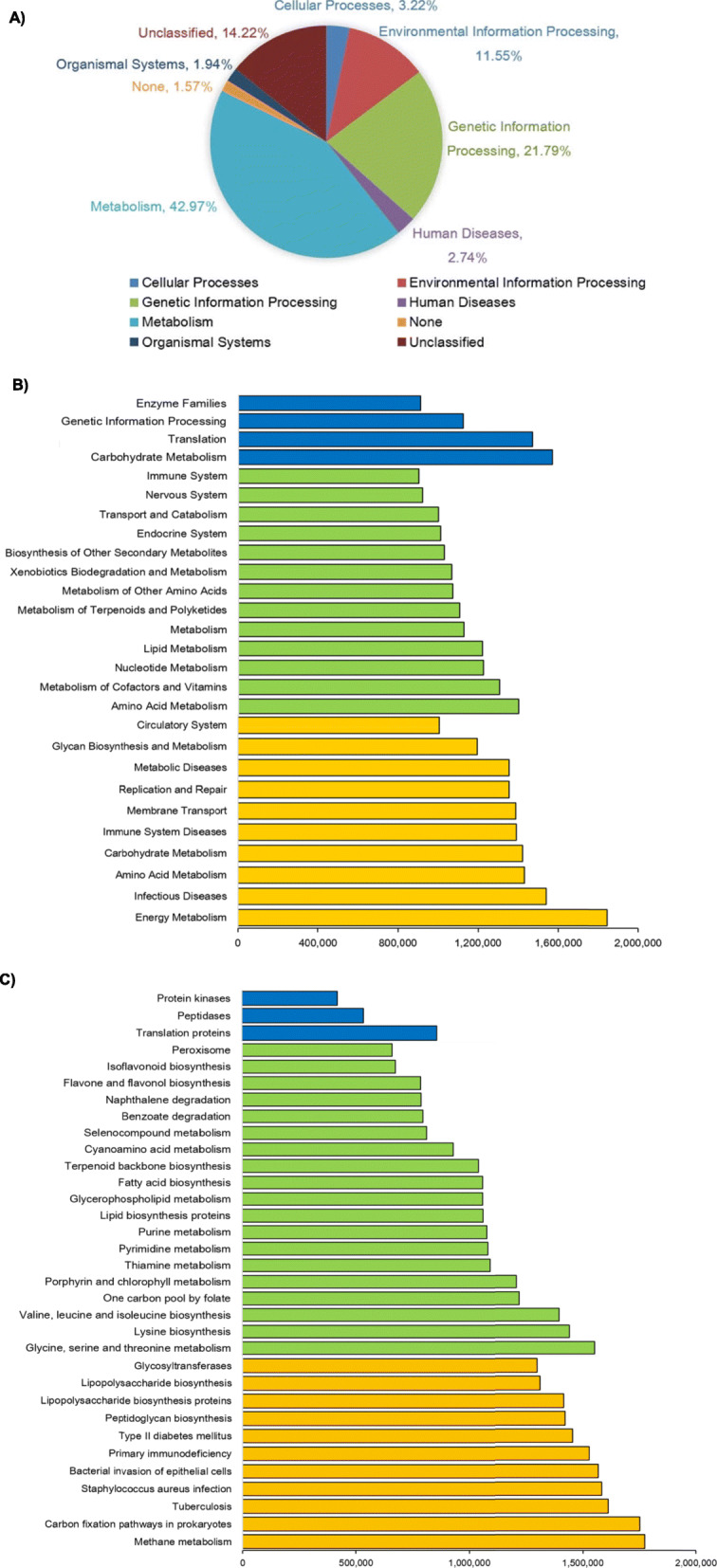
Phylogenetic investigation of communities by reconstruction of unobserved states (PICRUSt) functional prediction analysis for rumen microorganisms from H, SM and CM cows. **a** Superclass level. **b** Class level. **c** Subclass level. H, healthy; SM, subclinical mastitis; CM, clinical mastitis

To further speculate the contribution of these key flora, we attempted to predict the function of rumen microbiota in H (average NSTI = 0.33 ± 0.005), SM (average NSTI = 0.33 ± 0.007) and CM (average NSTI = 0.35 ± 0.005) groups by PICRUSt. At superclass level, 42.97% of the genes participated in metabolism, 21.79% belonged to genetic information processing, and 11.55% were involved with environment information processing. No significant difference was detected in the functions of rumen microorganisms among the 3 groups at level 1 (Fig. 2a). While at class level, a total of 39 KEGG pathways were noted, and 26 of them were different among H, SM and CM samples (FDR-adjusted *P* < 0.05). The abundance of flora related to amino acids coenzymes (FDR-adjusted *P* = 0.008) and vitamins (FDR-adjusted *P* = 0.004), nucleotides (FDR-adjusted *P* = 0.001) and lipid metabolism (FDR-adjusted *P* = 0.014), etc., were prominent in H group. Conversely, flora correlated with energy metabolism (FDR-adjusted *P* = 0.001), infection diseases (FDR-adjusted *P* = 0.001), immunity system diseases (FDR-adjusted *P* = 0.001), etc., accounted for a greater proportion in the CM group. In the SM group, the significantly different functional flora was less than H and CM groups, mainly focusing on translation (FDR-adjusted *P* = 0.003) and genetic information processing (FDR-adjusted *P* = 0.002) (Fig. 2 and Additional file 1: Table S6). At subclass level, among the 268 KEGG characteristics, 33 were different in abundance among the 3 groups (FDR-adjusted *P* < 0.05). Flora related to methane metabolism (FDR-adjusted *P* = 0.003), bacterial invasion and infection (FDR-adjusted *P* = 0.04), primary immunodeficiency (FDR-adjusted *P* = 0.037) and lipopolysaccharide biosynthesis (FDR-adjusted *P* = 0.043), etc., were significantly enriched in the CM group (Fig. 2c and Additional file 1: Table S7).

### Correlation between ruminal flora, and rumen fermentation and lactation performance

**Fig. 3:**
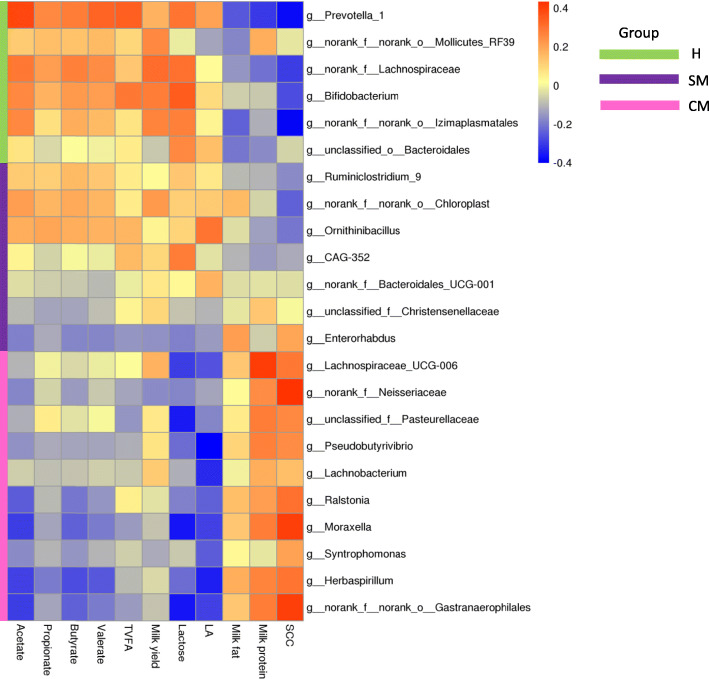
Correlation analysis between the differentially abundant bacteria and parameters of rumen fermentation as well as lactation performance. Each row represents a genus, each column represents a lactation performance or rumen fermentation parameter, and each cell represents a correlation coefficient between a lactation or rumen fermentation parameter and a bacterial genus. Red indicates the positive correlation, while the blue indicates the negative correlation

According to Spearman correlation coefficients, the rumen microbiota was associated with several rumen fermentation parameters, and milk yield and compositions (Fig. 3). Lachnospiraceae_UCG-006 (*r* = 0.409, FDR-adjusted *P* = 0.039) was positively correlated with milk protein. Neisseriaceae (r = 0.434, FDR-adjusted *P* = 0.008), *Moraxella* (*r* = 0.402, FDR-adjusted *P* = 0.005) and Gastranaerophilales (*r* = 0.402, FDR-adjusted *P* = 0.004) were positively correlated with milk SCC. *Bifidobacterium* (*r* = 0.349, FDR-adjusted *P* = 0.036) was positively associated with lactose, while Pasteurellaceae (*r* = − 0.354, FDR-adjusted *P* = 0.039), *Moraxella* (*r* = − 0.360, FDR-adjusted *P* = 0.045) and Gastranaerophilales (*r* = − 0.360, FDR-adjusted *P* = 0.043) were negatively associated with lactose. *Prevotella_1* (*r* = 0.384, FDR-adjusted *P* = 0.047) was positively correlated with acetate, but *Moraxella* (*r* = − 0.396, FDR-adjusted *P* = 0.045) and Gastranaerophilales (*r* = − 0.396, FDR-adjusted *P* = 0.044) were negatively correlated to acetate. *Herbaspirillum* (*r* = − 0.370, FDR-adjusted *P* = 0.043) and Gastranaerophilales (*r* = − 0.346, FDR-adjusted *P* = 0.043) were negatively associated with butyrate. In addition, *Pseudobutyrivibrio* (*r* = − 0.400, FDR-adjusted *P* = 0.02), *Herbaspirillum* (*r* = − 0.347, FDR-adjusted *P* = 0.04) and *Lachnobacterium* (*r* = − 0.333, FDR-adjusted *P* = 0.042) were negatively associated with LA (Additional file 1: Table S8).

### Comparative analysis of ruminal metabolites

**Fig. 4:**
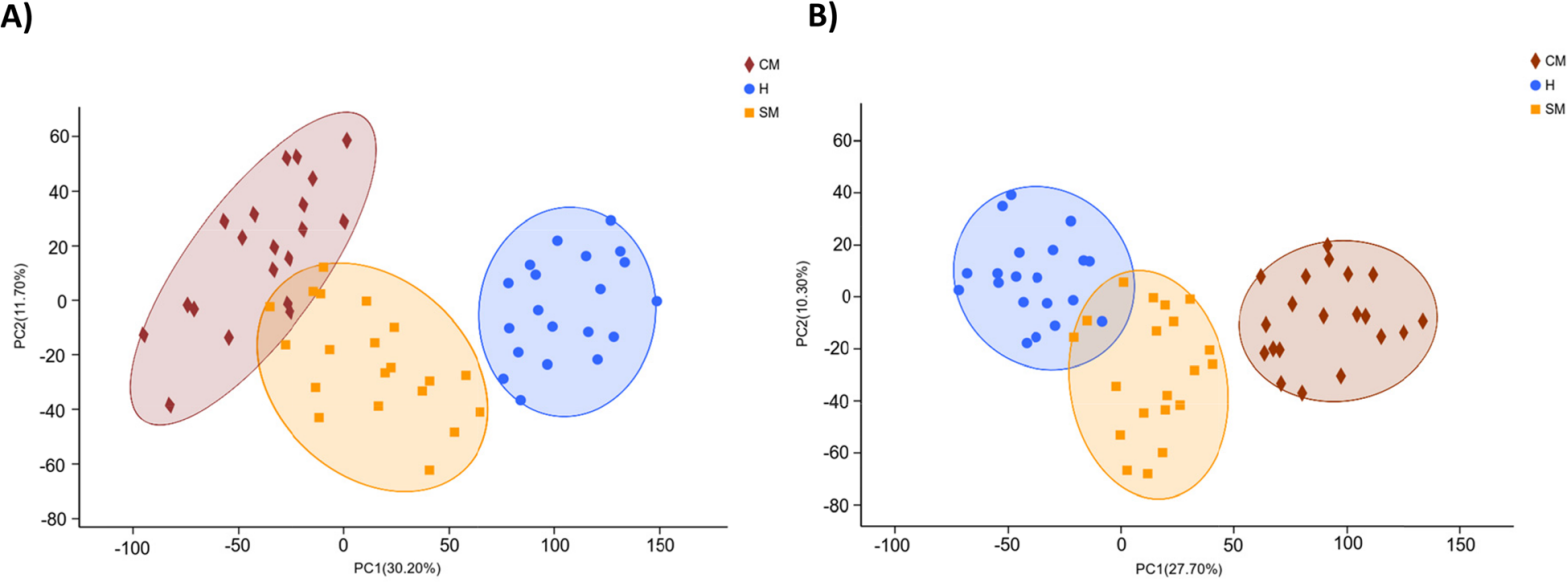
Principal component analysis (PCA) of the rumen metabolome of H, SM and CM cows in **a** positive and **b** negative ion mode. H, healthy; SM, subclinical mastitis; CM, clinical mastitis

The total ion chromatogram (TIC) of QC samples in positive and negative ion mode showed an overlap in the response intensity and retention time of each chromatographic peak (Additional file 1: Fig. S6), which indicated the high reliability of data quality. To exhibit the overall difference and the degree of the variability of rumen samples among and within the groups, unsupervised PCA was performed under positive and negative ionization modes, respectively (Fig. 4). Symbols representing ruminal metabolites in H, SM and CM groups could be basically separated. Further supervised OPLS-DA analysis of the data was performed in positive (Additional file 1: Fig. S7a, c and e) and negative ion modes (Additional file 1: Fig. S8a, c and e). Significant differences were detected between CM and H groups, SM and H groups, and CM and SM groups. Model parameter R^2^Y (cum) were all above 0.7 and Q^2^ (cum) were all above 0.5, indicating a good ability of model prediction. Model evaluation parameters of response permutation testing, Q^2^, were all below 0, suggesting that there was no overfitting of the model. The results illustrated that there were significant differences in the rumen metabolism under different udder health conditions of dairy cows (Additional file 1: Fig. S7b, d and f and Fig. S8b, d and f).

### Differential metabolites identification and evaluation

**Fig. 5:**
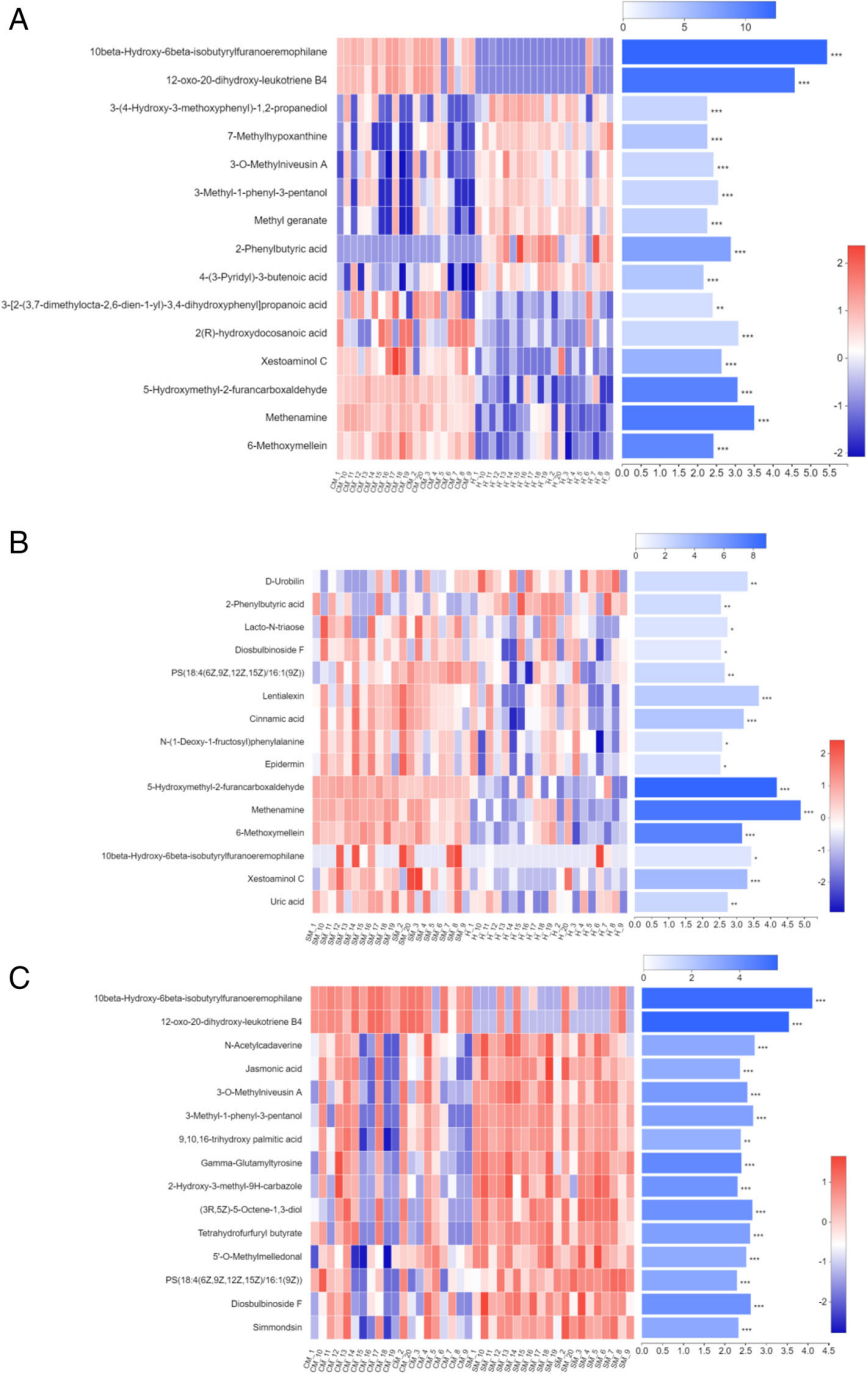
Significantly differential metabolites (top 15) in the rumen of dairy cows between **a** CM and H cows, **b** SM and H cows and **c** CM and SM cows based on variable importance in the projection (VIP) value under merger mode of positive and negative ion. H, healthy; SM, subclinical mastitis; CM, clinical mastitis. Each column represents a sample, each row represents a metabolite, and the color indicates the relative expression level of the metabolites in the samples. The right side is the metabolite VIP bar graph, the length of bar represents the contribution of the metabolites to the difference between the two groups (VIP > 1), the color of the bar means the FDR-adjusted *P*-value (*0.01 < FDR-adjusted *P* ≤ 0.05; **0.001 < FDR-adjusted *P* ≤ 0.01; *** FDR-adjusted *P* ≤ 0.001)

A total of 642 differential metabolites were selected including 384 and 258 in positive and negative ion mode, respectively, from the 60 rumen fluid samples. Under positive and negative ionization modes, the significantly differential metabolites (VIP > 1 and *P*-value < 0.05) among the 3 groups were shown in Additional file 1: Table S9-S14. Significantly differential metabolites (top 15) in the rumen between CM and H groups, SM and H groups, and CM and SM groups are shown in Fig. 5a, b and c respectively. Comparing to H group, 10beta-hydroxy-6beta-isobutyrylfuranoeremophilane (FDR-adjusted *P* < 0.001) and 12-oxo-20-dihydroxy-leukotriene B4 (FDR-adjusted *P* < 0.001) in CM group increased 12.23- and 16.36-fold, respectively. Meanwhile, these two in CM group increased 2.88 and 3.64-fold, respectively, compared with SM group (Table 4). Methenamine (FDR-adjusted *P* < 0.001), 5-hydroxymethyl-2-furancarboxaldehyde (5-HMF) (FDR-adjusted *P* < 0.001) and 6-methoxymellein (FDR-adjusted *P* < 0.001) in CM and SM groups were both higher than those in H group. Besides, xestoaminol C (FDR-adjusted *P* = 0.016), lentialexin (FDR-adjusted *P* = 0.031) and cinnamic acid (FDR-adjusted *P* = 0.042) in SM group were increased compared to H group. Less abundance of 2-phenylbutyric acid (2-PBA) (FDR-adjusted *P* < 0.001) was detected in CM group compared with H group. In CM group, N-acetylcadaverine (FDR-adjusted *P* = 0.020) and (3R, 5Z)-5-octene-1,3-diol (FDR-adjusted *P* = 0.011) were reduced compared with SM group (Table 4 and Fig. 5).

**Table 4:** HMDB compound classification of differential metabolites in cows with different udder health status

HMDB superclass	HMDB class^a^	HMDB subclass^a^	Metabolites	VIP^b^	FC^c^	*P*-value	FDR
CM/H							
Lipids and lipid-like molecules	Prenol lipids	Sesquiterpenoids	10beta-Hydroxy-6beta-isobutyrylfuranoeremophilane	5.27	12.23	3.41 × 10^− 13^	3.88 × 10^− 10^
	Fatty acyls	Eicosanoids	12-Oxo-20-dihydroxy-leukotriene B4	4.53	16.36	3.82 × 10^− 12^	5.97 × 10^− 9^
Organoheterocyclic compounds	Triazinanes	1,3,5-Triazinanes	Methenamine	3.74	4.08	1.40 × 10^−11^	4.92 × 10^−9^
	Carbonyl compounds	Aldehydes	5-Hydroxymethyl-2-furancarboxaldehyde	3.07	4.89	6.11 × 10^−11^	1.86 × 10^−8^
	Benzopyrans	2-Benzopyrans	6-Methoxymellein	2.51	3.30	1.63 × 10^−10^	4.13 × 10^−8^
Phenylpropanoids and polyketides	Phenylpropanoic acids	–	2-Phenylbutyric acid	2.83	0.26	2.24 × 10^−8^	4.37 × 10^−6^
SM/H							
Organoheterocyclic compounds	Triazinanes	1,3,5-Triazinanes	Methenamine	5.15	4.85	1.07 × 10^−8^	6.95 × 10^− 6^
	Carbonyl compounds	Aldehydes	5-Hydroxymethyl-2-furancarboxaldehyde	4.76	4.80	1.33 × 10^−9^	2.02 × 10^−6^
	Benzopyrans	2-Benzopyrans	6-Methoxymellein	3.37	3.23	5.64 × 10^−8^	2.57 × 10^−5^
Phenylpropanoids and polyketides	Cinnamic acids and derivatives	Cinnamic acids	Cinnamic acid	2.75	1.27	4.61 × 10^−4^	0.042
Lipids and lipid-like molecules	Fatty acyls	Fatty alcohols	Lentialexin	3.09	1.48	9.70 × 10^−4^	0.041
–	–	–	Xestoaminol C	3.57	1.21	8.78 × 10^−5^	0.016
CM/SM							
Lipids and lipid-like molecules	Prenol lipids	Sesquiterpenoids	10beta-Hydroxy-6beta-isobutyrylfuranoeremophilane	4.09	2.88	4.25 × 10^−6^	0.003
	Fatty acyls	Eicosanoids	12-Oxo-20-dihydroxy-leukotriene B4	3.24	3.64	2.63 × 10^−6^	0.002
	Fatty acyls	Fatty alcohols	(3R,5Z)-5-Octene-1,3-diol	2.66	0.51	9.97 × 10^−5^	0.011
Organic acids and derivatives	Carboxylic acids and derivatives	Carboxylic acid derivatives	N-Acetylcadaverine	2.71	0.61	6.96 × 10^−4^	0.020

^a^–: no pathway information. ^b^ VIP: the contribution value of metabolites to the difference between the two groups (VIP > 1). ^c^ FC: FC > 1 represents the upregulated compounds, while FC < 1 represents the downregulated compounds

Abbreviations: *CM/H* The clinical mastitis group versus to the healthy group, *SM/H* The subclinical mastitis group versus to the healthy group, *CM/SM* The clinical mastitis group versus to the subclinical mastitis group, *HMDB* Human metabolome database, *VIP* Variable importance in the projection, *FC* Fold change, *FDR* False discovery rate

**Fig. 6:**
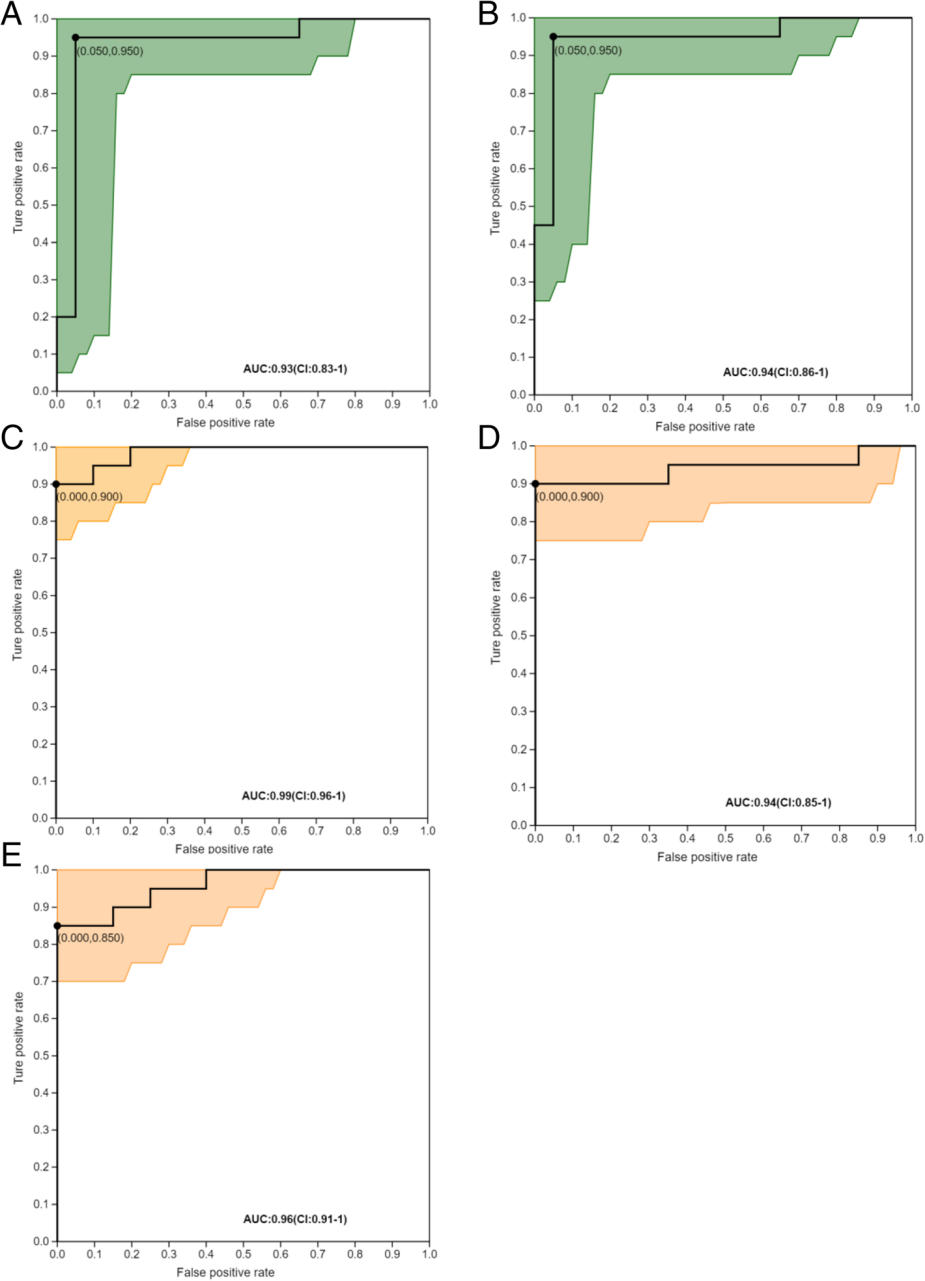
Receiver operating characteristic (ROC) curves of metabolites in the prediction and diagnosis of mastitis. ROC curve reflects the relationship between sensitivity and specificity. The X-axis is false positive rate (specificity). The closer the X-axis to zero, the higher the accuracy will be. The Y-axis is true positive rate (sensitivity). The larger the Y-axis is, the better the accuracy is. The area under curve (AUC) is used to indicate the accuracy of prediction. AUC value is between 0 and 1, the higher the AUC value is, the higher the accuracy of prediction is. **a** 10beta-Hydroxy-6beta-isobutyrylfuranoeremophilane; **b** 12-Oxo-20-dihydroxy-leukotriene B4; **c** Methenamine; **d** 5-Hydroxymethyl-2-furancarboxaldehyde; **e** 6-Methoxymellein

To evaluate the diagnostic value for BM of these key differential metabolites in the rumen, receiver operator characteristic (ROC) analysis was performed, which reflected the relationship between false positive rate (X-axis) and true positive rate (Y-axis) (Fig. 6). In the current study, the AUC values of the 5 key differential metabolites, including 10beta-hydroxy-6beta-isobutyrylfuranoeremophilane, 12-Oxo-20-dihydroxy-leukotriene B4, methenamine, 5-HMF and 6-methoxymellein, were all above 0.9, and the ROC curves were all close to the upper left corner. This indicated that these 5 differential metabolites in the rumen screened in the present study were of high value to be served as the biomarkers for bovine mastitis.

### Differential metabolites classification and metabolic pathways enrichment analysis

Compound classification of differential metabolites in the rumen fluid samples were processed based on comparisons with the HMDB. Differentially clustered metabolites belonging to lipid-like molecules, organ heterocyclic compounds, and organic acids and derivatives compounds accounted for the top 3 among the 3 groups (Table 4 and Additional file 1: Fig. S9).

Furthermore, the enrichment of metabolic pathway analysis showed that, 10beta-hydroxy-6beta-isobutyrylfuranoeremophilane was enriched in limonene and pinene degradation pathway (FDR-adjusted *P* = 0.008). 12-Oxo-20-dihydroxy-leukotriene B4 was enriched in arachidonic acid metabolism pathway (FDR-adjusted *P* = 0.026). Besides, methenamine was the intermediate product of formaldehyde biosynthesis (FDR-adjusted *P* = 0.036). 5-Hydroxymethyl-2-furancarboxaldehyde participated in the furfural degradation (FDR-adjusted *P* = 0.032). 6-Methoxymellein was enriched in the biosynthesis of plant secondary metabolites pathway (FDR-adjusted *P* = 0.045). And 2-phenylbutyric acid was enriched in the butanoate metabolism pathway (FDR-adjusted *P* = 0.042) (Table 5).

**Table 5:** Metabolic pathway enrichment analysis of significantly differential metabolites among health, subclinical mastitis and clinical mastitis cows

Groups	Metabolites	KEGG pathway	*P*-value	FDR
CM/H	10beta-Hydroxy-6beta-isobutyrylfuranoeremophilane	Limonene and pinene degradation	3.09 × 10^−6^	0.008
	12-Oxo-20-dihydroxy-leukotriene B4	Arachidonic acid metabolism	1.94 × 10^− 5^	0.026
	Methenamine	Formaldehyde biosynthesis	3.25 × 10^−5^	0.036
	5-Hydroxymethyl-2-furancarboxaldehyde	Furfural degradation	3.03 × 10^−5^	0.032
	6-Methoxymellein	Biosynthesis of plant secondary metabolites	3.39 × 10^−4^	0.045
	2-Phenylbutyric acid	Butanoate metabolism	6.62 × 10^−4^	0.042
SM/H	Methenamine	Formaldehyde biosynthesis	7.79 × 10^−5^	0.037
	5-Hydroxymethyl-2-furancarboxaldehyde	Furfural degradation	2.54 × 10^−4^	0.036
	6-Methoxymellein	Biosynthesis of plant secondary metabolites	3.64 × 10^−4^	0.047
	Cinnamic acid	Phenylpropylene degradation	7.58 × 10^−4^	0.058
	Lentialexin	Biosynthesis of plant secondary metabolites	0.003	0.178
CM/SM	10beta-Hydroxy-6beta-isobutyrylfuranoeremophilane	Limonene and pinene degradation	6.37 × 10^−5^	0.025
	12-Oxo-20-dihydroxy-leukotriene B4	Arachidonic acid metabolism	7.34 × 10^−5^	0.029
	(3R,5Z)-5-Octene-1,3-diol	Fat hydrolysis	9.54 × 10^−4^	0.063
	N-Acetylcadaverine	Lysine degradation	1.06 × 10^−4^	0.034

Abbreviations: *CM/H* The clinical mastitis group versus to the healthy group, *SM/H* The subclinical mastitis group versus to the healthy group, *CM/SM* The clinical mastitis group versus to the subclinical mastitis group, *FDR* False discovery rate

### Correlations between ruminal metabolites, and lactation and rumen fermentation parameters

**Fig. 7:**
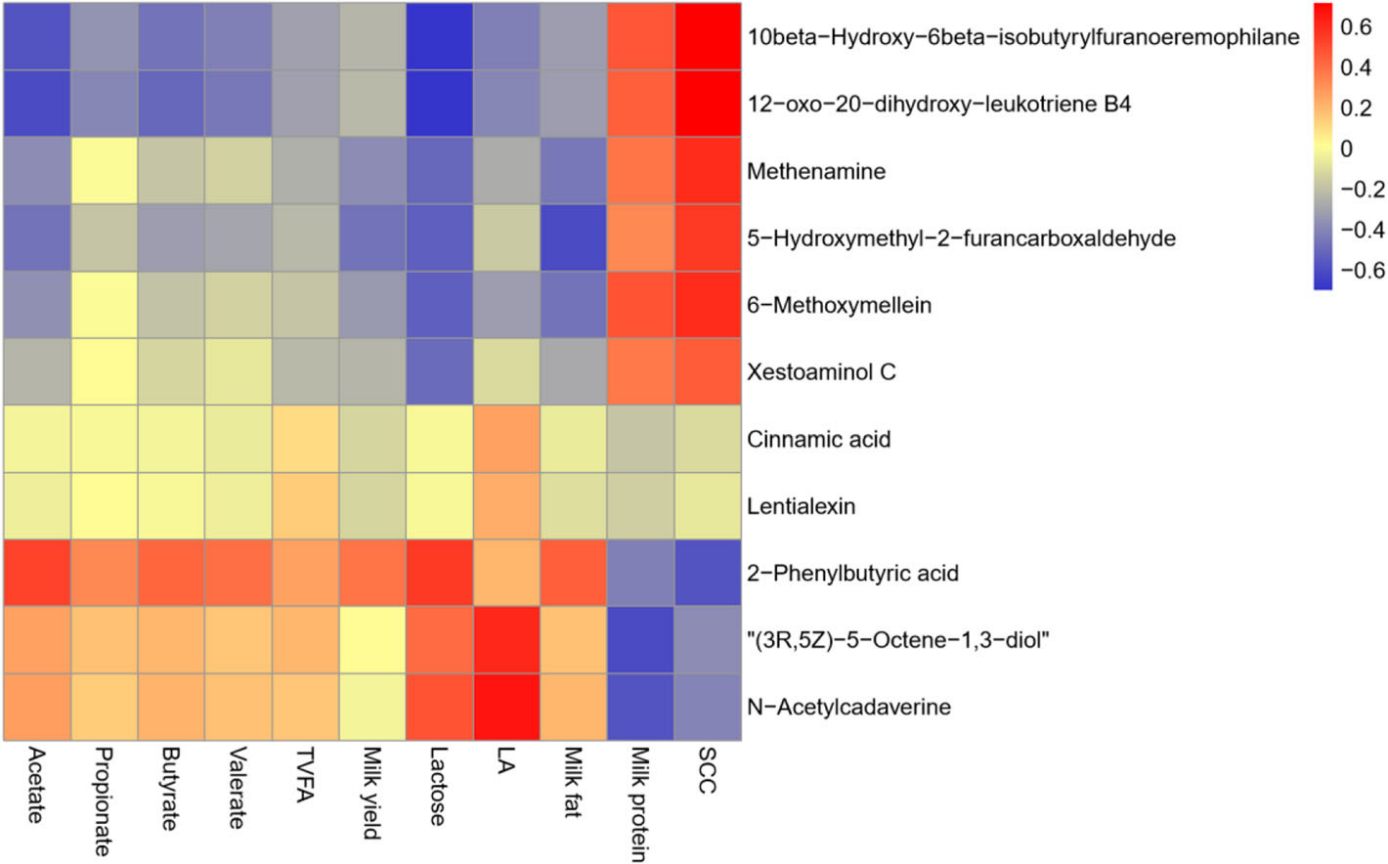
Correlation analysis of differential metabolites and index of lactation and rumen fermentation. Each row represents a metabolite, each column represents a lactation or rumen fermentation parameter. Red means positive correlation, while blue means negative correlation

Based on Spearman correlation coefficients and Euclidean distance matrix, we observed significant correlations between significantly differential metabolites, and lactation as well as rumen fermentation parameters (Fig. 7). 10beta-Hydroxy-6beta-isobutyrylfuranoeremophilane (*r* = − 0.584, FDR-adjusted *P* < 0.001) and 12-Oxo-20-dihydroxy-leukotriene B4 (*r* = 0.601, FDR-adjusted *P* < 0.001) were negatively correlated to acetate, whereas the 2-PBA (*r* = 0.520, FDR-adjusted *P* < 0.001) was positively correlated to acetate. Meanwhile, 10beta-hydroxy-6beta-isobutyrylfuranoeremophilane (*r* = − 0.472, FDR-adjusted *P* = 0.046) and 12-oxo-20-dihydroxy-leukotriene B4 (*r* = − 0.504, FDR-adjusted *P* = 0.002) were also negatively associated with butyrate. Besides, (3R, 5Z)-5-octene-1, 3-diol (*r* = 0.612, FDR-adjusted *P* < 0.001) and N-acetylcadaverine (*r* = 0.670, FDR-adjusted *P* < 0.001) were positively associated with LA. 10beta-Hydroxy-6beta-isobutyrylfuranoeremophilane (*r* = − 0.700, FDR-adjusted *P* < 0.001), 12-Oxo-20-dihydroxy-leukotriene B4 (*r* = − 0.688, FDR-adjusted *P* < 0.001), 5-HMF (*r* = − 0.537, FDR-adjusted *P* < 0.001) and 6-methoxymellein (*r* = − 0.542, FDR-adjusted *P* < 0.001) were negatively associated with lactose, while 2-PBA was positively associated with lactose (*r* = 0.547, FDR-adjusted *P* < 0.001). Furthermore, 5-HMF was negatively associated with milk fat (*r* = − 0.604, FDR-adjusted *P* < 0.001). (3R, 5Z)-5-Octene-1, 3-diol (*r* = − 0.606, FDR adjusted *P* < 0.001) and N-acetylcadaverine (*r* = − 0.582, FDR-adjusted *P* < 0.001) were negatively correlated to milk protein. 10beta-Hydroxy-6beta-isobutyrylfuranoeremophilane (*r* = 0.716, FDR-adjusted *P* < 0.001), 12-oxo-20-dihydroxy-leukotriene B4 (*r* = 0.712, FDR-adjusted *P* < 0.001), methenamine (*r* = 0.603, FDR-adjusted *P* < 0.001), 5-HMF (*r *= 0.550, FDR-adjusted *P* < 0.001) and 6-methoxymellein (r = 0.600, FDR adjusted *P* < 0.001) were positively associated with milk SCC, but 2-PBA was negatively associated with milk SCC (*r* = − 0.573, FDR-adjusted *P* < 0.001) (Additional file 1: Table S15).

### Associations between BM-linked rumen microbes and metabolites

**Fig. 8:**
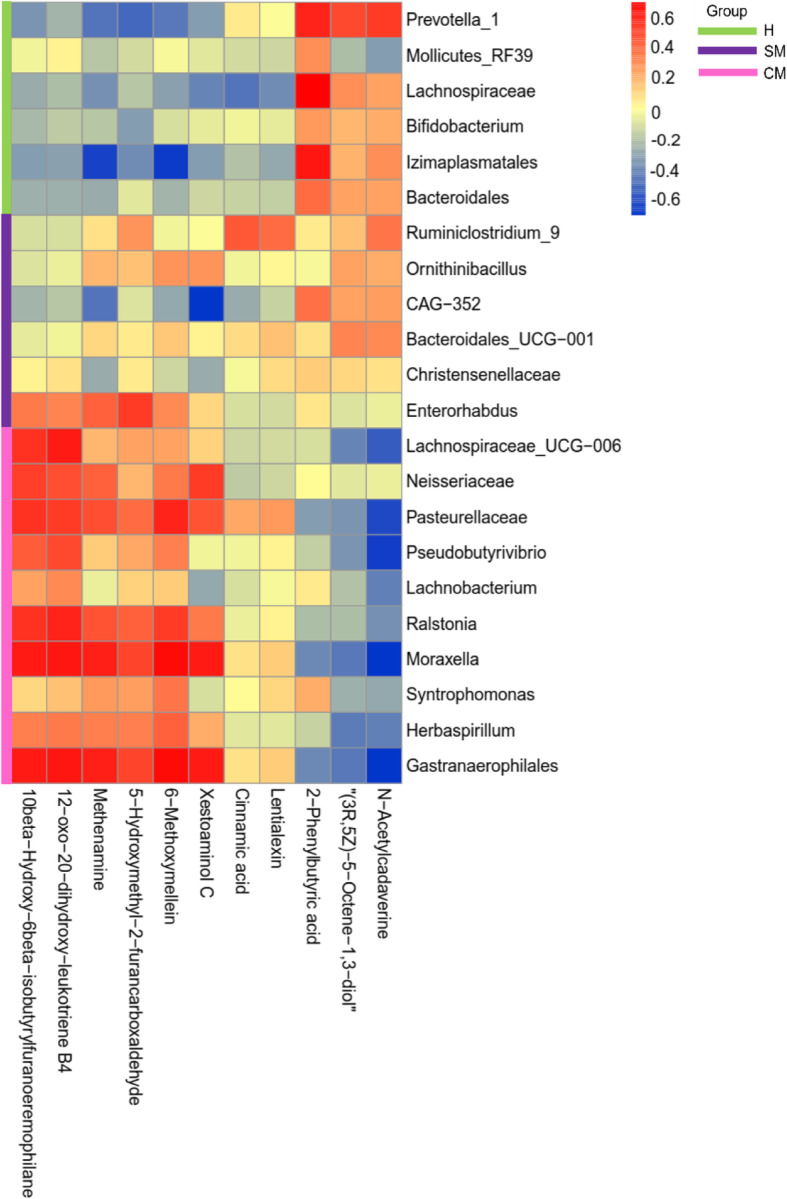
Correlation analysis of differential bacteria genus and differential metabolites in rumen samples. Each row represents a metabolite, each column represents a bacterium. Red means positive correlation, while blue means negative correlation

The Spearman correlation coefficients were calculated to reflect the correlations between significantly differential bacteria and metabolites via heat map (Fig. 8). In CM group, *Lachnospiraceae_UCG-006* (*r* = 0.493, FDR-adjusted *P* = 0.038), Pasteurellaceae (*r* = 0.565, FDR-adjusted *P* = 0.043)*, Moraxella* (*r* = 0.599, FDR-adjusted *P* = 0.033)*,* and Gastranaerophilales (*r* = 0.589, FDR-adjusted *P* = 0.031) were positively related to 10beta-hydroxy-6beta-isobutyrylfuranoeremophilane. *Lachnospiraceae_UCG-006* (*r* = 0.542, FDR-adjusted *P* = 0.042), *Moraxella* (*r* = 0.574, FDR-adjusted *P* = 0.038) and Gastranaerophilales (*r* = 0.561, FDR-adjusted *P* = 0.037) were also positively related to 12-oxo-20-dihydroxy-leukotriene B4. Pasteurellaceae (*r* = − 0.556, FDR-adjusted *P* = 0.036), *Pseudobutyrivibrio* (*r* = − 0.588, FDR-adjusted *P* = 0.01), *Moraxella* (*r* = − 0.595, FDR-adjusted *P* = 0.025) and Gastranaerophilales (*r* = − 0.585, FDR-adjusted *P* = 0.025) were negatively correlated with N-acetylcadaverine. In SM group, Enterorhabdus (*r* = 0.527, FDR-adjusted *P* = 0.047) were positively associated with 5-HMF. The preponderant strains in H group, including *Prevotella_1* (*r* = 0.572, FDR-adjusted *P* = 0.040), Lachnospiraceae (*r* = 0.578, FDR-adjusted *P* = 0.042), and Izimaplasmatales (*r* = 0.545, FDR-adjusted *P* = 0.042) were positively correlated with 2-PBA, while Izimaplasmatales was negatively associated with methenamine (*r* = − 0.577, FDR-adjusted *P* = 0.041) and 6-methoxymellein (*r* = − 0.499, FDR-adjusted *P* = 0.002) (Additional file 1: Table S16).

## Discussion

Milk SCC, CMT method and udder clinical symptoms were used to determine the udder health status in the current study. Screening out cows with H, SM and CM through comprehensive analysis improved the prediction accuracy of disease incidence and progression [24, 25]. Besides, unlike the work of Zhong et al. [26], we increased the difference gap of SCC levels among the 3 groups and considered udder clinical symptoms when grouping to further investigate the correlation between BM and changes in rumen microflora and metabolites.

Consistent with expectations, the severity of BM was accompanied by a decline in lactation performance. The significant reduction in milk yield, milk fat and lactose contents was a reflection of the malfunction of the milk synthesis system in the mammary gland [1, 3]. However, the content of total milk protein in CM group was the highest among the 3 groups. Generally, milk yield is inversely proportional to milk protein [26]. In line with the report by Rhone et al. [3], decreased milk yield resulted in increased dry matter content and the total protein rate in milk. In addition, raised SCC would increase the permeability of the blood-milk barrier that bring about serum proteins, immunoglobulins, and lactoferrin into the milk, which consequently increased the total amount of milk proteins [1]. Furthermore, the inner-environment of rumen, including microbial compositions and their fermented products, such as VFAs, also played decisive roles in milk yield and compositions [15]. In the current study, the content of ruminal VFAs and LA in CM group were significantly decreased, illustrating that the rumen environment was abnormal during acute BM. Besides, the reduced milk fat synthesis in CM may be due to the decreased acetate and butyrate which could affect the expression of acetyl-coenzyme A carboxylase and fatty acid synthase and thus changed the synthesis of milk fat [27]. The less propionate in the rumen of CM cows limited the production of lactose, due to the restricted supply of glucose [12, 28]. The above results suggested that the alteration of rumen fermentation pattern in cows affected by CM was related to the loss of milk yield and poor milk quality, which may be correlated with the changes in rumen microbial structure and metabolic activity.

Except for fermentation products, rumen microorganisms also have the ability to induce systemic inflammation [5, 29]. Shifts in microbial populations associated with dysbiosis in rumen cause translocation and systemic dissemination of increased concentrations of potentially toxic and inflammatory substances, such as lipopolysaccharide [29]. In the present study, the main phyla of rumen microorganisms, Firmicutes, Proteobacteria and Bacteroidetes in cows with H, SM and CM were not significantly different, which suggested that the bacterial community at phyla level with crucial function in rumen was relatively stable in cows under different udder health status [26]. Subsequent microbial HCA and PCoA analysis verified that only partial differences existed in rumen microbes from H and SM cows, which consisted with the results of Zhong et al. [26]. However, the rumen bacteria structure of CM cows was significantly distinguished from the other two groups. In the present study, the rumen microflora imbalance during IMI was characterized by a large decrease in symbiotic bacteria and substantially increase in pathogens, which may be associated with the intestinal or oral inflammation. In the changed rumen flora during CM, Pasteurellaceae has been proved to cause infection through direct or indirect contact, which colonized in the oral cavity of livestock [30]. Another changed pathogen from oral cavity was Neisseriaceae which was associated with a high risk of gastric cancer [31]. *Lachnospiraceae_UCG-006* has been reported with close correlation with intestinal inflammation and diabetes [32], and so is Gastranaerophilales [33]. Besides, Ferreira et al. [34] also found that Gastranaerophilales was significantly enriched in milk from CM cows. Additionally, *Moraxella* is a main pathogen causing respiratory infections, laryngitis and enteritis, and also had strong correlation with rumen wall damage [29]. *Ralstonia* was reported to induce infected hemoperitoneum in human [35], while *Herbaspirillum* could invade human and livestock through the digestive tract, and further cause infection in immunocompromised individuals [36, 37]. However, in CM group, the abundance of *Pseudobutyrivibrio* which was a probiotic in rumen [38] was elevated. It is speculated that, when inflammation occurs, a large number of *Pseudobutyrivibrio* in rumen produce butyrate to enhance the function of intestinal mucosal immune barrier, further preventing bacteria and their metabolites to enter the bloodstream and inhabiting the associated inflammation, which might exert a beneficial role [38]. On the other hand, *Ornithinibacillus* and *Enterorhabdus* were identified in SM group. Although partial *Bacillus* in gastrointestinal tract was harmless, a mass of *Ornithinibacillus* in water could cause gastroenteritis [39]. *Enterorhabdus* is a common conditional pathogen that can induce intestinal infections. Meanwhile, it is also significantly enriched in milk from mastitis cows [40]. Overall, the above microorganisms, which significantly increased in the cows’ rumen during CM, have been reported with certain pathogenicity in humans or other animals, however their pathogenicity in the rumen of cows still needs further investigation.

In contrast, the high-abundant ruminal microbiota in the H and SM groups mostly belonged to non-pathogenic symbiotic bacteria and intestinal probiotics, such as *Prevotella_1*, *Bifidobacterium*, Lachnospiraceae and *Mollicutes_RF39* in H group, and *Ruminiclostridium_9* in SM group. Jami and Mizrahi [41] found that Mollicutes was one of the most abundant phyla in rumen, just after *Bacteroides* and *Pachyphyta*. Similarly, Lachnospiraceae in the bovine milk samples obtained from the healthy quarters, was considered as part of the healthy milk core microbiota [42]. *Ruminiclostridium_9* could degrade the mucus protein of intestinal epithelial cell, produce SCFAs, and maintain the stability of the intestinal environment [43], although its functions in rumen were still unknown.

Commensal dysbiosis in mammary gland promoted early inflammation within the udder. Further, intestinal flora dysbiosis induced by fecal bacteria transplantation could also trigger the inflammatory cells dissemination into the udder [44]. Thus, we attempted to preliminarily predict the potential function of differential ruminal bacteria among the three groups in the current study. The PICRUSt is a computational approach to predict the functional composition of a metagenome using marker gene data and a database of reference genomes [21]. However, the accuracy of PICRUSt (NSTI value) in the three groups was not optimal in the present study. Langille et al. [21] reported that PICRUSt could only predict a limited number of taxa based on the 16S gene copy numbers. Furthermore, the NSTI could make accurate predictions for non-animal related samples, but poor for animal related samples.

In the present study, numerous increased ruminal microorganisms in the CM group had positive association with milk SCC. These microorganisms, as discussed above, were mostly related to oral cavity and intestinal inflammation. Usually, the elevated SCC in milk was due to pathogens invaded and colonized in udder from external environment. However, increasing studies confirmed the existence of the entero-mammary pathway [26]. In fact, gastrointestinal microorganisms can be transmitted to udder through lymphatic vessels and peripheral blood circulation during lactation [45]. Addis et al. [45] found that the abundance of *Ruminococcus* in milk from mastitis cows was significantly higher than that of healthy cows. The destruction of the integrity of rumen epithelial-vascular endothelial barrier due to ruminal damage facilitates the entry of pathogens or bacterial antigens into the portal vein and thereby causes systemic inflammation in other organs, including mammary gland [29]. The above studies together with our results triggered speculation on the relationships between microbial endogenous pathways and the occurrence of mastitis.

Besides, various systemic inflammatory disease (arthritis, spondylitis, mastitis, etc.) are related with the absence of SCFAs-producing bacteria in gastrointestinal tract [5, 14]. In the current study, abundant *Prevotella_1*, Lachnospiraceae and *Bifidobacterium* in H group were observed which could ferment starch and glucose to produce acetate, propionate and butyrate. Meanwhile, *Ruminiclostridium_9* (cellobiose-degrading bacteria) [43] and *Ornithinibacillus* (lactic acid-producing bacteria) in the SM group can also produce SCFAs and LA [39]. This may explain their positive relationships with VFAs and LA in rumen. However, they were significantly reduced in the CM group. The SCFAs in rumen provide energy substrates for lactation, meanwhile, inhibit the proliferation of pathogens in the gastrointestinal tract [46]. Propionate can enhance the resistance to colonization of *Salmonella* by disrupting the pH stability of bacteria [46]. Butyrate can limit the growth of pathogenic Enterobacteriaceae [47].

Metabolomics data showed that, the significantly changed metabolites in the rumen were mostly associated with inflammation and antibacterial activity. Five significantly increased ruminal metabolites were identified during mastitis, including 12-oxo-20-dihydroxy-leukotriene B4, 10beta-hydroxy-6beta-isobutyrylfuranoeremophilane, 5-HMF, methenamine, and 6-Methoxymellein. 12-oxo-20-dihydroxy-leukotriene B4 is an isomer of leukotriene B4 (LTB4), and in this study, it was significantly increased in the CM group compared to the H group, which was consistent with the elevated LTB4 found in CM cows by Boutet et al. [48]. Correlation analysis showed that ruminal bacteria, including Gastranaerophilales, *Lachnospiraceae_UCG-006* and *Moraxella* etc., were positively correlated with LTB4 during the CM. The lipoxygenase secreted by the pathogens initiates the conversion of arachidonic acid to LTB4, which in turn triggers an inflammatory response. Furthermore, Polymorphonuclear leukocytes (PMNLs) was the main cell type in the increased SCC during mastitis, and the LTB4 was the key chemokine for PMNLs in inflamed tissue [48], which agrees with the strongly positive correlation between 12-oxo-20-dihydroxy-leukotriene B4 and milk SCC in the current study. 10beta-Hydroxy-6beta-isobutyrylfuranoeremophilane is a kind of sesquiterpenoids, participating in the degradation of limonene and pinene, both of which were volatile substances with anti-inflammatory and antibacterial effects [49]. The significantly increased 10beta-hydroxy-6beta-isobutyrylfuranoeremophilane in the CM group compared to the H group may thus accelerate the limonene and pinene degradation and weaken their antibacterial and anti-inflammatory activity. However, reports on 10beta-hydroxy-6beta-isobutyrylfuranoeremophilane in mastitis are quite limited. High concentrations of 5-HMF are cytotoxic by inducing DNA damage [50]. Additionally, 5-HMF could also induce the production of pro-inflammatory factors, such as TNF-α and IL-1β [51]. Both 6-methoxymellein [52] and methenamine [53] were reported to inhibit the growth of tissue cells or produce harmful metabolites, but also had certain antibacterial activities. The correlation analysis showed that methenamine and 6-methoxymellein were positively associated with milk SCC and ruminal pathogens. Therefore, the increase of methenamine and 6-Methoxymellein during SM is possibly due to either the increase of harmful metabolites produced by pathogens, or a large number of symbiotic microbiota in rumen with resistance to potential pathogens through competitive rejection and production of antibacterial compounds. Besides, compared with the H group, 2-PBA, which is a derivative of butyrate, was significantly down-regulated in the CM group [54]. Correlation analysis showed that it was positively correlated with butyrate and butyrate-producing bacteria in the rumen and negatively associated with milk SCC. 2-PBA has a protective effect on host mucosal defense during infection, which could increase the number of intestinal *Lactobacillus* and reduce the induction of the pro-inflammatory cytokine IL-23 in macrophage-like cells [55]. The anti-infective effect of 2-PBA may explain its negative correlation with milk SCC during BM.

This study identified the difference of rumen microbiota structure and metabolites among CM, SM and healthy cows. The dysregulation of rumen flora and the significant changes in metabolites related to inflammation were observed during BM. However, the mechanism of the relationships between mastitis and ruminal internal environment still need further investigation. In addition, the composition of the rumen flora and metabolites in dairy cows are also affected by the environment and diet composition. Future research should further investigate the variations of rumen microbes and metabolites during BM with different dietary types, animal bedding types and seasons etc.

## Conclusion

The current study analyzed the differences in rumen microbiome structure and metabolites among the SM, CM and healthy cows. In the rumen of CM cows, the significantly increased bacteria, such as Pseudobutyrivibrio, Gastranaerophilales and *Moraxella*, etc., were found accompanied by the increase of 12-oxo-20-dihydroxy-leukotriene B4 and 10beta-hydroxy-6beta-isobutyrylfuranoeremophilane. The *Ruminiclostridium_9* and *Enterorhabdus* in the rumen of SM cows were found increased with increasing methenamine, 5-HMF and 6-methoxymellein. In contrast, the SCFAs-producing bacteria and intestinal probiotics in the rumen during IMI, including *Prevoterotoella_1*, *Mollicutes_RF39* and *Bifidobacteria*, etc., were significantly reduced with a decrease of 2-PBA, indicating the decline of immunity and anti-infection ability of the dairy cows. In summary, the abundance of microflora and metabolites associated with inflammation were significantly changed in the rumen of mastitis cows. However, the mechanism of the shift in the rumen inner-environment during BM still needs further investigation.

## Supplementary Information

**Additional file 1 Table S1** TMR Ingredient and nutrient component (% of DM). **Table S2** Criteria to judge California mastitis test (CMT) results. **Table S3** Basic information and grouping of experimental cows. **Table S4** Rumen microbial composition and relative abundances of cows with different udder health states (phylum level). **Table S5** Rumen microbial composition and relative abundances of cows with different udder health states (genus level). **Table S6** Differentially abundant KEGG function abundance among health, subclinical and clinical mastitis groups at level 2. **Table S7** Differentially abundant KEGG function abundance among health, subclinical and clinical mastitis groups at level 3. **Table S8** Correlation analysis between the differentially abundant bacteria and parameters of rumen fermentation as well as lactation performance. **Table S9** Significantly different metabolites between rumen fluid samples from healthy and clinical mastitis cows, udder positive ion mode. **Table S10** Significantly different metabolites between rumen fluid samples from healthy and clinical mastitis cows, udder negative ion mode. **Table S11** Significantly different metabolites between rumen fluid samples from healthy and subclinical mastitis cows, udder positive ion mode. **Table S12** Significantly different metabolites between rumen fluid samples from healthy and subclinical mastitis cows, udder negative ion mode. **Table S13** Significantly different metabolites between rumen fluid samples from subclinical and clinical mastitis, udder positive ion mode. **Table S14** Significantly different metabolites between rumen fluid samples from subclinical and clinical mastitis cows, udder negative ion mode. **Table S15** Correlation analysis of differential metabolites and index of lactation and rumen fermentation. **Table S16** Correlation analysis of differential bacteria genus and differential metabolites in rumen samples.

**Additional file 2 Fig. S1** Pan-Species curve of OTU number in the cows with different udder health status. Pan species was the sum of all the species in a sample, which was used to observe the increase in the total number of species as the number of samples increases. H, healthy; SM, subclinical mastitis; CM, clinical mastitis. **Fig. S2** Rarefaction curve of OUT number in the cows with different udder health status. H, healthy; SM, subclinical mastiti; CM, clinical mastitis. **Fig. S3** Rumen microbial community composition analysis. **a** At phylum level. **b** At genus level. H, healthy; SM, subclinical mastitis; CM, clinical mastitis. **Fig.S4** Hierarchical cluster analysis (HCA) of rumen bacteria at genus level. Each row in the figure represents a sample, each column represents a genus, and the color indicates the relative abundance of bacteria measured in the group. Red indicates the high relative abundance, and the green indicates low relative abundance. H, healthy; SM, subclinical mastitis; CM, clinical mastitis. **Fig. S5** Linear discriminant analysis effect size (LEfse) analysis of multilevel species differences in ruminal microbiota. **a** Cladogram; **b** LEfSe Bar graph. H, healthy; SM, subclinical mastitis; CM, clinical mastitis; LDA, linear discriminant analysis. **Fig. S6** The total ion chromatograms (TIC) plot of quality control (QC) samples in **a** positive ion mode and **b** negative ion mode. **Fig. S7** Orthogonal partial least squares discriminant analysis (OPLS-DA) (**a**, **c**, **e**) and response permutation testing (RPT) (**b**, **d**, **f**) of rumen metabolites between H, SM and CM groups in positive ion mode. H, healthy; SM, subclinical mastitis; CM, clinical mastitis. R^2^X and R^2^Y represent the interpretation rate of the built model to the X and Y matrix, R^2^X (cum) and R^2^Y (cum) represent the cumulative interpretation rate; Q^2^ indicates the predictive power of the model. **Fig. S8** Orthogonal partial least squares discriminant analysis (OPLS-DA) (**a**, **c**, **e**) and response permutation testing (RPT) (**b**, **d**, **f**) plots of rumen metabolites between H, SM and CM groups in negative ion mode. H, healthy; SM, subclinical mastitis; CM, clinical mastitis. R^2^X and R^2^Y represent the interpretation rate of the built model to the X and Y matrix, R^2^X (cum) and R^2^Y (cum) represent the cumulative interpretation rate; Q^2^ indicates the predictive power of the model. **Fig. S9** HMDB compound classification (Superclass level) of significantly differential metabolites between **a** CM and H groups, **b** SM and H groups and **c** CM and SM groups. HMDB, Human Metabolome Database; H, healthy; SM, subclinical mastitis; CM, clinical mastitis.

## Data Availability

All data generated or analyzed during this study are included in this published article (and its supplementary information files).

## Abbreviations

IMI: Intramammary infection
BM: Bovine mastitis
CM: Clinical mastitis
SM: Subclinical mastitis
H: Healthy
SCC: Somatic cell counts
CMT: California mastitis test
VFAs: Volatile fatty acids
SCFAs: Short-chain fatty acids
TVFAs: Total volatile fatty acids
Th17: T helper cell 17
LC-MS: Liquid chromatography-mass spectrometry
NH_3_-N: Ammonia nitrogen
RUN: Ruminal fluid urea nitrogen
LA: Lactic acid
OTUs: Operational taxonomic units
PCoA: Principal co-ordinates analysis
HCA: Hierarchical cluster analysis
PICRUSt: Phylogenetic investigation of communities by reconstruction of unobserved states
QC: Quality control
RT: Retention time
HMDB: Human metabolome database
PCA: Principle component analysis
OPLS-DA: Orthogonal partial least squares discriminate analysis
VIP: Variable importance in the projection
A/P: Acetate to propionate ratio
TIC: Total ion chromatogram
2-PBA: 2-Phenylbutyric acid
ROC: Receiver operator characteristic
AUC: Area under curve
LTB4: Leukotriene B4
PMNLs: Polymorphonuclear leukocytes
5-HMF: 5-Hydroxymethyl-2-furancarboxaldehyde
