# Supplementary material for: Rumen microbiome structure and metabolites activity in dairy cows with clinical and subclinical mastitis
Source: J Anim Sci Biotechnol. 2021 Feb 8;12:36. doi: 10.1186/s40104-020-00543-1 (PMC7869221; doi:10.1186/s40104-020-00543-1)
Supplement: Supplementary file 1 — Additional file 1 Table S1 TMR Ingredient and nutrient component (% of DM). Table S2 Criteria to judge California mastitis test (CMT) results. Table S3 Basic information and grouping of experimental cows. Table S4 Rumen microbial composition and relative abundances of cows with different udder health states (phylum level). Table S5 Rumen microbial composition and relative abundances of cows with different udder health states (genus level). Table S6 Differentially abundant KEGG function abundance among health, subclinical and clinical mastitis groups at level 2. Table S7 Differentially abundant KEGG function abundance among health, subclinical and clinical mastitis groups at level 3. Table S8 Correlation analysis between the differentially abundant bacteria and parameters of rumen fermentation as well as lactation performance. Table S9 Significantly different metabolites between rumen fluid samples from healthy and clinical mastitis cows, udder positive ion mode. Table S10 Significantly different metabolites between rumen fluid samples from healthy and clinical mastitis cows, udder negative ion mode. Table S11 Significantly different metabolites between rumen fluid samples from healthy and subclinical mastitis cows, udder positive ion mode. Table S12 Significantly different metabolites between rumen fluid samples from healthy and subclinical mastitis cows, udder negative ion mode. Table S13 Significantly different metabolites between rumen fluid samples from subclinical and clinical mastitis, udder positive ion mode. Table S14 Significantly different metabolites between rumen fluid samples from subclinical and clinical mastitis cows, udder negative ion mode. Table S15 Correlation analysis of differential metabolites and index of lactation and rumen fermentation. Table S16 Correlation analysis of differential bacteria genus and differential metabolites in rumen samples. [file 40104_2020_543_MOESM1_ESM.docx]

**Table S1** TMR Ingredient and nutrient component (% of DM)

| Ingredient, % | Content | Nutrient composition  (%, unless other stated) | Content |
| --- | --- | --- | --- |
| Extruded soybean | 0.45 | CP | 16.9 |
| Steam-flaked corn | 10.5 | NDF | 30.1 |
| Sprayed corn husk | 2.27 | ADF | 17.3 |
| Cottonseed | 7.36 | Ether extract | 4.93 |
| Megalac^a^ | 0.45 | Ca | 0.81 |
| Fatty powder | 0.91 | P | 0.48 |
| Beet granules | 1.36 | NE_L_, Mcal/kg | 1.72 |
| Rapeseed meal | 2.20 |  |  |
| Hulless oat straw | 2.30 |  |  |
| Sunflower meal | 0.64 |  |  |
| Alfalfa hay | 8.18 |  |  |
| Oat grass | 3.18 |  |  |
| Corn silage | 48.3 |  |  |
| 5% Premix^b^ | 4.91 |  |  |
| DDGS^c^ | 4.42 |  |  |
| NaHCO3 | 1.50 |  |  |
| MgO | 0.80 |  |  |
| Sodium diacetate | 0.33 |  |  |

^a^ Megalac, a complex fatty acid calcium (Jianhe Animal Husbandry Co., Ltd., Shanghai, China); ^b^ 5% Premix, including (per kg of DM) 400,000 IU of vitamin A, 320,000 IU of vitamin D_3_, 1200 IU of vitamin E, 1400 mg of Cu, 12,000 mg of Zn, 60,000 mg of Fe, 12,000 mg of Mn, 40 mg of Se, 400 mg of I, 160 mg of Co, 28% of Ca and 5.4% of P;

Abbreviations: DDGS distiller’s dried grain with soluble.

**Table S2** Criteria to judge California mastitis test (CMT) results

| Results | Reactive states | Milk SCC /(×1,000 cells/mL) |
| --- | --- | --- |
| Negative (-) | The mixture is liquid, flowing smoothly and without clots when tilting the test plate. | 0-200 |
| Suspected (±) | The mixture is liquid with a trace of sediment at the bottom, which disappears when shaken. | 200-500 |
| Weakly positive (+) | The bottom of the plate appears a small amount of viscous sediment, which spread in the bottom of the plate and with a certain degree of viscosity when shaken. | 500-800 |
| Positive (++) | The mixture show gelatinous, having certain viscosity and not easy to spread out when turned to the center. | 800-5,000 |
| Strongly positive (+++) | Most or all of the mixture form distinct colloidal deposits, almost completely adherent to the bottom of the plate, gathering in the center and difficult to disperse when shaken. | >5,000 |

Abbreviations: SCC somatic cell counts.

**Table S3** Basic information and grouping of experimental cows

| Sample group | Parity | DIM | Milk yield  kg/d^1^ | Udder clinical symptoms | CMT  Results^3^ | Milk SCC  /(×10^3^ cells / mL)^2^ |
| --- | --- | --- | --- | --- | --- | --- |
| H (*n* = 20) | 2-4 | 149-199 | 42.6 ± 3.44 | No | - | 42.8 ± 5.86 |
| SM (*n* = 20) | 2-4 | 137-202 | 32.7 ± 5.28 | No | + | 803 ± 91.9 |
| CM (*n* = 20) | 2-4 | 156-191 | 28.5 ± 4.53 | Fever, redness and swelling | ++ or +++ | 7,053 ± 173 |

^1, 2^ Milk yield and milk SCC is expressed by mean ± SE; ^3^-, +, ++, +++ represent the diagnosis results of mastitis are negative, weakly positive, positive and strongly positive, respectively.

Abbreviations: H health, SM subclinical mastitis, CM clinical mastitis, DIM days in milk, SCC somatic cell counts, CMT California mastitis test.

**Table S4** Rumen microbial composition and relative abundances of cows with different udder health states (phylum level)

| NO. | Items | H  (*n* = 20) | SM  (*n* = 20) | CM  (*n* = 20) | SEM | *P*-value | FDR |
| --- | --- | --- | --- | --- | --- | --- | --- |
| 1 | Patescibacteria | 0.52^a^ | 0.44^b^ | 0.26^c^ | 0.28 | 0.002 | 0.031 |
| 2 | Verrucomicrobia | 0.001^b^ | 0.00^c^ | 0.002^a^ | 0.002 | 0.003 | 0.037 |
| 3 | Lentisphaerae | 0.01^b^ | 0.01^a^ | 0.004^c^ | 0.01 | 0.003 | 0.037 |
| 4 | Tenericutes | 1.11^a^ | 0.82^c^ | 0.92^b^ | 0.39 | 0.002 | 0.031 |
| 5 | Kiritimatiellaeota | 0.28 | 0.20 | 0.32 | 0.17 | 0.066 | 0.096 |
| 6 | Actinobacteria | 0.85^a^ | 0.55^b^ | 0.39^c^ | 0.50 | 0.003 | 0.034 |
| 7 | WPS-2 | 0.03 | 0.02 | 0.03 | 0.02 | 0.091 | 0.126 |
| 8 | Elusimicrobia | 0.04 | 0.04 | 0.02 | 0.03 | 0.098 | 0.249 |
| 9 | Epsilonbacteraeota | 0.01 | 0.01 | 0.01 | 0.01 | 0.179 | 0.421 |
| 10 | Cyanobacteria | 4.39^b^ | 3.73^c^ | 5.38^a^ | 0.63 | 0.002 | 0.033 |
| 11 | Spirochaetes | 0.55 | 0.51 | 0.41 | 0.36 | 0.494 | 0.910 |
| 12 | Armatimonadetes | 0.004 | 0.003 | 0.01 | 0.01 | 0.574 | 0.910 |
| 13 | Bacteria | 0.20 | 0.18 | 0.19 | 0.18 | 0.583 | 0.913 |
| 14 | Fusobacteria | 0.002 | 0.004 | 0.01 | 0.02 | 0.696 | 0.914 |
| 15 | Synergistetes | 0.04 | 0.04 | 0.03 | 0.03 | 0.699 | 0.914 |
| 16 | Bacteroidetes | 38.1^b^ | 36.4^c^ | 40.3^a^ | 6.94 | 0.002 | 0.035 |
| 17 | Fibrobacteres | 0.16 | 0.18 | 0.13 | 0.14 | 0.787 | 0.920 |
| 18 | Firmicutes | 43.4^c^ | 48.3^b^ | 54.0^a^ | 11.3 | 0.001 | 0.030 |
| 19 | Chloroflexi | 0.01 | 0.01 | 0.01 | 0.01 | 0.827 | 0.935 |
| 20 | Proteobacteria | 10.3^a^ | 8.60^b^ | 7.45^c^ | 10.8 | 0.002 | 0.032 |
| 21 | Deinococcus-Thermus | 0.001 | 0.001 | 0.002 | 0.003 | 0.998 | 0.100 |

Abbreviations: H healthy, SM subclinical mastitis, CM clinical mastitis, SEM standard error of the mean, FDR false discovery rate, ^a, b, c^ values within a row, different letters denote differences between treatment combinations (FDR-adjusted *P* < 0.05).

**Table S5** Rumen microbial composition and relative abundances of cows with different udder health states (genus level)

| NO. | Items | H  (*n* = 20) | SM  (*n* = 20) | CM  (*n* = 20) | SEM | *P*-value | FDR |
| --- | --- | --- | --- | --- | --- | --- | --- |
| 1 | *Prevotella_1* | 25.1^a^ | 21.2^b^ | 17.3^c^ | 5.35 | 0.003 | 0.045 |
| 2 | *Succiniclasticum* | 9.66 | 10.2 | 9.56 | 6.182 | 0.967 | 0.990 |
| 3 | *Muribaculaceae* | 7.92 | 8.26 | 9.44 | 5.163 | 0.450 | 0.803 |
| 4 | *Prevotella_7* | 3.88 | 5.86 | 4.72 | 6.532 | 0.753 | 0.911 |
| 5 | *Ruminococcaceae_NK4A214_group* | 3.26 | 2.93 | 2.91 | 1.538 | 0.718 | 0.903 |
| 6 | *Succinivibrionaceae_UCG-001* | 2.69 | 4.53 | 4.43 | 7.034 | 0.299 | 0.751 |
| 7 | *Prevotellaceae* | 2.39 | 2.60 | 2.28 | 1.156 | 0.540 | 0.881 |
| 8 | *Ruminococcaceae_UCG-014* | 2.13 | 1.73 | 1.36 | 0.872 | 0.064 | 0.660 |
| 9 | *Rikenellaceae_RC9_gut_group* | 1.98 | 1.84 | 2.07 | 0.881 | 0.845 | 0.946 |
| 10 | *F082* | 1.86 | 1.71 | 1.90 | 0.843 | 0.666 | 0.890 |
| 11 | *Christensenellaceae_R-7_group* | 1.85 | 1.56 | 1.76 | 0.857 | 0.614 | 0.880 |
| 12 | *Prevotellaceae_UCG-001* | 1.69 | 1.82 | 1.96 | 0.823 | 0.708 | 0.901 |
| 13 | *Lachnospiraceae_NK3A20_group* | 1.62 | 1.73 | 1.61 | 0.749 | 0.813 | 0.940 |
| 14 | *Prevotellaceae_UCG-003* | 1.54 | 1.86 | 1.56 | 0.839 | 0.333 | 0.755 |
| 15 | *Ruminococcus_2* | 1.40 | 0.82 | 0.99 | 1.041 | 0.499 | 0.852 |
| 16 | *[Eubacterium]_ruminantium_group* | 1.00 | 0.92 | 1.05 | 0.477 | 0.554 | 0.880 |
| 17 | *Butyrivibrio_2* | 0.96 | 0.87 | 1.47 | 0.880 | 0.548 | 0.880 |
| 18 | *Mollicutes_RF39* | 0.91^a^ | 0.66^c^ | 0.70^b^ | 0.354 | 0.003 | 0.047 |
| 19 | *Lachnospiraceae* | 0.80 | 0.75 | 0.94 | 0.526 | 0.407 | 0.771 |
| 20 | *Ruminococcus_1* | 0.79 | 0.72 | 0.85 | 0.473 | 0.102 | 0.671 |
| 21 | *Shuttleworthia* | 0.75 | 1.04 | 0.66 | 0.729 | 0.562 | 0.880 |
| 22 | *Candidatus_Saccharimonas* | 0.71 | 0.59 | 0.65 | 0.374 | 0.313 | 0.752 |
| 23 | *Absconditabacteriales_SR1* | 0.67 | 0.58 | 0.62 | 0.377 | 0.596 | 0.880 |
| 24 | *Saccharofermentans* | 0.65 | 0.61 | 0.89 | 0.335 | 0.121 | 0.674 |
| 25 | *Selenomonas_1* | 0.64 | 0.53 | 1.04 | 0.772 | 0.199 | 0.690 |
| 26 | *Clostridiales* | 0.58 | 0.48 | 0.58 | 0.334 | 0.458 | 0.801 |
| 27 | *Prevotellaceae_YAB2003_group* | 0.53 | 0.51 | 0.34 | 0.500 | 0.247 | 0.710 |
| 28 | *Treponema_2* | 0.53 | 0.49 | 0.39 | 0.353 | 0.515 | 0.870 |
| 29 | *Acetitomaculum* | 0.52 | 0.52 | 0.50 | 0.295 | 0.770 | 0.910 |
| 30 | *Pseudobutyrivibrio* | 0.51^b^ | 0.39^c^ | 0.66^a^ | 0.313 | 0.003 | 0.047 |
| 31 | *Moryella* | 0.50 | 0.45 | 0.49 | 0.209 | 0.622 | 0.881 |
| 32 | *Lachnospiraceae_NK4A136_group* | 0.49 | 0.42 | 0.54 | 0.274 | 0.300 | 0.753 |
| 33 | *Bacteroidales_RF16_group* | 0.48 | 0.54 | 0.91 | 0.719 | 0.837 | 0.940 |
| 34 | *Bifidobacterium* | 0.49^a^ | 0.24^b^ | 0.12^c^ | 0.380 | 0.002 | 0.035 |
| 35 | *Lachnospiraceae* | 0.48^a^ | 0.33^b^ | 0.23^c^ | 0.274 | 0.002 | 0.039 |
| 36 | *Schwartzia* | 0.46 | 0.47 | 0.88 | 0.500 | 0.054 | 0.642 |
| 37 | *Ruminococcaceae_UCG-005* | 0.45 | 0.39 | 0.67 | 0.333 | 0.052 | 0.641 |
| 38 | *Lachnospiraceae_AC2044_group* | 0.45 | 0.45 | 0.59 | 0.232 | 0.394 | 0.760 |
| 39 | *[Ruminococcus]_gauvreauii_group* | 0.45 | 0.57 | 0.46 | 0.349 | 0.931 | 0.971 |
| 40 | *Gastranaerophilales* | 0.21^c^ | 0.33^b^ | 0.44^a^ | 0.270 | 0.002 | 0.037 |
| 41 | *[Eubacterium]_coprostanoligenes_group* | 0.41 | 0.40 | 0.56 | 0.233 | 0.490 | 0.845 |
| 42 | *Oribacterium* | 0.37 | 0.38 | 0.39 | 0.261 | 0.693 | 0.900 |

Abbreviations: H healthy, SM subclinical mastitis, CM clinical mastitis, SEM standard error of the mean, FDR false discovery rate, ^a, b, c^ values within a row, different letters denote differences between treatment combinations (FDR-adjusted *P* < 0.05).

**Table S5** Continued

| NO. | Items | H  (*n* = 20) | SM  (*n* = 20) | CM  (*n* = 20) | SEM | *P*-value | FDR |
| --- | --- | --- | --- | --- | --- | --- | --- |
| 43 | *Bacteroidales* | 0.36 | 0.34 | 0.25 | 0.163 | 0.113 | 0.670 |
| 44 | *Veillonellaceae* | 0.36 | 0.38 | 0.49 | 0.383 | 0.990 | 1.000 |
| 45 | *Lachnoclostridium_1* | 0.33 | 0.35 | 0.34 | 0.169 | 0.685 | 0.903 |
| 46 | *Lachnospiraceae_XPB1014_group* | 0.33 | 0.27 | 0.50 | 0.217 | 0.212 | 0.710 |
| 47 | *Prevotellaceae_UCG-004* | 0.33 | 0.32 | 0.28 | 0.189 | 0.630 | 0.880 |
| 48 | *Lachnospira* | 0.32 | 0.30 | 0.22 | 0.254 | 0.251 | 0.712 |
| 49 | *WCHB1-41* | 0.28 | 0.20 | 0.32 | 0.166 | 0.066 | 0.660 |
| 50 | *p-251-o5* | 0.28 | 0.25 | 0.34 | 0.343 | 0.542 | 0.885 |
| 51 | *Ruminococcaceae_UCG-002* | 0.25 | 0.22 | 0.31 | 0.156 | 0.369 | 0.750 |
| 52 | *Family_XIII_AD3011_group* | 0.24 | 0.18 | 0.28 | 0.129 | 0.148 | 0.673 |
| 53 | *Anaerovibrio* | 0.23 | 0.20 | 0.51 | 0.367 | 0.132 | 0.670 |
| 54 | *Veillonellaceae_UCG-001* | 0.23 | 0.19 | 0.44 | 0.233 | 0.084 | 0.670 |
| 55 | *Ruminococcaceae_UCG-001* | 0.23 | 0.16 | 0.13 | 0.169 | 0.164 | 0.670 |
| 56 | *Erysipelotrichaceae_UCG-004* | 0.22 | 0.25 | 0.28 | 0.200 | 0.842 | 0.941 |
| 57 | *Prevotellaceae_NK3B31_group* | 0.22 | 0.17 | 0.26 | 0.132 | 0.219 | 0.710 |
| 58 | *Succinivibrionaceae_UCG-002* | 0.20 | 0.25 | 0.28 | 0.497 | 0.877 | 0.951 |
| 59 | *Ruminococcaceae_UCG-010* | 0.20 | 0.18 | 0.29 | 0.143 | 0.399 | 0.760 |
| 60 | *Bacteria* | 0.19 | 0.18 | 0.19 | 0.176 | 0.583 | 0.880 |
| 61 | *CAG-352* | 0.19 | 0.11 | 0.12 | 0.128 | 0.021 | 0.357 |
| 62 | *Desulfovibrio* | 0.19 | 0.17 | 0.16 | 0.095 | 0.271 | 0.741 |
| 63 | *[Eubacterium]_nodatum_group* | 0.18 | 0.17 | 0.21 | 0.225 | 0.859 | 0.952 |
| 64 | *Anaerovorax* | 0.17 | 0.14 | 0.21 | 0.086 | 0.083 | 0.673 |
| 65 | *Pseudoscardovia* | 0.17 | 0.04 | 0.05 | 0.178 | 0.062 | 0.660 |
| 66 | *Fibrobacter* | 0.16 | 0.18 | 0.13 | 0.139 | 0.787 | 0.931 |
| 67 | *Lachnobacterium* | 0.16^a^ | 0.09^b^ | 0.16^a^ | 0.091 | 0.002 | 0.040 |
| 68 | *Anaeroplasma* | 0.15 | 0.12 | 0.16 | 0.090 | 0.237 | 0.710 |
| 69 | *Syntrophococcus* | 0.14 | 0.30 | 0.14 | 0.250 | 0.917 | 0.973 |
| 70 | *Prevotellaceae_Ga6A1_group* | 0.14 | 0.11 | 0.11 | 0.119 | 0.277 | 0.755 |
| 71 | *Ruminococcaceae* | 0.14 | 0.13 | 0.16 | 0.073 | 0.310 | 0.750 |
| 72 | *Veillonellaceae* | 0.14 | 0.10 | 0.26 | 0.238 | 0.491 | 0.843 |
| 73 | *probable_genus_10* | 0.13 | 0.13 | 0.23 | 0.118 | 0.251 | 0.710 |
| 74 | *Ruminiclostridium_9* | 0.13^b^ | 0.23^a^ | 0.10^b^ | 0.177 | 0.003 | 0.042 |
| 75 | *Selenomonas* | 0.13 | 0.06 | 0.23 | 0.193 | 0.205 | 0.711 |
| 76 | *Lachnospiraceae_UCG-008* | 0.13 | 0.12 | 0.18 | 0.077 | 0.066 | 0.660 |
| 77 | *[Eubacterium]_ventriosum_group* | 0.12 | 0.09 | 0.12 | 0.096 | 0.238 | 0.714 |
| 78 | *Erysipelotrichaceae* | 0.12 | 0.11 | 0.14 | 0.076 | 0.280 | 0.750 |
| 79 | *Ruminococcaceae* | 0.12 | 0.09 | 0.16 | 0.076 | 0.100 | 0.670 |
| 80 | *Defluviitaleaceae_UCG-011* | 0.11 | 0.10 | 0.11 | 0.053 | 0.651 | 0.891 |
| 81 | *Bacteroidales_BS11_gut_group* | 0.10 | 0.08 | 0.15 | 0.111 | 0.127 | 0.678 |
| 82 | *Olsenella* | 0.10 | 0.14 | 0.08 | 0.098 | 0.308 | 0.750 |
| 83 | *Dialister* | 0.10 | 0.22 | 0.22 | 0.345 | 0.700 | 0.902 |

Abbreviations: H healthy, SM subclinical mastitis, CM clinical mastitis, SEM standard error of the mean, FDR false discovery rate, ^a, b, c^ values within a row, different letters denote differences between treatment combinations (FDR-adjusted *P* < 0.05).

**Table S5** Continued

| NO. | Items | H  (*n* = 20) | SM  (*n* = 20) | CM  (*n* = 20) | SEM | *P*-value | FDR |
| --- | --- | --- | --- | --- | --- | --- | --- |
| 84 | *[Eubacterium]_hallii_group* | 0.09 | 0.11 | 0.08 | 0.047 | 0.213 | 0.711 |
| 85 | *FD2005* | 0.09 | 0.07 | 0.10 | 0.066 | 0.047 | 0.621 |
| 86 | *Lachnospiraceae_ND3007_group* | 0.09 | 0.10 | 0.16 | 0.117 | 0.128 | 0.670 |
| 87 | *Bacteroidales* | 0.08^a^ | 0.05^b^ | 0.05^b^ | 0.061 | 0.001 | 0.031 |
| 88 | *Marvinbryantia* | 0.08 | 0.11 | 0.09 | 0.073 | 0.937 | 0.978 |
| 89 | *Prevotellaceae* | 0.07 | 0.10 | 0.14 | 0.092 | 0.066 | 0.565 |
| 90 | *Bradymonadales* | 0.07 | 0.06 | 0.09 | 0.063 | 0.559 | 0.880 |
| 91 | *Succinivibrio* | 0.07 | 0.12 | 0.06 | 0.101 | 0.733 | 0.900 |
| 92 | *Papillibacter* | 0.07 | 0.05 | 0.08 | 0.043 | 0.106 | 0.671 |
| 93 | *U29-B03* | 0.07 | 0.06 | 0.07 | 0.040 | 0.873 | 0.950 |
| 94 | *[Eubacterium]_cellulosolvens_group* | 0.07 | 0.08 | 0.07 | 0.073 | 0.484 | 0.840 |
| 95 | *[Anaerorhabdus]_furcosa_group* | 0.07 | 0.05 | 0.08 | 0.037 | 0.146 | 0.671 |
| 96 | *Alloprevotella* | 0.06 | 0.06 | 0.05 | 0.037 | 0.766 | 0.914 |
| 97 | *Roseburia* | 0.05 | 0.04 | 0.06 | 0.040 | 0.394 | 0.760 |
| 98 | *Rhodospirillales* | 0.05 | 0.07 | 0.04 | 0.080 | 0.182 | 0.670 |
| 99 | *Mogibacterium* | 0.05 | 0.04 | 0.05 | 0.032 | 0.196 | 0.690 |
| 100 | *Ruminococcaceae_UCG-004* | 0.05 | 0.05 | 0.07 | 0.043 | 0.786 | 0.933 |
| 101 | *Coprococcus_2* | 0.05 | 0.02 | 0.04 | 0.039 | 0.166 | 0.672 |
| 102 | *Asteroleplasma* | 0.05 | 0.05 | 0.04 | 0.046 | 0.112 | 0.670 |
| 103 | *Bacteroidia* | 0.05 | 0.05 | 0.04 | 0.033 | 0.565 | 0.880 |
| 104 | *Erysipelotrichaceae* | 0.04 | 0.05 | 0.04 | 0.033 | 0.880 | 0.950 |
| 105 | *Lachnospiraceae_UCG-006* | 0.05^b^ | 0.04^c^ | 0.06^a^ | 0.022 | 0.003 | 0.047 |
| 106 | *Blautia* | 0.04 | 0.07 | 0.07 | 0.044 | 0.212 | 0.710 |
| 107 | *[Eubacterium]_xylanophilum_group* | 0.04 | 0.02 | 0.05 | 0.031 | 0.079 | 0.674 |
| 108 | *Elusimicrobium* | 0.03 | 0.03 | 0.02 | 0.022 | 0.044 | 0.620 |
| 109 | *SP3-e08* | 0.03 | 0.05 | 0.02 | 0.042 | 0.099 | 0.671 |
| 110 | *Tyzzerella_3* | 0.03 | 0.02 | 0.03 | 0.022 | 0.109 | 0.671 |
| 111 | *Bacteroidales_UCG-001* | 0.03^b^ | 0.05^a^ | 0.03^b^ | 0.025 | 0.002 | 0.038 |
| 112 | *Mitochondria* | 0.03 | 0.02 | 0.04 | 0.026 | 0.179 | 0.670 |
| 113 | *Mycoplasma* | 0.03 | 0.03 | 0.03 | 0.038 | 0.697 | 0.907 |
| 114 | *Sharpea* | 0.03 | 0.03 | 0.01 | 0.045 | 0.134 | 0.670 |
| 115 | *WPS-2* | 0.03 | 0.02 | 0.03 | 0.024 | 0.091 | 0.674 |
| 116 | *Ruminiclostridium* | 0.03 | 0.03 | 0.04 | 0.035 | 0.540 | 0.880 |
| 117 | *Ruminiclostridium_6* | 0.03 | 0.03 | 0.03 | 0.029 | 0.259 | 0.730 |
| 118 | *[Eubacterium]_brachy_group* | 0.03 | 0.05 | 0.05 | 0.034 | 0.351 | 0.752 |
| 119 | *Megasphaera* | 0.03 | 0.03 | 0.03 | 0.044 | 0.606 | 0.880 |
| 120 | *p-2534-18B5_gut_group* | 0.03 | 0.09 | 0.01 | 0.105 | 0.116 | 0.671 |
| 121 | *Allisonella* | 0.03 | 0.05 | 0.03 | 0.067 | 0.243 | 0.710 |
| 122 | *Howardella* | 0.03 | 0.03 | 0.03 | 0.017 | 0.876 | 0.950 |
| 123 | *Clostridiales_vadinBB60_group* | 0.03 | 0.02 | 0.04 | 0.034 | 0.328 | 0.075 |
| 124 | *Prevotella_9* | 0.03 | 0.02 | 0.02 | 0.017 | 0.241 | 0.071 |

H, healthy; SM, subclinical mastitis; CM, clinical mastitis; SEM, standard error of the mean, FDR false discovery rate,

^a, b, c^ values within a row, different letters denote differences between treatment combinations (FDR-adjusted *P* < 0.05).

**Table S5** Continued

| NO. | Items | H  (*n* = 20) | SM  (*n* = 20) | CM  (*n* = 20) | SEM | *P*-value | FDR |
| --- | --- | --- | --- | --- | --- | --- | --- |
| 125 | *Lachnospiraceae_FCS020_group* | 0.03 | 0.03 | 0.04 | 0.026 | 0.955 | 0.990 |
| 126 | *Lactobacillus* | 0.03 | 0.04 | 0.02 | 0.038 | 0.449 | 0.803 |
| 127 | *Desulfobulbus* | 0.02 | 0.02 | 0.04 | 0.021 | 0.085 | 0.670 |
| 128 | *Lachnospiraceae_UCG-004* | 0.02 | 0.03 | 0.01 | 0.025 | 0.354 | 0.750 |
| 129 | *Weissella* | 0.02 | 0.04 | 0.02 | 0.039 | 0.194 | 0.691 |
| 130 | *Acidaminococcus* | 0.02 | 0.02 | 0.04 | 0.034 | 0.639 | 0.880 |
| 131 | *DNF00809* | 0.02 | 0.03 | 0.02 | 0.018 | 0.473 | 0.820 |
| 132 | *Erysipelotrichaceae_UCG-009* | 0.02 | 0.05 | 0.03 | 0.048 | 0.619 | 0.880 |
| 133 | *Pyramidobacter* | 0.02 | 0.03 | 0.02 | 0.021 | 0.614 | 0.880 |
| 134 | *Coprococcus_1* | 0.02 | 0.03 | 0.03 | 0.018 | 0.319 | 0.750 |
| 135 | *Eggerthellaceae* | 0.02 | 0.02 | 0.02 | 0.015 | 0.822 | 0.940 |
| 136 | *Christensenellaceae* | 0.02 | 0.02 | 0.02 | 0.011 | 0.970 | 0.992 |
| 137 | *Ruminiclostridium_1* | 0.02 | 0.02 | 0.03 | 0.027 | 0.238 | 0.717 |
| 138 | *Lachnospiraceae_UCG-002* | 0.02 | 0.02 | 0.03 | 0.012 | 0.186 | 0.680 |
| 139 | *Family_XIII_UCG-001* | 0.02 | 0.01 | 0.02 | 0.011 | 0.391 | 0.761 |
| 140 | *Ruminococcaceae_V9D2013_group* | 0.02 | 0.01 | 0.01 | 0.020 | 0.457 | 0.801 |
| 141 | *Atopobium* | 0.02 | 0.02 | 0.02 | 0.017 | 0.980 | 1.000 |
| 142 | *Mailhella* | 0.02 | 0.01 | 0.01 | 0.013 | 0.524 | 0.880 |
| 143 | *Lachnoclostridium_12* | 0.02 | 0.02 | 0.01 | 0.016 | 0.170 | 0.670 |
| 144 | *Lachnospiraceae_UCG-010* | 0.02 | 0.01 | 0.02 | 0.009 | 0.160 | 0.670 |
| 145 | *Rikenellaceae* | 0.01 | 0.02 | 0.02 | 0.011 | 0.948 | 0.983 |
| 146 | *Sphaerochaeta* | 0.01 | 0.01 | 0.01 | 0.014 | 0.734 | 0.904 |
| 147 | *Ruminococcaceae_UCG-013* | 0.01 | 0.02 | 0.03 | 0.021 | 0.386 | 0.760 |
| 148 | *Family_XIII* | 0.01 | 0.01 | 0.01 | 0.007 | 0.538 | 0.880 |
| 149 | *Candidatus_Soleaferrea* | 0.01 | 0.01 | 0.02 | 0.020 | 0.199 | 0.691 |
| 150 | *Ruminococcaceae_UCG-009* | 0.01 | 0.01 | 0.01 | 0.008 | 0.295 | 0.750 |
| 151 | *Lachnoclostridium_10* | 0.01 | 0.01 | 0.03 | 0.022 | 0.378 | 0.753 |
| 152 | *Corynebacterium_1* | 0.01 | 0.02 | 0.02 | 0.028 | 0.659 | 0.894 |
| 153 | *Peptococcaceae* | 0.01 | 0.01 | 0.01 | 0.007 | 0.175 | 0.670 |
| 154 | *Lachnospiraceae_NC2004_group* | 0.01 | 0.01 | 0.01 | 0.015 | 0.877 | 0.952 |
| 155 | *Synergistes* | 0.01 | 0.01 | 0.01 | 0.008 | 0.432 | 0.800 |
| 156 | *Fretibacterium* | 0.01 | 0.01 | 0.01 | 0.005 | 0.235 | 0.711 |
| 157 | *Erysipelotrichaceae_UCG-002* | 0.01 | 0.07 | 0.01 | 0.084 | 0.224 | 0.711 |
| 158 | Others | 4.63 | 6.52 | 6.42 | 0.012 | 0.031 | 0.725 |

Abbreviations: H healthy, SM subclinical mastitis, CM clinical mastitis, SEM standard error of the mean, FDR false discovery rate, ^a, b, c^ values within a row, different letters denote differences between treatment combinations (FDR-adjusted *P* < 0.05).

Table S6 Differentially abundant KEGG function abundance among health, subclinical and clinical mastitis groups at level 2

| Class level | H  (*n* = 20) | SM  (*n* = 20) | CM  (*n* = 20) | SEM | *P*-value | FDR |
| --- | --- | --- | --- | --- | --- | --- |
| Enzyme families | 618,989^c^ | 1,001,389^a^ | 710,558^b^ | 34655 | <0.001 | 0.001 |
| Genetic information processing | 784,325^b^ | 1,357,859^a^ | 573,133^c^ | 43319 | <0.001 | 0.002 |
| Translation | 851,960^b^ | 1,678,235^a^ | 879,189^b^ | 38325 | <0.001 | 0.003 |
| Carbohydrate metabolism | 1,380,913^b^ | 1,792,293^a^ | 1,423,277^b^ | 18475 | <0.001 | 0.002 |
| Immune system | 1,154,935^a^ | 825,804^b^ | 616,304^c^ | 4261 | <0.001 | 0.002 |
| Nervous system | 1,199,815^a^ | 796,936^b^ | 610,017^c^ | 2446 | <0.001 | 0.002 |
| Transport and catabolism | 1,183,231^a^ | 520,239^b^ | 414,404^c^ | 3885 | 0.005 | 0.002 |
| Endocrine system | 1,118,717^a^ | 756,415^b^ | 623,746^c^ | 7680 | <0.001 | 0.002 |
| Biosynthesis of other secondary metabolites | 1,212,516^a^ | 653,559^c^ | 811,405^b^ | 19241 | <0.001 | 0.002 |
| Xenobiotics biodegradation and metabolism | 1,320,732^a^ | 259,823^b^ | 339,156^b^ | 9407 | <0.001 | 0.001 |
| Metabolism of other amino acids | 1,372,397^a^ | 858,704^b^ | 739,718^b^ | 17710 | <0.001 | 0.002 |
| Metabolism of terpenoids and polyketides | 1,396,333^a^ | 930,815^b^ | 748,360^c^ | 13362 | <0.001 | 0.002 |
| Lipid metabolism | 1,428,206^a^ | 806,528^b^ | 727,840^c^ | 25228 | <0.001 | 0.014 |
| Nucleotide metabolism | 1,432,389^a^ | 988,817^b^ | 937,148^b^ | 5648 | <0.001 | 0.001 |
| Metabolism of cofactors and vitamins | 1,549,706^a^ | 1,003,593^b^ | 857,129^c^ | 6199 | <0.001 | 0.004 |
| Amino acid metabolism | 1,588,164^a^ | 1,013,456^b^ | 1,036,542^b^ | 9721 | <0.001 | 0.008 |
| Glycan biosynthesis and metabolism | 893,595^b^ | 891,123^b^ | 1,015,934^a^ | 25628 | 0.001 | 0.007 |
| Metabloic diseases | 903,595^b^ | 891,123^c^ | 1,215,934^a^ | 25628 | 0.003 | 0.001 |
| Replication and repair | 883,125^c^ | 948,730^b^ | 1,473,991^a^ | 4506 | <0.001 | 0.087 |
| Membrane transport | 993,595^b^ | 1,091,123^b^ | 1,515,934^a^ | 25628 | 0.001 | 0.403 |
| Immune system Diseases | 662,854^c^ | 816,795^b^ | 1,549,236^a^ | 74521 | 0.009 | 0.001 |
| Carbohydrate metabolism | 853,966^c^ | 1,143,423^b^ | 1,576,739^a^ | 13152 | <0.001 | 0.002 |
| Amino acid metabolism | 903,595^b^ | 891,123^b^ | 1,595,934^a^ | 25628 | 0.003 | 0.002 |
| Infectious diseases | 924,035^c^ | 1,319,503^b^ | 1,604,664^a^ | 5862 | <0.001 | 0.014 |
| Energy metabolism | 1,209,125^b^ | 1,316,932^b^ | 1,821,398^a^ | 5187 | <0.001 | 0.001 |

Abbreviations: H healthy, SM subclinical mastitis, CM clinical mastitis, SEM standard error of the mean, FDR false discovery rate, ^a, b, c^ values within a row, different letters denote differences between treatment combinations (FDR-adjusted *P* < 0.05).

**Table S7** Differentially abundant KEGG function abundance among health, subclinical and clinical mastitis groups at level 3

| Subclass level | H  (*n* = 20) | SM  (*n* = 20) | CM  (*n* = 20) | SEM | *P*-value | FDR |
| --- | --- | --- | --- | --- | --- | --- |
| Protein kinases | 744,997^c^ | 1,280,455^a^ | 855,014^b^ | 8689 | <0.001 | 0.003 |
| Peptidases | 984,938^b^ | 1,277,628^a^ | 895,202^c^ | 2460 | 0.712 | 1.000 |
| Translation proteins | 822,907^c^ | 1,302,925^a^ | 938,639^b^ | 15847 | 0.075 | 0.454 |
| Peroxisome | 854,138^a^ | 470,113^c^ | 595,258^b^ | 1454 | <0.001 | 0.001 |
| Isoflavonoid biosynthesis | 831,201^a^ | 503,746^b^ | 402,863^c^ | 1372 | 0.001 | 0.001 |
| Flavone and flavonol biosynthesis | 1,002,753^a^ | 762,015^b^ | 542,211^c^ | 1345 | <0.001 | 0.002 |
| Naphthalene degradation | 1,006,602^a^ | 785,542^b^ | 637,583^c^ | 1358 | 0.088 | 0.11 |
| Benzoate degradation | 1,048,933^a^ | 839,515^b^ | 754,410^c^ | 2719 | <0.001 | 0.002 |
| Selenocompound metabolism | 1,067,168^a^ | 734,332^b^ | 671,043^c^ | 1347 | 0.072 | 0.073 |
| Cyanoamino acid metabolism | 1,149,029^a^ | 820,151^b^ | 746,581^c^ | 2570 | 0.004 | 0.060 |
| Terpenoid backbone biosynthesis | 1,292,095^a^ | 836,737^b^ | 702,162^c^ | 2507 | 0.388 | 1.000 |
| Fatty acid biosynthesis | 1,305,600^a^ | 962,825^b^ | 935,222^b^ | 1243 | 0.154 | 1.000 |
| Glycerophosphlipid metabolism | 1,311,298^a^ | 923,202^b^ | 826,511^c^ | 2927 | 0.038 | 0.043 |
| Lipid biosynthesis proteins | 1,329,156^a^ | 836,670^b^ | 840,267^b^ | 2180 | 0.016 | 0.087 |
| Purine metabolism | 1,220,466^a^ | 994,163^c^ | 1,052,252^b^ | 3072 | <0.001 | 0.003 |
| Pyrimidine metabolism | 1,103,426^a^ | 894,006^b^ | 1,119,226^a^ | 2603 | <0.001 | 0.003 |
| Thiamine metabolism | 1,284,203^a^ | 1,027,779^b^ | 887,362^c^ | 3248 | 0.064 | 0.654 |
| Porphyrin and chlorophyll metabolism | 1,440,241^a^ | 1,052,258^b^ | 1,061,863^b^ | 1578 | <0.001 | 0.001 |
| One carbon pool by folate | 1,436,460^a^ | 972,369^a^ | 1,109,453^b^ | 2894 | <0.001 | 0.002 |
| Valine, leucine and isoleucine biosynthesis | 1,491,167^a^ | 1,103,308^c^ | 1,258,683^b^ | 2713 | 0.003 | 0.008 |
| Lysine biosynthesis | 1,532,327^a^ | 1,264,503^c^ | 1,337,275^b^ | 3554 | 0.004 | 0.029 |
| Glycine, serine and threonine metabolism | 961,671^c^ | 1,047,725^b^ | 1,652,168^a^ | 1330 | 0.009 | 0.087 |
| Glycosyltransferases | 1,250,790^b^ | 1,102,202^c^ | 1,451,878^a^ | 1333 | <0.001 | 0.003 |
| Lipopolysaccharide biosynthesis proteins | 1,131,195^c^ | 1,208,645^b^ | 1,490,310^a^ | 1374 | <0.001 | 0.003 |
| Lipopolysaccharide biosynthsis | 1,026,548^b^ | 1,122,636^b^ | 1,462,769^a^ | 2864 | <0.001 | 0.002 |
| Peptidoglycan biosynthesis | 1,064,812^c^ | 1,375,395^b^ | 1,515,906^a^ | 1574 | <0.001 | 0.003 |
| Type II diabetes mellitus | 1,227,302^b^ | 1,122,015^c^ | 1,528,415^a^ | 2713 | 0.056 | 0.403 |
| Primary immunodeficiency | 1,323,463^b^ | 1,219,632^b^ | 1,574,924^a^ | 3745 | <0.001 | 0.004 |
| Bacterial invasion of epithelial cells | 972,828^c^ | 1,131,096^b^ | 1,610,362^a^ | 2261 | <0.001 | 0.002 |
| Staphylococcus aureus infection | 1,135,876^c^ | 1,343,628^b^ | 1,611,026^a^ | 2469 | <0.001 | 0.001 |
| Tuberculosis | 1,376,860^c^ | 1,461,825^b^ | 1,717,089^a^ | 1840 | <0.001 | 0.003 |
| Carbon fixation pathways in prokaryotes | 1,552,610^b^ | 1,446,331^c^ | 1,832,888^a^ | 2332 | 0.001 | 0.006 |
| Methane metabolism | 1,408,377^b^ | 1,331,438^c^ | 1,842,948^a^ | 1418 | <0.001 | 0.003 |

Abbreviations: H healthy, SM subclinical mastitis, CM clinical mastitis, SEM standard error of the mean, FDR false discovery rate, ^a, b, c^ values within a row, different letters denote differences between treatment combinations (FDR-adjusted *P* < 0.05).

**Table S8** Correlation analysis between the differentially abundant bacteria and parameters of rumen fermentation as well as lactation performance

| Items | Items | R | *P*-value | FDR |
| --- | --- | --- | --- | --- |
| *Prevotella_1* | Acetate | 0.384 | 0.002 | 0.047 |
| *Mollicutes_RF39* | Acetate | 0.119 | 0.365 | 0.750 |
| *Lachnospiraceae* | Acetate | 0.291 | 0.024 | 0.583 |
| *Bifidobacterium* | Acetate | 0.254 | 0.050 | 0.687 |
| *Izimaplasmatales* | Acetate | 0.258 | 0.047 | 0.615 |
| *Bacteroidales* | Acetate | 0.069 | 0.599 | 0.876 |
| *Ruminiclostridium_9* | Acetate | 0.122 | 0.354 | 0.756 |
| *Ornithinibacillus* | Acetate | 0.181 | 0.166 | 0.625 |
| *CAG-352* | Acetate | 0.040 | 0.763 | 0.902 |
| *Bacteroidales_UCG-001* | Acetate | -0.035 | 0.791 | 0.911 |
| *Christensenellaceae* | Acetate | -0.099 | 0.451 | 0.865 |
| *Enterorhabdus* | Acetate | -0.188 | 0.151 | 0.666 |
| *Lachnospiraceae_UCG-006* | Acetate | -0.101 | 0.443 | 0.727 |
| *Neisseriaceae* | Acetate | -0.178 | 0.175 | 0.668 |
| *Pasteurellaceae* | Acetate | -0.116 | 0.377 | 0.742 |
| *Pseudobutyrivibrio* | Acetate | -0.163 | 0.213 | 0.288 |
| *Lachnobacterium* | Acetate | -0.064 | 0.630 | 0.877 |
| *Ralstonia* | Acetate | -0.248 | 0.056 | 0.648 |
| *Moraxella* | Acetate | -0.396 | 0.002 | 0.045 |
| *Syntrophomonas* | Acetate | -0.167 | 0.201 | 0.702 |
| *Herbaspirillum* | Acetate | -0.290 | 0.025 | 0.557 |
| *Gastranaerophilales* | Acetate | -0.396 | 0.002 | 0.044 |
| *Prevotella_1* | Propionate | 0.255 | 0.050 | 0.624 |
| *Mollicutes_RF39* | Propionate | 0.142 | 0.278 | 0.710 |
| *Lachnospiraceae* | Propionate | 0.214 | 0.101 | 0.662 |
| *Bifidobacterium* | Propionate | 0.169 | 0.196 | 0.663 |
| *Izimaplasmatales* | Propionate | 0.078 | 0.553 | 0.875 |
| *Bacteroidales* | Propionate | -0.048 | 0.715 | 0.901 |
| *Ruminiclostridium_9* | Propionate | 0.115 | 0.382 | 0.756 |
| *Ornithinibacillus* | Propionate | 0.192 | 0.142 | 0.363 |
| *CAG-352* | Propionate | -0.051 | 0.699 | 0.901 |
| *UCG-001* | Propionate | -0.066 | 0.618 | 0.909 |
| *Christensenellaceae* | Propionate | -0.127 | 0.334 | 0.741 |
| *Enterorhabdus* | Propionate | -0.123 | 0.348 | 0.715 |
| *Lachnospiraceae_UCG-006* | Propionate | -0.004 | 0.977 | 0.980 |
| *Neisseriaceae* | Propionate | -0.052 | 0.695 | 0.877 |
| *Pasteurellaceae* | Propionate | 0.050 | 0.704 | 0.901 |
| *Pseudobutyrivibrio* | Propionate | -0.122 | 0.354 | 0.749 |
| *Lachnobacterium* | Propionate | -0.084 | 0.524 | 0.876 |

Abbreviations: R correlation coefficients, FDR false discovery rate.

**Table S8** Continued

| Items | Items | R | *P*-value | FDR |
| --- | --- | --- | --- | --- |
| *Ralstonia* | Propionate | -0.094 | 0.473 | 0.807 |
| *Moraxella* | Propionate | -0.131 | 0.319 | 0.756 |
| *Syntrophomonas* | Propionate | -0.108 | 0.411 | 0.801 |
| *Herbaspirillum* | Propionate | -0.200 | 0.126 | 0.662 |
| *Gastranaerophilales* | Propionate | -0.131 | 0.319 | 0.749 |
| *Prevotella_1* | Butyrate | 0.276 | 0.033 | 0.755 |
| *Mollicutes_RF39* | Butyrate | 0.139 | 0.291 | 3.349 |
| *Lachnospiraceae* | Butyrate | 0.273 | 0.035 | 0.266 |
| *Bifidobacterium* | Butyrate | 0.218 | 0.094 | 0.538 |
| *Izimaplasmatales* | Butyrate | 0.180 | 0.169 | 0.775 |
| *Bacteroidales* | Butyrate | 0.013 | 0.919 | 3.523 |
| *Ruminiclostridium_9* | Butyrate | 0.151 | 0.250 | 0.822 |
| *Ornithinibacillus* | Butyrate | 0.177 | 0.176 | 0.450 |
| *CAG-352* | Butyrate | 0.003 | 0.984 | 2.262 |
| *Bacteroidales_UCG-001* | Butyrate | -0.072 | 0.582 | 1.217 |
| *Christensenellaceae* | Butyrate | -0.132 | 0.315 | 0.604 |
| *Enterorhabdus* | Butyrate | -0.180 | 0.170 | 0.300 |
| *Lachnospiraceae_UCG-006* | Butyrate | -0.047 | 0.723 | 1.188 |
| *Neisseriaceae* | Butyrate | -0.142 | 0.279 | 0.429 |
| *Pasteurellaceae* | Butyrate | -0.031 | 0.814 | 1.170 |
| *Pseudobutyrivibrio* | Butyrate | -0.128 | 0.328 | 0.444 |
| *Lachnobacterium* | Butyrate | -0.077 | 0.558 | 0.712 |
| *Ralstonia* | Butyrate | -0.205 | 0.117 | 0.141 |
| *Moraxella* | Butyrate | -0.286 | 0.010 | 0.438 |
| *Syntrophomonas* | Butyrate | -0.152 | 0.246 | 0.270 |
| *Herbaspirillum* | Butyrate | -0.370 | 0.004 | 0.043 |
| *Gastranaerophilales* | Butyrate | -0.346 | 0.005 | 0.043 |
| *Prevotella_1* | Valerate | 0.330 | 0.010 | 0.229 |
| *Mollicutes_RF39* | Valerate | 0.153 | 0.242 | 0.788 |
| *Lachnospiraceae* | Valerate | 0.247 | 0.057 | 0.437 |
| *Bifidobacterium* | Valerate | 0.214 | 0.101 | 0.661 |
| *Izimaplasmatales* | Valerate | 0.158 | 0.228 | 0.749 |
| *Bacteroidales* | Valerate | -0.006 | 0.966 | 0.990 |
| *Ruminiclostridium_9* | Valerate | 0.126 | 0.336 | 0.751 |
| *Ornithinibacillus* | Valerate | 0.170 | 0.194 | 0.650 |
| *CAG-352* | Valerate | -0.012 | 0.929 | 0.984 |
| *Bacteroidales_UCG-001* | Valerate | -0.098 | 0.458 | 0.807 |
| *Christensenellaceae* | Valerate | -0.086 | 0.516 | 0.878 |
| *Enterorhabdus* | Valerate | -0.180 | 0.169 | 0.659 |
| *Lachnospiraceae_UCG-006* | Valerate | -0.016 | 0.906 | 0.949 |

Abbreviations: R correlation coefficients, FDR false discovery rate.

**Table S8** Continued

| Items | Items | R | *P*-value | FDR |
| --- | --- | --- | --- | --- |
| *Neisseriaceae* | Valerate | -0.070 | 0.595 | 0.913 |
| *Pasteurellaceae* | Valerate | 0.001 | 0.985 | 0.991 |
| *Pseudobutyrivibrio* | Valerate | -0.133 | 0.312 | 0.752 |
| *Lachnobacterium* | Valerate | -0.065 | 0.623 | 0.876 |
| *Ralstonia* | Valerate | -0.152 | 0.247 | 0.719 |
| *Moraxella* | Valerate | -0.200 | 0.125 | 0.664 |
| *Syntrophomonas* | Valerate | -0.106 | 0.422 | 0.462 |
| *Herbaspirillum* | Valerate | -0.255 | 0.049 | 0.091 |
| *Gastranaerophilales* | Valerate | -0.200 | 0.125 | 0.553 |
| *Prevotella_1* | TVFA | 0.337 | 0.008 | 0.193 |
| *Mollicutes_RF39* | TVFA | 0.098 | 0.458 | 0.797 |
| *Lachnospiraceae* | TVFA | 0.129 | 0.326 | 0.760 |
| *Bifidobacterium* | TVFA | 0.287 | 0.026 | 0.152 |
| *Izimaplasmatales* | TVFA | 0.070 | 0.594 | 0.875 |
| *Bacteroidales* | TVFA | 0.052 | 0.693 | 0.897 |
| *Ruminiclostridium_9* | TVFA | 0.056 | 0.670 | 0.891 |
| *Ornithinibacillus* | TVFA | 0.159 | 0.224 | 0.732 |
| *CAG-352* | TVFA | 0.157 | 0.232 | 0.714 |
| *Bacteroidales_UCG-001* | TVFA | -0.009 | 0.946 | 0.979 |
| *Christensenellaceae* | TVFA | 0.038 | 0.770 | 0.902 |
| *Enterorhabdus* | TVFA | -0.156 | 0.234 | 0.415 |
| *Lachnospiraceae_UCG-006* | TVFA | 0.014 | 0.917 | 0.966 |
| *Neisseriaceae* | TVFA | -0.128 | 0.329 | 0.504 |
| *Pasteurellaceae* | TVFA | -0.158 | 0.229 | 0.709 |
| *Pseudobutyrivibrio* | TVFA | -0.109 | 0.406 | 0.549 |
| *Lachnobacterium* | TVFA | -0.073 | 0.582 | 0.744 |
| *Ralstonia* | TVFA | 0.042 | 0.749 | 0.906 |
| *Moraxella* | TVFA | -0.148 | 0.259 | 0.728 |
| *Syntrophomonas* | TVFA | -0.063 | 0.631 | 0.871 |
| *Herbaspirillum* | TVFA | -0.094 | 0.476 | 0.828 |
| *Gastranaerophilales* | TVFA | -0.148 | 0.259 | 0.719 |
| *Prevotella_1* | Milk yield | 0.175 | 0.181 | 0.668 |
| *Mollicutes_RF39* | Milk yield | 0.255 | 0.049 | 0.563 |
| *Lachnospiraceae* | Milk yield | 0.315 | 0.014 | 0.109 |
| *Bifidobacterium* | Milk yield | 0.277 | 0.032 | 0.484 |
| *Izimaplasmatales* | Milk yield | 0.261 | 0.044 | 0.203 |
| *Bacteroidales* | Milk yield | -0.075 | 0.570 | 0.876 |
| *Ruminiclostridium_9* | Milk yield | 0.024 | 0.856 | 0.961 |
| *Ornithinibacillus* | Milk yield | 0.036 | 0.787 | 0.901 |
| *CAG-352* | Milk yield | 0.099 | 0.450 | 0.793 |

Abbreviations: R correlation coefficients, FDR false discovery rate, TVFA total volatile fatty acids.

**Table S8 Continued**

| Items | Items | R | *P*-value | FDR |
| --- | --- | --- | --- | --- |
| *Bacteroidales_UCG-001* | Milk yield | 0.066 | 0.617 | 0.879 |
| *Christensenellaceae* | Milk yield | 0.093 | 0.479 | 0.918 |
| *Enterorhabdus* | Milk yield | -0.160 | 0.223 | 0.394 |
| *Lachnospiraceae_UCG-006* | Milk yield | 0.175 | 0.181 | 0.698 |
| *Neisseriaceae* | Milk yield | -0.171 | 0.191 | 0.292 |
| *Pasteurellaceae* | Milk yield | 0.063 | 0.632 | 0.909 |
| *Pseudobutyrivibrio* | Milk yield | 0.074 | 0.577 | 0.780 |
| *Lachnobacterium* | Milk yield | 0.117 | 0.372 | 0.776 |
| *Ralstonia* | Milk yield | -0.033 | 0.803 | 0.972 |
| *Moraxella* | Milk yield | -0.078 | 0.556 | 0.876 |
| *Syntrophomonas* | Milk yield | -0.125 | 0.343 | 0.798 |
| *Herbaspirillum* | Milk yield | -0.043 | 0.745 | 0.902 |
| *Gastranaerophilales* | Milk yield | -0.078 | 0.556 | 0.876 |
| *Prevotella_1* | Lactose | 0.293 | 0.023 | 0.527 |
| *Mollicutes_RF39* | Lactose | -0.012 | 0.929 | 0.988 |
| *Lachnospiraceae* | Lactose | 0.305 | 0.018 | 0.138 |
| *Bifidobacterium* | Lactose | 0.349 | 0.006 | 0.036 |
| *Izimaplasmatales* | Lactose | 0.273 | 0.035 | 0.160 |
| *Bacteroidales* | Lactose | 0.255 | 0.049 | 0.187 |
| *Ruminiclostridium_9* | Lactose | 0.130 | 0.321 | 0.755 |
| *Ornithinibacillus* | Lactose | 0.101 | 0.443 | 0.773 |
| *CAG-352* | Lactose | 0.281 | 0.030 | 0.069 |
| *Bacteroidales_UCG-001* | Lactose | 0.030 | 0.823 | 0.942 |
| *Christensenellaceae* | Lactose | -0.077 | 0.559 | 0.877 |
| *Enterorhabdus* | Lactose | -0.188 | 0.151 | 0.667 |
| *Lachnospiraceae_UCG-006* | Lactose | -0.299 | 0.021 | 0.534 |
| *Neisseriaceae* | Lactose | -0.182 | 0.165 | 0.653 |
| *Pasteurellaceae* | Lactose | -0.354 | 0.005 | 0.039 |
| *Pseudobutyrivibrio* | Lactose | -0.223 | 0.087 | 0.118 |
| *Lachnobacterium* | Lactose | -0.110 | 0.405 | 0.817 |
| *Ralstonia* | Lactose | -0.186 | 0.156 | 0.688 |
| *Moraxella* | Lactose | -0.360 | 0.005 | 0.045 |
| *Syntrophomonas* | Lactose | -0.069 | 0.599 | 0.856 |
| *Herbaspirillum* | Lactose | -0.225 | 0.084 | 0.288 |
| *Gastranaerophilales* | Lactose | -0.360 | 0.004 | 0.043 |
| *Prevotella_1* | LA | 0.206 | 0.114 | 0.261 |
| *Mollicutes_RF39* | LA | -0.127 | 0.334 | 0.684 |
| *Lachnospiraceae* | LA | 0.018 | 0.889 | 0.918 |
| *Bifidobacterium* | LA | 0.084 | 0.526 | 0.822 |
| *Izimaplasmatales* | LA | 0.036 | 0.783 | 0.900 |
| *Bacteroidales* | LA | 0.147 | 0.261 | 0.800 |

Abbreviations: R correlation coefficients, FDR false discovery rate, LA lactic acid.

**Table S8 Continued**

| Items | Items | R | *P*-value | FDR |
| --- | --- | --- | --- | --- |
| *Ruminiclostridium_9* | LA | 0.066 | 0.615 | 0.880 |
| *Ornithinibacillus* | LA | 0.298 | 0.021 | 0.054 |
| *CAG-352* | LA | -0.032 | 0.806 | 0.935 |
| *Bacteroidales_UCG-001* | LA | 0.169 | 0.198 | 0.413 |
| *Christensenellaceae* | LA | -0.102 | 0.436 | 0.836 |
| *Enterorhabdus* | LA | -0.147 | 0.261 | 0.463 |
| *Lachnospiraceae_UCG-006* | LA | -0.261 | 0.044 | 0.072 |
| *Neisseriaceae* | LA | -0.127 | 0.335 | 0.514 |
| *Pasteurellaceae* | LA | -0.177 | 0.177 | 0.254 |
| *Pseudobutyrivibrio* | LA | -0.400 | 0.002 | 0.021 |
| *Lachnobacterium* | LA | -0.333 | 0.009 | 0.042 |
| *Ralstonia* | LA | -0.235 | 0.071 | 0.085 |
| *Moraxella* | LA | -0.292 | 0.024 | 0.027 |
| *Syntrophomonas* | LA | -0.248 | 0.056 | 0.061 |
| *Herbaspirillum* | LA | -0.347 | 0.007 | 0.040 |
| *Gastranaerophilales* | LA | -0.292 | 0.002 | 0.044 |
| *Prevotella_1* | Milk fat | -0.257 | 0.047 | 0.584 |
| *Mollicutes_RF39* | Milk fat | -0.177 | 0.177 | 0.443 |
| *Lachnospiraceae* | Milk fat | -0.156 | 0.233 | 0.719 |
| *Bifidobacterium* | Milk fat | -0.063 | 0.630 | 0.862 |
| *Izimaplasmatales* | Milk fat | -0.238 | 0.067 | 0.807 |
| *Bacteroidales* | Milk fat | -0.202 | 0.121 | 0.466 |
| *Ruminiclostridium_9* | Milk fat | -0.098 | 0.455 | 0.794 |
| *Ornithinibacillus* | Milk fat | -0.041 | 0.758 | 0.908 |
| *CAG-352* | Milk fat | -0.103 | 0.432 | 0.793 |
| *Bacteroidales_UCG-001* | Milk fat | -0.037 | 0.778 | 0.913 |
| *Christensenellaceae* | Milk fat | -0.021 | 0.874 | 0.948 |
| *Enterorhabdus* | Milk fat | 0.215 | 0.100 | 0.176 |
| *Lachnospiraceae_UCG-006* | Milk fat | 0.126 | 0.338 | 0.755 |
| *Neisseriaceae* | Milk fat | 0.022 | 0.870 | 0.963 |
| *Pasteurellaceae* | Milk fat | 0.057 | 0.668 | 0.960 |
| *Pseudobutyrivibrio* | Milk fat | 0.102 | 0.436 | 0.790 |
| *Lachnobacterium* | Milk fat | -0.005 | 0.971 | 0.994 |
| *Ralstonia* | Milk fat | 0.143 | 0.277 | 0.336 |
| *Moraxella* | Milk fat | 0.125 | 0.341 | 0.392 |
| *Syntrophomonas* | Milk fat | 0.027 | 0.840 | 0.920 |
| *Herbaspirillum* | Milk fat | 0.178 | 0.174 | 0.182 |
| *Gastranaerophilales* | Milk fat | 0.125 | 0.341 | 0.341 |
| *Prevotella_1* | Milk protein | -0.306 | 0.017 | 0.602 |
| *Mollicutes_RF39* | Milk protein | 0.177 | 0.176 | 0.627 |

Abbreviations: R correlation coefficients, FDR false discovery rate, LA lactic acid.

**Table S8 Continued**

| Items | Items | R | *P*-value | FDR |
| --- | --- | --- | --- | --- |
| *Lachnospiraceae* | Milk protein | -0.215 | 0.099 | 0.756 |
| *Bifidobacterium* | Milk protein | -0.074 | 0.576 | 0.813 |
| *Izimaplasmatales* | Milk protein | -0.116 | 0.377 | 0.733 |
| *Bacteroidales* | Milk protein | -0.174 | 0.184 | 0.706 |
| *Ruminiclostridium_9* | Milk protein | -0.107 | 0.414 | 0.859 |
| *Ornithinibacillus* | Milk protein | -0.140 | 0.285 | 0.728 |
| *CAG-352* | Milk protein | -0.148 | 0.258 | 0.593 |
| *Bacteroidales_UCG-001* | Milk protein | -0.026 | 0.843 | 0.963 |
| *Christensenellaceae* | Milk protein | 0.132 | 0.315 | 0.604 |
| *Enterorhabdus* | Milk protein | -0.060 | 0.651 | 0.875 |
| *Lachnospiraceae_UCG-006* | Milk protein | 0.409 | 0.001 | 0.039 |
| *Neisseriaceae* | Milk protein | 0.245 | 0.059 | 0.090 |
| *Pasteurellaceae* | Milk protein | 0.282 | 0.029 | 0.042 |
| *Pseudobutyrivibrio* | Milk protein | 0.268 | 0.038 | 0.052 |
| *Lachnobacterium* | Milk protein | 0.179 | 0.171 | 0.218 |
| *Ralstonia* | Milk protein | 0.210 | 0.108 | 0.130 |
| *Moraxella* | Milk protein | 0.320 | 0.301 | 0.646 |
| *Syntrophomonas* | Milk protein | -0.019 | 0.886 | 0.971 |
| *Herbaspirillum* | Milk protein | 0.266 | 0.004 | 0.041 |
| *Gastranaerophilales* | Milk protein | 0.280 | 0.002 | 0.030 |
| *Prevotella_1* | SCC | -0.382 | 0.003 | 0.060 |
| *Mollicutes_RF39* | SCC | -0.020 | 0.880 | 0.912 |
| *Lachnospiraceae* | SCC | -0.298 | 0.021 | 0.160 |
| *Bifidobacterium* | SCC | -0.274 | 0.034 | 0.196 |
| *Izimaplasmatales* | SCC | -0.384 | 0.002 | 0.011 |
| *Bacteroidales* | SCC | -0.056 | 0.669 | 0.856 |
| *Ruminiclostridium_9* | SCC | -0.168 | 0.199 | 0.655 |
| *Ornithinibacillus* | SCC | -0.205 | 0.116 | 0.296 |
| *CAG-352* | SCC | -0.119 | 0.366 | 0.842 |
| *Bacteroidales_UCG-001* | SCC | -0.036 | 0.784 | 0.938 |
| *Christensenellaceae* | SCC | 0.005 | 0.969 | 0.986 |
| *Enterorhabdus* | SCC | 0.197 | 0.132 | 0.234 |
| *Lachnospiraceae_UCG-006* | SCC | 0.284 | 0.028 | 0.145 |
| *Neisseriaceae* | SCC | 0.434 | 0.001 | 0.008 |
| *Pasteurellaceae* | SCC | 0.258 | 0.046 | 0.067 |
| *Pseudobutyrivibrio* | SCC | 0.242 | 0.062 | 0.084 |
| *Lachnobacterium* | SCC | 0.147 | 0.264 | 0.337 |
| *Ralstonia* | SCC | 0.305 | 0.018 | 0.062 |
| *Moraxella* | SCC | 0.402 | 0.000 | 0.005 |
| *Syntrophomonas* | SCC | 0.208 | 0.110 | 0.121 |
| *Herbaspirillum* | SCC | 0.294 | 0.023 | 0.024 |
| *Gastranaerophilales* | SCC | 0.402 | 0.000 | 0.004 |

Abbreviations: R correlation coefficients, FDR false discovery rate, SCC somatic cell counts.

**Table S9** Significantly different metabolites between rumen fluid samples from healthy and clinical mastitis cows, udder positive ion mode

| Metabolite | M/Z | Formula | Retention time | HMDB superclass | HMDB class | HMDB subclass^1^ | VIP^2^ | FC^3^ | *P*-value | FDR |
| --- | --- | --- | --- | --- | --- | --- | --- | --- | --- | --- |
| 10beta-Hydroxy-6beta-isobutyrylfuranoeremophilane | 321.21 | C_19_H_28_O_4_ | 7.81 | Lipids and lipid-like molecules | Prenol lipids | Sesquiterpenoids | 5.27 | 12.23 | 3.41×10^-13^ | 3.88×10^-10^ |
| 3-O-Methylniveusin A | 431.17 | C_21_H_28_O_8_ | 4.39 | Lipids and lipid-like molecules | Prenol lipids | Terpene lactones | 2.41 | 0.60 | 0.000488 | 0.007462 |
| Tetrahydrofurfuryl butyrate | 155.11 | C_9_H_16_O_3_ | 6.26 | Lipids and lipid-like molecules | Fatty Acyls | Fatty acid esters | 2.14 | 0.64 | 0.00486 | 0.02525 |
| 2-Methylglutaric acid | 210.07 | C_6_H_10_O_4_ | 0.83 | Lipids and lipid-like molecules | Fatty Acyls | Fatty acids and conjugates | 1.81 | 0.49 | 0.00029 | 0.005717 |
| LysoPE(0:0/16:0) | 436.29 | C_21_H_44_NO_7_P | 9.47 | Lipids and lipid-like molecules | Glycerophospholipids | Glycerophosphoethanolamines | 1.74 | 1.72 | 0.002489 | 0.01757 |
| 2,3-dinor, 6-keto-PGF1alpha | 343.21 | C_18_H_30_O_6_ | 6.2 | Lipids and lipid-like molecules | Fatty Acyls | Eicosanoids | 1.68 | 0.87 | 0.004388 | 0.02391 |
| 12-Oxo-2,3-dinor-10,15-phytodienoic acid | 297.21 | C_16_H_24_O_3_ | 5.74 | Lipids and lipid-like molecules | Fatty Acyls | Eicosanoids | 1.63 | 0.77 | 0.001308 | 0.01292 |
| N-Acetylcadaverine | 145.13 | C_7_H_16_N_2_O | 0.78 | Organic acids and derivatives | Carboxylic acids and derivatives | Carboxylic acid derivatives | 2.11 | 0.67 | 0.005132 | 0.02636 |
| Gamma-Glutamyltyrosine | 275.1 | C_14_H_18_N_2_O_6_ | 3.54 | Organic acids and derivatives | Carboxylic acids and derivatives | Amino acids, peptides, and analogues | 1.95 | 0.67 | 0.001385 | 0.01331 |
| 12-Hydroxydodecanoic acid | 199.17 | C_12_H_24_O_3_ | 6.09 | Organic acids and derivatives | Hydroxy acids and derivatives | Medium-chain hydroxy acids and derivatives | 1.80 | 0.79 | 0.001601 | 0.01419 |
| Valyl-Proline | 197.13 | C_10_H_18_N_2_O_3_ | 3.16 | Organic acids and derivatives | Carboxylic acids and derivatives | Amino acids, peptides, and analogues | 1.78 | 0.74 | 0.008469 | 0.03565 |
| Suberylglycine | 196.1 | C_10_H_17_NO_5_ | 2.64 | Organic acids and derivatives | Carboxylic acids and derivatives | Amino acids, peptides, and analogues | 1.45 | 0.86 | 0.000144 | 0.003824 |
| Prolyl-Valine | 197.13 | C_10_H_18_N_2_O_3_ | 2.76 | Organic acids and derivatives | Carboxylic acids and derivatives | Amino acids, peptides, and analogues | 1.37 | 0.65 | 0.02556 | 0.07059 |
| Valyl-Threonine | 251.16 | C_9_H_18_N_2_O_4_ | 0.53 | Organic acids and derivatives | Carboxylic acids and derivatives | Amino acids, peptides, and analogues | 1.18 | 0.75 | 0.08533 | 0.1677 |
| 1-Phenylethylamine | 163.12 | C_8_H_11_N | 0.81 | Organic nitrogen compounds | Organonitrogen compounds | Amines | 1.69 | 0.75 | 0.001006 | 0.01102 |
| Linoleoyl Ethanolamide | 324.29 | C_20_H_37_NO_2_ | 8.46 | Organic nitrogen compounds | Organonitrogen compounds | Amines | 1.27 | 1.09 | 0.001398 | 0.01331 |
| Jasmolone | 222.15 | C_11_H_16_O_2_ | 4.71 | Organic oxygen compounds | Organooxygen compounds | Alcohols and polyols | 2.11 | 0.71 | 0.001363 | 0.01321 |
| Mandelonitrile rutinoside | 406.15 | C_20_H_27_NO_10_ | 4.39 | Organic oxygen compounds | Organooxygen compounds | Carbohydrates and carbohydrate conjugates | 1.86 | 0.81 | 0.001064 | 0.01138 |
| (E)-2-Nonen-4-one | 141.13 | C_9_H_16_O | 5.29 | Organic oxygen compounds | Organooxygen compounds | Carbonyl compounds | 1.59 | 0.62 | 0.02457 | 0.06895 |
| 8,8-Diethoxy-2,6-dimethyl-2-octanol | 288.25 | C_14_H_30_O_3_ | 7.22 | Organic oxygen compounds | Organooxygen compounds | Alcohols and polyols | 1.48 | 1.56 | 0.003519 | 0.02119 |
| Methenamine | 141.11 | C_6_H_12_N_4_ | 0.69 | Organoheterocyclic compounds | Triazinanes | 1,3,5-triazinanes | 3.74 | 4.08 | 1.40×10^-11^ | 4.92×10^-9^ |
| 6-Methoxymellein | 209.08 | C_11_H_12_O_4_ | 7.52 | Organoheterocyclic compounds | Benzopyrans | 2-benzopyrans | 2.51 | 3.30 | 1.63×10^-10^ | 4.13×10^-8^ |
| CGMP | 346.05 | C_10_H_12_N_5_O_7_P | 1.04 | Nucleosides, nucleotides, and analogues | Purine nucleotides | Cyclic purine nucleotides | 1.13 | 1.34 | 0.1255 | 0.225 |
| Indole-3-carboxaldehyde | 146.06 | C_9_H_7_NO | 1.64 | Nucleosides, nucleotides, and analogues | Purine nucleosides | - | 0.93 | 0.90 | 0.2061 | 0.3225 |
| Deoxyinosine | 253.09 | C_10_H_12_N_4_O_4_ | 1.4 | Nucleosides, nucleotides, and analogues | Purine nucleosides | Purine 2'-deoxyribonucleosides | 0.68 | 1.06 | 0.2125 | 0.329 |
| 3-Methyl-1-phenyl-3-pentanol | 179.14 | C_12_H_18_O | 4.56 | Benzenoids | Benzene and substituted derivatives | - | 2.56 | 0.66 | 0.000407 | 0.006896 |
| P-Tolualdehyde | 121.06 | C_8_H_8_O | 0.82 | Benzenoids | Benzene and substituted derivatives | Benzoyl derivatives | 1.95 | 0.68 | 0.01546 | 0.05111 |
| Caffeoylputrescine | 251.14 | C_13_H_18_N_2_O_3_ | 3.06 | Benzenoids | Phenols | Benzenediols | 1.82 | 0.72 | 0.002922 | 0.01922 |
| P-Aminobenzoic acid | 138.05 | C_7_H_7_NO_2_ | 2.83 | Benzenoids | Benzene and substituted derivatives | Benzoic acids and derivatives | 1.81 | 0.75 | 0.003484 | 0.02109 |
| (2S,4R)-4-(9H-Pyrido[3,4-b]indol-1-yl)-1,2,4-butanetriol | 273.12 | C_15_H_16_N_2_O_3_ | 3.17 | Alkaloids and derivatives | Harmala alkaloids | - | 1.54 | 0.85 | 7.77×10^-6^ | 0.000472 |
| 3-Carboxy-2,3,4,9-tetrahydro-1H-pyrido[3,4-b]indole-1-propanoic acid | 321.14 | C_15_H_16_N_2_O_4_ | 4.21 | Alkaloids and derivatives | Harmala alkaloids | - | 1.25 | 0.85 | 0.007138 | 0.03216 |

^1^ - : no pathway information; ^2^ VIP: the contribution value of metabolites to the difference between the two groups (VIP >1); ^3^ FC: FC > 1 represents the upregulated compounds, while FC < 1 represents the downregulated compounds

Abbreviations: HMDB human metabolome database, VIP variable importance in the projection, FC fold change, FDR false discovery rate.

**Table S10** Significantly different metabolites between rumen fluid samples from healthy and clinical mastitis cows, udder negative ion mode

| Metabolite | M/Z | Formula | Retention time | HMDB superclass | HMDB class | HMDB subclass^1^ | VIP^2^ | FC^3^ | *P*-value | FDR |
| --- | --- | --- | --- | --- | --- | --- | --- | --- | --- | --- |
| 3-(4-Hydroxy-3-methoxyphenyl)-1,2-propanediol | 197.08 | C_10_H_14_O_4_ | 4.73 | Benzenoids | Phenols | Methoxyphenols | 2.27 | 0.47 | 0.0003347 | 0.005814 |
| 2-Dodecylbenzenesulfonic acid | 325.18 | C_18_H_30_O_3_S | 9.20 | Benzenoids | Benzene and substituted derivatives | Benzenesulfonic acids and derivatives | 1.48 | 1.05 | 0.0000117 | 0.0006693 |
| 2,3-Dihydroxybenzoic acid | 153.02 | C_7_H_6_O_4_ | 3.51 | Benzenoids | Benzene and substituted derivatives | Benzoic acids and derivatives | 1.38 | 0.93 | 0.002383 | 0.01568 |
| 12-oxo-20-dihydroxy-leukotriene B4 | 387.18 | C_20_H_30_O_6_ | 7.79 | Lipids and lipid-like molecules | Fatty Acyls | Eicosanoids | 4.53 | 16.36 | 3.815×10^-12^ | 5.966×10^-9^ |
| 2(R)-hydroxydocosanoic acid | 355.32 | C_22_H_44_O_3_ | 10.45 | Lipids and lipid-like molecules | Fatty Acyls | Fatty acids and conjugates | 3.12 | 2.18 | 0.0006698 | 0.008269 |
| Cis-4-Decenedioic acid | 235.07 | C_10_H_16_O_4_ | 5.44 | Lipids and lipid-like molecules | Fatty Acyls | Fatty acids and conjugates | 1.90 | 0.62 | 0.0002812 | 0.005332 |
| Jasmonic acid | 255.12 | C_12_H_18_O_3_ | 3.94 | Lipids and lipid-like molecules | Fatty Acyls | Lineolic acids and derivatives | 1.85 | 0.66 | 0.01242 | 0.04536 |
| 3-Hydroxy-5Z-octenyl acetate | 231.12 | C_10_H_18_O_3_ | 4.37 | Lipids and lipid-like molecules | Fatty Acyls | Fatty alcohol esters | 1.82 | 0.72 | 0.003294 | 0.01956 |
| 2-Ethyl-2-Hydroxybutyric acid | 131.07 | C_6_H_12_O_3_ | 3.85 | Lipids and lipid-like molecules | Fatty Acyls | Fatty acids and conjugates | 1.68 | 0.78 | 0.02616 | 0.07647 |
| DG(14:1(9Z)/22:6(4Z,7Z,10Z,13Z,16Z,19Z)/0:0) | 645.43 | C_39_H_62_O_5_ | 12.32 | Lipids and lipid-like molecules | Glycerolipids | Diradylglycerols | 1.62 | 1.41 | 0.02486 | 0.07357 |
| Adenosine 3'-monophosphate | 346.06 | C_10_H_14_N_5_O_7_P | 0.80 | Nucleosides, nucleotides, and analogues | Ribonucleoside 3'-phosphates | - | 1.48 | 1.20 | 0.01772 | 0.05867 |
| DTMP | 321.05 | C_10_H_15_N_2_O_8_P | 0.81 | Nucleosides, nucleotides, and analogues | Pyrimidine nucleotides | Pyrimidine deoxyribonucleotides | 1.07 | 1.09 | 0.01459 | 0.05091 |
| Dopaquinone | 176.03 | C_9_H_9_NO_4_ | 2.34 | Organic acids and derivatives | Carboxylic acids and derivatives | Amino acids, peptides, and analogues | 1.73 | 0.67 | 0.01996 | 0.06304 |
| (S)-9-Hydroxy-10-undecenoic acid | 199.13 | C_11_H_20_O_3_ | 6.21 | Organic acids and derivatives | Hydroxy acids and derivatives | Medium-chain hydroxy acids and derivatives | 1.73 | 0.79 | 0.003342 | 0.01979 |
| 3-Oxododecanoic acid | 259.15 | C_12_H_22_O_3_ | 5.00 | Organic acids and derivatives | Keto acids and derivatives | Medium-chain keto acids and derivatives | 1.68 | 0.78 | 0.001887 | 0.01375 |
| N-acetyl-L-2-aminoadipate(2-) | 184.06 | C_8_H_13_NO_5_ | 2.85 | Organic acids and derivatives | Carboxylic acids and derivatives | Amino acids, peptides, and analogues | 1.45 | 0.88 | 0.0008397 | 0.009212 |
| Glutaminylproline | 278.09 | C_10_H_17_N_3_O_4_ | 3.86 | Organic acids and derivatives | Carboxylic acids and derivatives | Amino acids, peptides, and analogues | 1.42 | 0.84 | 0.001919 | 0.01384 |
| Maltose | 341.11 | C_12_H_22_O_11_ | 0.65 | Organic oxygen compounds | Organooxygen compounds | Carbohydrates and carbohydrate conjugates | 1.78 | 0.79 | 0.002937 | 0.01808 |
| Beta-D-Glucopyranosyl anthranilate | 280.08 | C_13_H_17_NO_7_ | 2.91 | Organic oxygen compounds | Organooxygen compounds | Carbohydrates and carbohydrate conjugates | 1.76 | 0.66 | 0.002102 | 0.01457 |
| Talaromycin A | 229.14 | C_12_H_22_O_4_ | 4.82 | Organic oxygen compounds | Organooxygen compounds | Ethers | 1.45 | 0.88 | 0.0008217 | 0.009154 |
| 3-oxo-3-[(3,4,5,6-tetrahydroxyoxan-2-yl)methoxy]propanoic acid | 247.05 | C_9_H_14_O_9_ | 2.96 | Organic oxygen compounds | Organooxygen compounds | Carbohydrates and carbohydrate conjugates | 1.37 | 0.88 | 0.004563 | 0.02381 |
| 7-Methylhypoxanthine | 149.05 | C_6_H_6_N_4_O | 3.16 | Organoheterocyclic compounds | Imidazopyrimidines | Purines and purine derivatives | 2.22 | 0.59 | 0.00002013 | 0.0009444 |
| Alpha-Carboxy-delta-decalactone | 259.12 | C_11_H_18_O_4_ | 4.13 | Organoheterocyclic compounds | Lactones | Delta valerolactones | 1.90 | 0.73 | 0.005074 | 0.0253 |
| Cyclocalopin D | 497.16 | C_23_H_32_O_13_ | 7.66 | Organooxygen compounds | Carbohydrates and carbohydrate conjugates | Glycosyl compounds | 1.60 | 0.75 | 0.00292 | 0.01802 |
| 2-Methyl-3-(2-pentenyl)-2-cyclopenten-1-one | 209.12 | C_11_H_16_O | 5.36 | Organooxygen compounds | Carbonyl compounds | Ketones | 1.50 | 0.87 | 0.0002852 | 0.005373 |
| 4-Hydroxy-6-methyl-3-(1-oxobutyl)-2H-pyran-2-one | 195.07 | C_10_H_12_O_4_ | 2.77 | Organooxygen compounds | Carbonyl compounds | Ketones | 1.29 | 0.86 | 0.000992 | 0.009838 |
| 2-Phenylbutyric acid | 163.08 | C_10_H_12_O_2_ | 5.74 | Phenylpropanoids and polyketides | Phenylpropanoic acids | - | 2.83 | 0.46 | 2.237×10^-8^ | 4.373×10^-6^ |
| 2-Hydroxycinnamic acid | 163.04 | C_9_H_8_O_3_ | 3.88 | Phenylpropanoids and polyketides | Cinnamic acids and derivatives | Hydroxycinnamic acids and derivatives | 1.91 | 1.38 | 0.0004028 | 0.006516 |
| 3-(2-Hydroxyphenyl)propionic acid | 165.06 | C_9_H_10_O_3_ | 4.26 | Phenylpropanoids and polyketides | Phenylpropanoic acids | - | 1.39 | 0.91 | 0.0004209 | 0.006671 |

^1^ - : no pathway information; ^2^ VIP: the contribution value of metabolites to the difference between the two groups (VIP >1); ^3^ FC: FC > 1 represents the upregulated compounds, while FC < 1 represents the downregulated compounds

Abbreviations: HMDB human metabolome database, VIP variable importance in the projection, FC fold change, FDR false discovery rate.

**Table S11** Significantly different metabolites between rumen fluid samples from healthy and subclinical mastitis cows, udder positive ion mode

| Metabolite | M/Z | Formula | Retention time | HMDB superclass | HMDB class | HMDB subclass^1^ | VIP^2^ | FC^3^ | *P*-value | FDR |
| --- | --- | --- | --- | --- | --- | --- | --- | --- | --- | --- |
| Vanillin | 153.05 | C_8_H_8_O_3_ | 3.71 | Benzenoids | Phenols | Methoxyphenols | 1.24 | 0.83 | 0.1459 | 0.4371 |
| Alpha-oxycodol | 318.17 | C_18_H_23_NO_4_ | 5.55 | Benzenoids | Phenanthrenes and derivatives | - | 1.20 | 0.93 | 0.05798 | 0.274 |
| Lentialexin | 103.05 | C_8_H_8_O | 2.11 | Lipids and lipid-like molecules | Fatty Acyls | Fatty alcohols | 3.09 | 1.48 | 0.0009696 | 0.03095 |
| 10beta-Hydroxy-6beta-isobutyrylfuranoeremophilane | 321.21 | C_19_H_28_O_4_ | 7.81 | Lipids and lipid-like molecules | Prenol lipids | Sesquiterpenoids | 3.03 | 4.25 | 0.03201 | 0.208 |
| 2,3-dinor, 6-keto-PGF1alpha | 343.21 | C_18_H_30_O_6_ | 6.20 | Lipids and lipid-like molecules | Fatty Acyls | Eicosanoids | 2.51 | 0.87 | 0.003731 | 0.08494 |
| 4-Hydroxyproline galactoside | 276.11 | C_11_H_19_NO_8_ | 2.10 | Lipids and lipid-like molecules | Fatty Acyls | Fatty acyl glycosides | 2.01 | 1.10 | 0.001565 | 0.05926 |
| Withaperuvin H | 611.29 | C_30_H_42_O_9_S | 7.91 | Lipids and lipid-like molecules | Steroids and steroid derivatives | Steroid lactones | 2.00 | 0.73 | 0.1265 | 0.4078 |
| 12-Oxo-2,3-dinor-10,15-phytodienoic acid | 297.21 | C_16_H_24_O_3_ | 5.74 | Lipids and lipid-like molecules | Fatty Acyls | Eicosanoids | 2.00 | 0.87 | 0.002513 | 0.0701 |
| (3R,5Z)-5-Octene-1,3-diol | 289.24 | C_8_H_16_O_2_ | 6.62 | Lipids and lipid-like molecules | Fatty Acyls | Fatty alcohols | 1.99 | 1.26 | 0.02922 | 0.1981 |
| 3-hydroxyhexanoyl carnitine | 290.20 | C_14_H_27_NO_5_ | 3.61 | Lipids and lipid-like molecules | Fatty Acyls | Fatty acid esters | 1.98 | 0.93 | 0.002156 | 0.06779 |
| Deoxyinosine | 253.09 | C_10_H_12_N_4_O_4_ | 1.40 | Nucleosides, nucleotides, and analogues | Purine nucleosides | Purine 2'-deoxyribonucleosides | 1.85 | 1.12 | 0.01662 | 0.1536 |
| Deoxyadenosine | 252.11 | C_10_H_13_N_5_O_3_ | 1.18 | Nucleosides, nucleotides, and analogues | Purine nucleosides | - | 1.11 | 1.03 | 0.0219 | 0.1739 |
| N-(1-Deoxy-1-fructosyl)phenylalanine | 328.14 | C_15_H_21_NO_7_ | 2.13 | Organic acids and derivatives | Carboxylic acids and derivatives | Amino acids, peptides, and analogues | 2.17 | 1.17 | 0.02029 | 0.1681 |
| L-Phenylalanine | 166.09 | C_9_H_11_NO_2_ | 0.83 | Organic acids and derivatives | Carboxylic acids and derivatives | Amino acids, peptides, and analogues | 1.74 | 1.05 | 0.002804 | 0.07456 |
| L-Isoleucine | 132.10 | C_6_H_13_NO_2_ | 1.29 | Organic acids and derivatives | Carboxylic acids and derivatives | Amino acids, peptides, and analogues | 1.67 | 1.05 | 0.005987 | 0.1095 |
| Valyl-Threonine | 251.16 | C_9_H_18_N_2_O_4_ | 0.53 | Organic acids and derivatives | Carboxylic acids and derivatives | Amino acids, peptides, and analogues | 1.46 | 1.15 | 0.1387 | 0.4259 |
| L-Tyrosine | 182.08 | C_9_H_11_NO_3_ | 1.12 | Organic acids and derivatives | Carboxylic acids and derivatives | Amino acids, peptides, and analogues | 1.45 | 1.05 | 0.002158 | 0.06779 |
| Gamma-Glutamyltyrosine | 275.10 | C_14_H_18_N_2_O_6_ | 3.54 | Organic acids and derivatives | Carboxylic acids and derivatives | Amino acids, peptides, and analogues | 1.45 | 1.11 | 0.06601 | 0.2914 |
| Allysine | 187.11 | C_6_H_11_NO_3_ | 0.79 | Organic acids and derivatives | Carboxylic acids and derivatives | Amino acids, peptides, and analogues | 1.42 | 1.06 | 0.006155 | 0.1112 |
| Gamma-Glutamylvaline | 229.12 | C_10_H_18_N_2_O_5_ | 1.07 | Organic acids and derivatives | Carboxylic acids and derivatives | Amino acids, peptides, and analogues | 1.27 | 0.91 | 0.04032 | 0.232 |
| Linoleoyl Ethanolamide | 324.29 | C_20_H_37_NO_2_ | 8.46 | Organic nitrogen compounds | Organonitrogen compounds | Amines | 1.87 | 1.07 | 0.002021 | 0.06617 |
| 2-Hydroxyphenethylamine | 120.08 | C_8_H_11_NO | 0.83 | Organic nitrogen compounds | Organonitrogen compounds | Amines | 1.75 | 1.07 | 0.001332 | 0.05531 |
| 8,8-Diethoxy-2,6-dimethyl-2-octanol | 288.25 | C_14_H_30_O_3_ | 7.22 | Organic oxygen compounds | Organooxygen compounds | Alcohols and polyols | 2.33 | 1.52 | 0.02022 | 0.1681 |
| Epidermin | 294.15 | C_11_H_19_NO_6_ | 1.29 | Organic oxygen compounds | Organooxygen compounds | Carbohydrates and carbohydrate conjugates | 2.12 | 1.16 | 0.01916 | 0.1645 |
| Simmondsin | 398.14 | C_16_H_25_NO_9_ | 2.64 | Organic oxygen compounds | Organooxygen compounds | Carbohydrates and carbohydrate conjugates | 1.85 | 1.18 | 0.03339 | 0.2113 |
| Methenamine | 141.11 | C_6_H_12_N_4_ | 0.69 | Organoheterocyclic compounds | Triazinanes | 1,3,5-triazinanes | 5.15 | 4.85 | 1.068×10^-8^ | 6.951×10^-6^ |
| 6-Methoxymellein | 209.08 | C_11_H_12_O_4_ | 7.52 | Organoheterocyclic compounds | Benzopyrans | 2-benzopyrans | 3.37 | 3.23 | 5.642×10^-8^ | 2.571×10^-6^ |
| 5-Hydroxymethyl-2-furancarboxaldehyde | 159.07 | C_6_H_6_O_3_ | 3.95 | Organooxygen compounds | Carbonyl compounds | Aldehydes | 4.76 | 4.80 | 1.331×10^-9^ | 2.022×10^-6^ |
| Lotaustralin | 226.11 | C_11_H_19_NO_6_ | 2.83 | Organooxygen compounds | Carbohydrates and carbohydrate conjugates | Glycosyl compounds | 1.72 | 0.94 | 0.001361 | 0.05531 |
| Cinnamic acid | 131.05 | C_9_H_8_O_2_ | 2.09 | Phenylpropanoids and polyketides | Cinnamic acids and derivatives | Cinnamic acids | 2.75 | 1.27 | 0.0004601 | 0.04192 |
| Cajaisoflavone | 451.18 | C_26_H_26_O_7_ | 4.91 | Phenylpropanoids and polyketides | Isoflavonoids | Isoflavans | 1.73 | 0.87 | 0.04915 | 0.2521 |
| Delphinidin 3-rhamnoside 5-glucoside | 594.16 | C_27_H_31_O_16_ | 6.79 | Phenylpropanoids and polyketides | Flavonoids | Flavonoid glycosides | 1.66 | 1.12 | 0.01111 | 0.1339 |

^1^ - : no pathway information; ^2^ VIP: the contribution value of metabolites to the difference between the two groups (VIP >1); ^3^ FC: FC > 1 represents the upregulated compounds, while FC < 1 represents the downregulated compounds

Abbreviations: HMDB human metabolome database, VIP variable importance in the projection, FC fold change, FDR false discovery rate.

**Table S12** Significantly different metabolites between rumen fluid samples from healthy and subclinical mastitis cows, udder negative ion mode

| Metabolite | M/Z | Formula | Retention time | HMDB superclass | HMDB class | HMDB subclass^1^ | VIP^2^ | FC^3^ | *P*-value | FDR |
| --- | --- | --- | --- | --- | --- | --- | --- | --- | --- | --- |
| Avenestergenin A2 | 629.35 | C_37_H_52_O_7_ | 6.49 | Lipids and lipid-like molecules | Prenol lipids | Triterpenoids | 2.20 | 0.84 | 0.005158 | 0.09577 |
| Isopentyl beta-D-glucoside | 231.12 | C_11_H_22_O_6_ | 3.89 | Lipids and lipid-like molecules | Fatty Acyls | Fatty acyl glycosides | 2.15 | 0.87 | 0.004764 | 0.09377 |
| PS(18:4(6Z,9Z,12Z,15Z)/16:1(9Z)) | 798.45 | C_40_H_68_NO_10_P | 11.73 | Lipids and lipid-like molecules | Glycerophospholipids | Glycerophosphoserines | 2.05 | 1.28 | 0.004943 | 0.09447 |
| (R)-8-Acetoxycarvotanacetone | 255.12 | C_12_H_18_O_3_ | 5.25 | Lipids and lipid-like molecules | Prenol lipids | Monoterpenoids | 2.00 | 1.17 | 0.02518 | 0.1971 |
| Dolichosterone | 507.33 | C_28_H_46_O_5_ | 7.79 | Lipids and lipid-like molecules | Steroids and steroid derivatives | Bile acids, alcohols and derivatives | 1.98 | 0.66 | 0.07675 | 0.3384 |
| NONIC ACID | 187.10 | C_9_H_16_O_4_ | 5.72 | Lipids and lipid-like molecules | Fatty Acyls | Fatty acids and conjugates | 1.78 | 0.92 | 0.0007434 | 0.05936 |
| Azukisapogenol | 517.35 | C_30_H_48_O_4_ | 7.13 | Lipids and lipid-like molecules | Prenol lipids | Triterpenoids | 1.76 | 0.93 | 0.001309 | 0.06669 |
| Physagulin C | 563.23 | C_30_H_38_O_9_ | 8.10 | Lipids and lipid-like molecules | Steroids and steroid derivatives | Steroid lactones | 1.74 | 0.93 | 0.006564 | 0.1058 |
| Asparaginyl-Arginine | 287.15 | C_10_H_20_N_6_O_4_ | 4.31 | Organic acids and derivatives | Carboxylic acids and derivatives | Amino acids, peptides, and analogues | 2.15 | 1.26 | 0.04392 | 0.2519 |
| D-2-Hydroxyglutaric acid | 147.03 | C_5_H_8_O_5_ | 0.81 | Organic acids and derivatives | Hydroxy acids and derivatives | Short-chain hydroxy acids and derivatives | 2.12 | 1.16 | 0.001634 | 0.07443 |
| N-Carboxyacetyl-D-phenylalanine | 232.06 | C_12_H_13_NO_5_ | 3.64 | Organic acids and derivatives | Carboxylic acids and derivatives | Amino acids, peptides, and analogues | 1.86 | 0.88 | 0.002925 | 0.08091 |
| Glutaminyltyrosine | 308.13 | C_14_H_19_N_3_O_5_ | 5.69 | Organic acids and derivatives | Carboxylic acids and derivatives | Amino acids, peptides, and analogues | 1.65 | 0.91 | 0.01853 | 0.1701 |
| 9-Hydroxydecanoic acid | 187.13 | C_10_H_20_O_3_ | 6.21 | Organic acids and derivatives | Hydroxy acids and derivatives | Medium-chain hydroxy acids and derivatives | 1.52 | 1.10 | 0.07794 | 0.3407 |
| 8-Hydroxy-5,6-octadienoic acid | 201.08 | C_8_H_12_O_3_ | 3.17 | Organic acids and derivatives | Hydroxy acids and derivatives | Medium-chain hydroxy acids and derivatives | 1.50 | 0.93 | 0.005311 | 0.09734 |
| Oleoyl glycine | 384.28 | C_20_H_37_NO_3_ | 7.77 | Organic acids and derivatives | Carboxylic acids and derivatives | Amino acids, peptides, and analogues | 1.43 | 1.20 | 0.1526 | 0.4666 |
| Bilirubin | 605.24 | C_33_H_36_N_4_O_6_ | 8.02 | Organic compounds | Organoheterocyclic compounds | Tetrapyrroles and derivatives | 1.68 | 1.24 | 0.2291 | 0.5726 |
| Lacto-N-triaose | 580.16 | C_20_H_35_NO_16_ | 1.19 | Organic oxygen compounds | Organooxygen compounds | Carbohydrates and carbohydrate conjugates | 2.33 | 1.71 | 0.02375 | 0.1904 |
| Neuraminic acid | 266.09 | C_9_H_17_NO_8_ | 0.73 | Organic oxygen compounds | Organooxygen compounds | Carbohydrates and carbohydrate conjugates | 2.09 | 1.16 | 0.007137 | 0.1094 |
| Furaneol 4-(6-malonylglucoside) | 411.07 | C_15_H_20_O_11_ | 3.46 | Organic oxygen compounds | Organooxygen compounds | Carbohydrates and carbohydrate conjugates | 1.25 | 0.86 | 0.2087 | 0.5461 |
| D-Urobilin | 609.27 | C_33_H_40_N_4_O_6_ | 8.06 | Organoheterocyclic compounds | Tetrapyrroles and derivatives | Bilirubins | 3.14 | 0.56 | 0.009351 | 0.1232 |
| Uric acid | 167.02 | C_5_H_4_N_4_O_3_ | 0.79 | Organoheterocyclic compounds | Imidazopyrimidines | Purines and purine derivatives | 2.86 | 1.72 | 0.004635 | 0.09293 |
| 2-Isopropyl-5-methylfuran | 169.09 | C_8_H_12_O | 5.68 | Organoheterocyclic compounds | Heteroaromatic compounds | - | 2.27 | 0.85 | 0.004488 | 0.09292 |
| 1,4-Ipomeadiol | 215.09 | C_9_H_14_O_3_ | 3.54 | Organoheterocyclic compounds | Heteroaromatic compounds | - | 1.86 | 0.96 | 0.000002103 | 0.00141 |
| Sandoricin | 623.22 | C_31_H_40_O_11_ | 9.14 | Organoheterocyclic compounds | Lactones | Delta valerolactones | 1.72 | 0.90 | 0.008241 | 0.1171 |
| Cyclocalopin D | 497.16 | C_23_H_32_O_13_ | 7.66 | Organooxygen compounds | Carbohydrates and carbohydrate conjugates | Glycosyl compounds | 1.53 | 0.86 | 0.05827 | 0.2902 |
| 2-Phenylbutyric acid | 163.08 | C_10_H_12_O_2_ | 5.74 | Phenylpropanoids and polyketides | Phenylpropanoic acids | - | 2.14 | 0.72 | 0.008411 | 0.1181 |
| (R)-Byakangelicinn 2'-(3-methylbutanoate) | 453.13 | C_22_H_26_O_8_ | 7.06 | Phenylpropanoids and polyketides | Coumarins and derivatives | Furanocoumarins | 1.84 | 1.37 | 0.2241 | 0.5657 |
| 2-hydroxy-3,4-diphenylpentanedioic acid | 299.09 | C_17_H_16_O_5_ | 4.52 | Phenylpropanoids and polyketides | Stilbenes | - | 1.80 | 0.89 | 0.1269 | 0.4259 |
| 6-[(1Z)-2-hydroxy-3-oxobut-1-en-1-yl]-7-methoxy-2H-chromen-2-one | 305.07 | C_14_H_12_O_5_ | 4.56 | Phenylpropanoids and polyketides | Coumarins and derivatives | - | 1.56 | 0.88 | 0.2721 | 0.615 |
| Phenylacetic acid | 135.04 | C_8_H_8_O_2_ | 4.49 | Benzenoids | Benzene and substituted derivatives | - | 1.46 | 0.95 | 0.01542 | 0.1552 |
| 2-Dodecylbenzenesulfonic acid | 325.18 | C_18_H_30_O_3_S | 9.20 | Benzenoids | Benzene and substituted derivatives | Benzenesulfonic acids and derivatives | 1.39 | 1.03 | 0.02844 | 0.2088 |
| N-Undecylbenzenesulfonic acid | 311.17 | C_17_H_28_O_3_S | 8.59 | Benzenoids | Benzene and substituted derivatives | Benzenesulfonic acids and derivatives | 1.22 | 1.02 | 0.0629 | 0.3002 |
| Thymidine | 287.09 | C_10_H_14_N_2_O_5_ | 2.08 | Nucleosides, nucleotides, and analogues | Pyrimidine nucleosides | Pyrimidine 2'-deoxyribonucleosides | 1.47 | 1.04 | 0.002149 | 0.07653 |

^1^ - : no pathway information; ^2^ VIP: the contribution value of metabolites to the difference between the two groups (VIP >1); ^3^ FC: FC > 1 represents the upregulated compounds, while FC < 1 represents the downregulated compounds

Abbreviations: HMDB human metabolome database, VIP variable importance in the projection, FC fold change, FDR false discovery rate.

**Table S13** Significantly different metabolites between rumen fluid samples from subclinical and clinical mastitis, udder positive ion mode

| Metabolite | M/Z | Formula | Retention time | HMDB superclass | HMDB class | HMDB subclass^1^ | VIP^2^ | FC^3^ | *P*-value | FDR |
| --- | --- | --- | --- | --- | --- | --- | --- | --- | --- | --- |
| 10beta-Hydroxy-6beta-isobutyrylfuranoeremophilane | 321.21 | C_19_H_28_O_4_ | 7.81 | Lipids and lipid-like molecules | Prenol lipids | Sesquiterpenoids | 4.09 | 2.88 | 0.00000424 | 0.003226 |
| 3-O-Methylniveusin A | 431.17 | C_21_H_28_O_8_ | 4.39 | Lipids and lipid-like molecules | Prenol lipids | Terpene lactones | 2.78 | 0.58 | 0.0001988 | 0.01393 |
| Tetrahydrofurfuryl butyrate | 155.11 | C_9_H_16_O_3_ | 6.26 | Lipids and lipid-like molecules | Fatty acyls | Fatty acid esters | 2.77 | 0.59 | 0.0003336 | 0.0155 |
| (3R,5Z)-5-Octene-1,3-diol | 289.24 | C_8_H_16_O_2_ | 6.62 | Lipids and lipid-like molecules | Fatty acyls | Fatty alcohols | 2.72 | 0.51 | 0.00009973 | 0.01108 |
| Pseudoionone | 193.16 | C_13_H_20_O | 5.05 | Lipids and lipid-like molecules | Prenol lipids | Monoterpenoids | 2.59 | 0.50 | 0.000765 | 0.02004 |
| Diosbulbinoside F | 539.21 | C_26_H_34_O_12_ | 1.86 | Lipids and lipid-like molecules | Prenol lipids | Terpene glycosides | 2.55 | 0.67 | 0.0001075 | 0.01153 |
| Withaperuvin H | 611.29 | C_30_H_42_O_9_S | 7.91 | Lipids and lipid-like molecules | Steroids and steroid derivatives | Steroid lactones | 2.36 | 1.63 | 0.01505 | 0.06963 |
| Methyl geranate | 183.14 | C_11_H_18_O_2_ | 5.92 | Lipids and lipid-like molecules | Prenol lipids | Monoterpenoids | 2.34 | 0.66 | 0.0002024 | 0.01393 |
| Canesceol | 607.25 | C_29_H_44_O_11_ | 8.02 | Lipids and lipid-like molecules | Steroids and steroid derivatives | Steroid lactones | 2.24 | 0.67 | 0.01085 | 0.05865 |
| N-Acetylcadaverine | 145.13 | C_7_H_16_N_2_O | 0.78 | Organic acids and derivatives | Carboxylic acids and derivatives | Carboxylic acid derivatives | 2.73 | 0.61 | 0.0006958 | 0.01964 |
| Gamma-Glutamyltyrosine | 275.10 | C_14_H_18_N_2_O_6_ | 3.54 | Organic acids and derivatives | Carboxylic acids and derivatives | Amino acids, peptides, and analogues | 2.59 | 0.60 | 0.00005084 | 0.007994 |
| Valyl-Proline | 197.13 | C_10_H_18_N_2_O_3_ | 3.16 | Organic acids and derivatives | Carboxylic acids and derivatives | Amino acids, peptides, and analogues | 2.35 | 0.70 | 0.001723 | 0.02477 |
| N-(1-Deoxy-1-fructosyl)phenylalanine | 328.14 | C_15_H_21_NO_7_ | 2.13 | Organic acids and derivatives | Carboxylic acids and derivatives | Amino acids, peptides, and analogues | 2.25 | 0.72 | 0.001341 | 0.02338 |
| Valyl-Threonine | 251.16 | C_9_H_18_N_2_O_4_ | 0.53 | Organic acids and derivatives | Carboxylic acids and derivatives | Amino acids, peptides, and analogues | 2.25 | 0.65 | 0.002941 | 0.03127 |
| 12-Hydroxydodecanoic acid | 199.17 | C_12_H_24_O_3_ | 6.09 | Organic acids and derivatives | Hydroxy acids and derivatives | Medium-chain hydroxy acids and derivatives | 2.07 | 0.77 | 0.0004813 | 0.01705 |
| 1-Phenylethylamine | 163.12 | C_8_H_11_N | 0.81 | Organic nitrogen compounds | Organonitrogen compounds | Amines | 1.76 | 0.78 | 0.007443 | 0.0483 |
| METHACHOLINE | 160.13 | C_8_H_17_NO_2_ | 0.74 | Organic nitrogen compounds | Organonitrogen compounds | Quaternary ammonium salts | 1.24 | 0.93 | 0.004555 | 0.03707 |
| Mandelonitrile rutinoside | 406.15 | C_20_H_27_NO_10_ | 4.39 | Organic oxygen compounds | Organooxygen compounds | Carbohydrates and carbohydrate conjugates | 2.28 | 0.79 | 0.0002654 | 0.01459 |
| Simmondsin | 398.14 | C_16_H_25_NO_9_ | 2.64 | Organic oxygen compounds | Organooxygen compounds | Carbohydrates and carbohydrate conjugates | 2.25 | 0.65 | 0.0007082 | 0.01964 |
| Jasmolone | 222.15 | C_11_H_16_O_2_ | 4.71 | Organic oxygen compounds | Organooxygen compounds | Alcohols and polyols | 2.05 | 0.74 | 0.005271 | 0.04043 |
| (E,E,E)-Sylvatine | 422.21 | C_24_H_33_NO_3_ | 7.06 | Organoheterocyclic compounds | Benzodioxoles | - | 2.50 | 0.64 | 0.0001539 | 0.01323 |
| 2,6,10,10-Tetramethyl-1-oxaspiro[4.5]decan-6-ol | 213.18 | C_13_H_24_O_2_ | 4.99 | Organoheterocyclic compounds | Tetrahydrofurans | - | 2.31 | 0.52 | 0.02132 | 0.08412 |
| 2-Indolecarboxylic acid | 162.05 | C_9_H_7_NO_2_ | 3.19 | Organoheterocyclic compounds | Indoles and derivatives | Indolecarboxylic acids and derivatives | 2.21 | 0.80 | 0.0007049 | 0.01964 |
| Asteltoxin | 457.16 | C_23_H_30_O_7_ | 7.40 | Organoheterocyclic compounds | Furofurans | - | 2.15 | 0.63 | 0.02236 | 0.08639 |
| Dihydroisophorol | 143.14 | C_9_H_18_O | 5.33 | Organooxygen compounds | Alcohols and polyols | Secondary alcohols | 2.11 | 0.63 | 0.001377 | 0.02341 |
| Pantothenic Acid | 220.12 | C_9_H_17_NO_5_ | 2.67 | Organooxygen compounds | Alcohols and polyols | Polyols | 1.20 | 0.93 | 0.00342 | 0.03237 |
| 3-[2-(3,7-dimethylocta-2,6-dien-1-yl)-3,4-dihydroxyphenyl]propanoic acid | 319.19 | C_19_H_26_O_4_ | 6.38 | Phenylpropanoids and polyketides | Phenylpropanoic acids | - | 2.08 | 1.82 | 0.01386 | 0.06634 |
| 3-hydroxy-6-[(E)-2-methoxyethenyl]-7-methyl-2H-chromen-2-one | 296.09 | C_13_H_12_O_4_ | 0.74 | Phenylpropanoids and polyketides | Coumarins and derivatives | Hydroxycoumarins | 1.79 | 0.70 | 0.002548 | 0.0294 |
| Cajaisoflavone | 451.18 | C_26_H_26_O_7_ | 4.91 | Phenylpropanoids and polyketides | Isoflavonoids | Isoflavans | 1.76 | 1.23 | 0.008559 | 0.05227 |
| 3-Methyl-1-phenyl-3-pentanol | 179.14 | C_12_H_18_O | 4.56 | Benzenoids | Benzene and substituted derivatives | - | 2.87 | 0.66 | 0.0003564 | 0.01561 |
| P-Tolualdehyde | 121.06 | C_8_H_8_O | 0.82 | Benzenoids | Benzene and substituted derivatives | Benzoyl derivatives | 2.51 | 0.65 | 0.004686 | 0.03766 |
| P-Aminobenzoic acid | 138.05 | C_7_H_7_NO_2_ | 2.83 | Benzenoids | Benzene and substituted derivatives | Benzoic acids and derivatives | 2.19 | 0.74 | 0.0008674 | 0.02046 |
| Caffeoylputrescine | 251.14 | C_13_H_18_N_2_O_3_ | 3.06 | Benzenoids | Phenols | Benzenediols | 2.05 | 0.72 | 0.003031 | 0.03179 |
| 3-Carboxy-2,3,4,9-tetrahydro-1H-pyrido[3,4-b]indole-1-propanoic acid | 321.14 | C_15_H_16_N_2_O_4_ | 4.21 | Alkaloids and derivatives | Harmala alkaloids | - | 1.33 | 0.86 | 0.01931 | 0.07998 |
| (2S,4R)-4-(9H-Pyrido[3,4-b]indol-1-yl)-1,2,4-butanetriol | 273.12 | C_15_H_16_N_2_O_3_ | 3.17 | Alkaloids and derivatives | Harmala alkaloids | - | 1.17 | 0.87 | 0.006003 | 0.0435 |

^1^ - : no pathway information; ^2^ VIP: the contribution value of metabolites to the difference between the two groups (VIP >1); ^3^ FC: FC > 1 represents the upregulated compounds, while FC < 1 represents the downregulated compounds

Abbreviations: HMDB human metabolome database, VIP variable importance in the projection, FC fold change, FDR false discovery rate.

**Table S14** Significantly different metabolites between rumen fluid samples from subclinical and clinical mastitis cows, udder negative ion mode

| Metabolite | M/Z | Formula | Retention time | HMDB superclass | HMDB class | HMDB subclass^1^ | VIP^2^ | FC^3^ | *P*-value | FDR |
| --- | --- | --- | --- | --- | --- | --- | --- | --- | --- | --- |
| 12-oxo-20-dihydroxy-leukotriene B4 | 387.18 | C_20_H_30_O_6_ | 7.79 | Lipids and lipid-like molecules | Fatty acyls | Eicosanoids | 3.24 | 3.64 | 0.000002625 | 0.002052 |
| Dolichosterone | 507.33 | C_28_H_46_O_5_ | 7.79 | Lipids and lipid-like molecules | Steroids and steroid derivatives | Bile acids, alcohols and derivatives | 2.22 | 1.92 | 0.001132 | 0.01754 |
| 2(R)-hydroxydocosanoic acid | 355.32 | C_22_H_44_O_3_ | 10.45 | Lipids and lipid-like molecules | Fatty acyls | Fatty acids and conjugates | 2.09 | 1.63 | 0.01552 | 0.06155 |
| DG(14:1(9Z)/22:6(4Z,7Z,10Z,13Z,16Z,19Z)/0:0) | 645.43 | C_39_H_62_O_5_ | 12.32 | Lipids and lipid-like molecules | Glycerolipids | Diradylglycerols | 1.92 | 1.57 | 0.004145 | 0.03167 |
| Cis-4-Decenedioic acid | 235.07 | C_10_H_16_O_4_ | 5.44 | Lipids and lipid-like molecules | Fatty acyls | Fatty acids and conjugates | 2.44 | 0.58 | 0.0001581 | 0.0109 |
| Jasmonic acid | 255.12 | C_12_H_18_O_3_ | 3.94 | Lipids and lipid-like molecules | Fatty acyls | Lineolic acids and derivatives | 2.32 | 0.59 | 0.0009177 | 0.0161 |
| PS(18:4(6Z,9Z,12Z,15Z)/16:1(9Z)) | 798.45 | C_40_H_68_NO_10_P | 11.73 | Lipids and lipid-like molecules | Glycerophospholipids | Glycerophosphoserines | 1.86 | 0.66 | 0.0006019 | 0.01407 |
| Bilirubin | 605.24 | C_33_H_36_N_4_O_6_ | 8.02 | Organic compounds | Organoheterocyclic compounds | Tetrapyrroles and derivatives | 2.16 | 0.55 | 0.008956 | 0.04543 |
| Lacto-N-triaose | 580.16 | C_20_H_35_NO_16_ | 1.19 | Organic oxygen compounds | Organooxygen compounds | Carbohydrates and carbohydrate conjugates | 1.80 | 0.42 | 0.004699 | 0.03381 |
| 2-Hydroxy-3-methyl-9H-carbazole | 242.08 | C_13_H_11_NO | 5.36 | Organoheterocyclic compounds | Indoles and derivatives | Carbazoles | 2.53 | 0.56 | 0.0001213 | 0.009814 |
| Methylisopelletierine | 200.13 | C_9_H_17_NO | 4.82 | Organoheterocyclic compounds | Piperidines | - | 2.24 | 0.57 | 0.005893 | 0.03746 |
| 2-Phenylbutyric acid | 163.08 | C_10_H_12_O_2_ | 5.74 | Phenylpropanoids and polyketides | Phenylpropanoic acids | - | 1.41 | 0.64 | 0.0006232 | 0.01412 |
| D-Urobilin | 609.27 | C_33_H_40_N_4_O_6_ | 8.06 | Organoheterocyclic compounds | Tetrapyrroles and derivatives | Bilirubins | 2.26 | 2.02 | 0.00206 | 0.02263 |
| 3-(4-Hydroxy-3-methoxyphenyl)-1,2-propanediol | 197.08 | C_10_H_14_O_4_ | 4.73 | Benzenoids | Phenols | Methoxyphenols | 2.12 | 0.50 | 0.0006544 | 0.01448 |
| Pteroside Z | 415.18 | C_21_H_30_O_7_ | 7.46 | Benzenoids | Indanes | Indanones | 1.77 | 0.60 | 0.0009906 | 0.01657 |

^1^ - : no pathway information; ^2^ VIP: the contribution value of metabolites to the difference between the two groups (VIP >1); ^3^ FC: FC > 1 represents the upregulated compounds, while FC < 1 represents the downregulated compounds

Abbreviations: HMDB human metabolome database, VIP variable importance in the projection, FC fold change, FDR false discovery rate.

Table S15 Correlation analysis of differential metabolites and index of lactation and rumen fermentation

| Items | Items | R | *P*-value | FDR |
| --- | --- | --- | --- | --- |
| 10beta-Hydroxy-6beta-isobutyrylfuranoeremophilane | Acetate | -0.584 | 9.58×10-7 | 1.05×10^-5^ |
| 12-oxo-20-dihydroxy-leukotriene B4 | Acetate | -0.600 | 3.55×10-7 | 1.95×10^-5^ |
| Methenamine | Acetate | -0.376 | 0.003 | 0.392 |
| 5-Hydroxymethyl-2-furancarboxaldehyde | Acetate | -0.465 | 0.000 | 0.008 |
| 6-Methoxymellein | Acetate | -0.373 | 0.003 | 0.566 |
| Xestoaminol C | Acetate | -0.233 | 0.073 | 0.664 |
| Cinnamic acid | Acetate | -0.034 | 0.795 | 0.939 |
| Lentialexin | Acetate | -0.037 | 0.776 | 0.914 |
| 2-Phenylbutyric acid | Acetate | 0.520 | 2.06×10-5 | 0.003 |
| (3R,5Z)-5-Octene-1,3-diol | Acetate | 0.255 | 0.049 | 0.621 |
| N-Acetylcadaverine | Acetate | 0.268 | 0.039 | 0.566 |
| 10beta-Hydroxy-6beta-isobutyrylfuranoeremophilane | Propionate | -0.353 | 0.006 | 0.624 |
| 12-oxo-20-dihydroxy-leukotriene B4 | Propionate | -0.389 | 0.002 | 0.446 |
| Methenamine | Propionate | -0.003 | 0.982 | 0.999 |
| 5-Hydroxymethyl-2-furancarboxaldehyde | Propionate | -0.183 | 0.161 | 0.664 |
| 6-Methoxymellein | Propionate | 0.002 | 0.989 | 0.995 |
| Xestoaminol C | Propionate | 0.010 | 0.937 | 0.975 |
| Cinnamic acid | Propionate | -0.013 | 0.922 | 0.965 |
| Lentialexin | Propionate | 0.016 | 0.903 | 0.957 |
| 2-Phenylbutyric acid | Propionate | 0.324 | 0.011 | 0.435 |
| (3R,5Z)-5-Octene-1,3-diol | Propionate | 0.165 | 0.209 | 0.708 |
| N-Acetylcadaverine | Propionate | 0.139 | 0.289 | 0.759 |
| 10beta-Hydroxy-6beta-isobutyrylfuranoeremophilane | Butyrate | -0.472 | 1.43×10-4 | 0.046 |
| 12-oxo-20-dihydroxy-leukotriene B4 | Butyrate | -0.504 | 3.60×10-5 | 0.002 |
| Methenamine | Butyrate | -0.190 | 0.146 | 0.635 |
| 5-Hydroxymethyl-2-furancarboxaldehyde | Butyrate | -0.327 | 0.011 | 0.444 |
| 6-Methoxymellein | Butyrate | -0.194 | 0.138 | 0.663 |

Abbreviations: R correlation coefficients, FDR false discovery rate.

Table S15 Continued

| Items | Items | R | *P*-value | FDR |
| --- | --- | --- | --- | --- |
| Xestoaminol C | Butyrate | -0.122 | 0.355 | 0.710 |
| Cinnamic acid | Butyrate | -0.030 | 0.818 | 0.901 |
| Lentialexin | Butyrate | -0.016 | 0.906 | 0.958 |
| 2-Phenylbutyric acid | Butyrate | 0.422 | 0.001 | 0.392 |
| (3R,5Z)-5-Octene-1,3-diol | Butyrate | 0.199 | 0.128 | 0.641 |
| N-Acetylcadaverine | Butyrate | 0.217 | 0.095 | 0.665 |
| 10beta-Hydroxy-6beta-isobutyrylfuranoeremophilane | Valerate | -0.419 | 0.001 | 0.009 |
| 12-oxo-20-dihydroxy-leukotriene B4 | Valerate | -0.446 | 0.000 | 0.002 |
| Methenamine | Valerate | -0.143 | 0.274 | 1.005 |
| 5-Hydroxymethyl-2-furancarboxaldehyde | Valerate | -0.303 | 0.019 | 0.051 |
| 6-Methoxymellein | Valerate | -0.138 | 0.295 | 0.648 |
| Xestoaminol C | Valerate | -0.072 | 0.582 | 1.067 |
| Cinnamic acid | Valerate | -0.052 | 0.693 | 1.088 |
| Lentialexin | Valerate | -0.038 | 0.773 | 1.062 |
| 2-Phenylbutyric acid | Valerate | 0.401 | 0.001 | 0.002 |
| (3R,5Z)-5-Octene-1,3-diol | Valerate | 0.155 | 0.237 | 0.261 |
| N-Acetylcadaverine | Valerate | 0.168 | 0.198 | 0.198 |
| 10beta-Hydroxy-6beta-isobutyrylfuranoeremophilane | TVFA | -0.318 | 0.013 | 0.435 |
| 12-oxo-20-dihydroxy-leukotriene B4 | TVFA | -0.317 | 0.014 | 0.434 |
| Methenamine | TVFA | -0.256 | 0.048 | 0.621 |
| 5-Hydroxymethyl-2-furancarboxaldehyde | TVFA | -0.228 | 0.080 | 0.666 |
| 6-Methoxymellein | TVFA | -0.189 | 0.147 | 0.663 |
| Xestoaminol C | TVFA | -0.225 | 0.084 | 0.666 |
| Cinnamic acid | TVFA | 0.102 | 0.438 | 0.800 |
| Lentialexin | TVFA | 0.148 | 0.258 | 0.725 |
| 2-Phenylbutyric acid | TVFA | 0.252 | 0.052 | 0.635 |
| (3R,5Z)-5-Octene-1,3-diol | TVFA | 0.193 | 0.139 | 0.663 |
| N-Acetylcadaverine | TVFA | 0.158 | 0.228 | 0.713 |
| 10beta-Hydroxy-6beta-isobutyrylfuranoeremophilane | Milk yield | -0.237 | 0.068 | 0.656 |
| 12-oxo-20-dihydroxy-leukotriene B4 | Milk yield | -0.226 | 0.083 | 0.664 |
| Methenamine | Milk yield | -0.378 | 0.003 | 0.446 |
| 5-Hydroxymethyl-2-furancarboxaldehyde | Milk yield | -0.473 | 0.000 | 0.079 |
| 6-Methoxymellein | Milk yield | -0.345 | 0.007 | 0.655 |
| Xestoaminol C | Milk yield | -0.242 | 0.062 | 0.657 |
| Cinnamic acid | Milk yield | -0.126 | 0.338 | 0.711 |
| Lentialexin | Milk yield | -0.124 | 0.344 | 0.716 |
| 2-Phenylbutyric acid | Milk yield | 0.383 | 0.003 | 0.446 |
| (3R,5Z)-5-Octene-1,3-diol | Milk yield | 0.017 | 0.896 | 0.947 |
| N-Acetylcadaverine | Milk yield | -0.022 | 0.867 | 0.947 |
| 10beta-Hydroxy-6beta-isobutyrylfuranoeremophilane | Lactose | -0.700 | 4.83×10-10 | 5.32×10^-8^ |
| 12-oxo-20-dihydroxy-leukotriene B4 | Lactose | -0.688 | 1.24×10-9 | 6.81×10^-7^ |
| Methenamine | Lactose | -0.515 | 2.57×10-4 | 0.094 |

Abbreviations: R correlation coefficients, FDR false discovery rate.

Table S15 Continued

| Items | Items | R | *P*-value | FDR |
| --- | --- | --- | --- | --- |
| 5-Hydroxymethyl-2-furancarboxaldehyde | Lactose | -0.537 | 9.56×10-6 | 2.63×10^-4^ |
| 6-Methoxymellein | Lactose | -0.542 | 7.58×10-6 | 1.67×10^-4^ |
| Xestoaminol C | Lactose | -0.501 | 0.005 | 0.606 |
| Cinnamic acid | Lactose | -0.018 | 0.894 | 0.940 |
| Lentialexin | Lactose | -0.017 | 0.895 | 0.943 |
| 2-Phenylbutyric acid | Lactose | 0.547 | 6.26×10-6 | 7.66×10-4 |
| (3R,5Z)-5-Octene-1,3-diol | Lactose | 0.409 | 0.001 | 0.203 |
| N-Acetylcadaverine | Lactose | 0.480 | 0.000 | 0.065 |
| 10beta-Hydroxy-6beta-isobutyrylfuranoeremophilane | LA | -0.422 | 0.001 | 0.109 |
| 12-oxo-20-dihydroxy-leukotriene B4 | LA | -0.401 | 0.001 | 0.202 |
| Methenamine | LA | -0.271 | 0.036 | 0.621 |
| 5-Hydroxymethyl-2-furancarboxaldehyde | LA | -0.167 | 0.202 | 0.663 |
| 6-Methoxymellein | LA | -0.332 | 0.010 | 0.408 |
| Xestoaminol C | LA | -0.109 | 0.408 | 0.718 |
| Cinnamic acid | LA | 0.261 | 0.044 | 0.621 |
| Lentialexin | LA | 0.227 | 0.081 | 0.111 |
| 2-Phenylbutyric acid | LA | 0.206 | 0.114 | 0.663 |
| (3R,5Z)-5-Octene-1,3-diol | LA | 0.612 | 2.03×10-7 | 4.46×10^-5^ |
| N-Acetylcadaverine | LA | 0.670 | 4.84×10-9 | 6.21×10^-7^ |
| 10beta-Hydroxy-6beta-isobutyrylfuranoeremophilane | Milk fat | -0.329 | 0.010 | 0.443 |
| 12-oxo-20-dihydroxy-leukotriene B4 | Milk fat | -0.322 | 0.012 | 0.445 |
| Methenamine | Milk fat | -0.458 | 0.000 | 0.001 |
| 5-Hydroxymethyl-2-furancarboxaldehyde | Milk fat | -0.604 | 0.000 | 0.000 |
| 6-Methoxymellein | Milk fat | -0.466 | 0.000 | 0.000 |
| Xestoaminol C | Milk fat | -0.284 | 0.028 | 0.051 |
| Cinnamic acid | Milk fat | -0.055 | 0.679 | 0.891 |
| Lentialexin | Milk fat | -0.092 | 0.484 | 0.841 |
| 2-Phenylbutyric acid | Milk fat | 0.442 | 0.000 | 0.083 |
| (3R,5Z)-5-Octene-1,3-diol | Milk fat | 0.177 | 0.177 | 0.443 |
| N-Acetylcadaverine | Milk fat | 0.204 | 0.118 | 0.446 |
| 10beta-Hydroxy-6beta-isobutyrylfuranoeremophilane | Milk protein | 0.465 | 0.000 | 0.052 |
| 12-oxo-20-dihydroxy-leukotriene B4 | Milk protein | 0.445 | 0.000 | 0.081 |
| Methenamine | Milk protein | 0.390 | 0.002 | 0.446 |
| 5-Hydroxymethyl-2-furancarboxaldehyde | Milk protein | 0.323 | 0.012 | 0.433 |
| 6-Methoxymellein | Milk protein | 0.484 | 8.98×10-4 | 0.070 |
| Xestoaminol C | Milk protein | 0.364 | 0.004 | 0.621 |
| Cinnamic acid | Milk protein | -0.188 | 0.151 | 0.648 |
| Lentialexin | Milk protein | -0.152 | 0.246 | 0.708 |
| 2-Phenylbutyric acid | Milk protein | -0.428 | 0.001 | 0.110 |
| (3R,5Z)-5-Octene-1,3-diol | Milk protein | -0.606 | 2.92×10-7 | 7.21×10^-5^ |
| N-Acetylcadaverine | Milk protein | -0.582 | 1.07×10-6 | 6.66×10^-4^ |
| 10beta-Hydroxy-6beta-isobutyrylfuranoeremophilane | SCC | 0.716 | 1.23×10-10 | 1.36×10^-8^ |

Abbreviations: R correlation coefficients, FDR false discovery rate, LA lactic acid.

Table S15 Continued

| Items | Items | R | *P*-value | FDR |
| --- | --- | --- | --- | --- |
| 12-oxo-20-dihydroxy-leukotriene B4 | SCC | 0.712 | 1.80×10-10 | 6.62×10^-8^ |
| Methenamine | SCC | 0.603 | 3.50×10-7 | 1.29×10^-5^ |
| 5-Hydroxymethyl-2-furancarboxaldehyde | SCC | 0.550 | 5.28×10-6 | 1.45×10^-4^ |
| 6-Methoxymellein | SCC | 0.599 | 4.29×10-7 | 7.14×10^-5^ |
| Xestoaminol C | SCC | 0.453 | 0.001 | 0.071 |
| Cinnamic acid | SCC | -0.112 | 0.394 | 0.670 |
| Lentialexin | SCC | -0.070 | 0.596 | 0.875 |
| 2-Phenylbutyric acid | SCC | -0.573 | 1.70×10-6 | 2.08×10^-4^ |
| (3R,5Z)-5-Octene-1,3-diol | SCC | -0.383 | 0.003 | 0.236 |
| N-Acetylcadaverine | SCC | -0.409 | 0.001 | 0.202 |

Abbreviations: R correlation coefficients, FDR false discovery rate, SCC somatic cell counts.

Table S16 Correlation analysis of differential bacteria genus and differential metabolites in rumen samples

| Items | Items | R | *P*-value | FDR |
| --- | --- | --- | --- | --- |
| *Prevotella_1* | 10beta-Hydroxy-6beta-isobutyrylfuranoeremophilane | -0.290 | 0.024 | 0.460 |
| *Mollicutes_RF39* | 10beta-Hydroxy-6beta-isobutyrylfuranoeremophilane | -0.084 | 0.522 | 0.863 |
| *Lachnospiraceae* | 10beta-Hydroxy-6beta-isobutyrylfuranoeremophilane | -0.236 | 0.069 | 0.632 |
| *Bifidobacterium* | 10beta-Hydroxy-6beta-isobutyrylfuranoeremophilane | -0.214 | 0.100 | 0.667 |
| *Izimaplasmatales* | 10beta-Hydroxy-6beta-isobutyrylfuranoeremophilane | -0.274 | 0.034 | 0.758 |
| *Bacteroidales* | 10beta-Hydroxy-6beta-isobutyrylfuranoeremophilane | -0.226 | 0.082 | 0.664 |
| *Ruminiclostridium_9* | 10beta-Hydroxy-6beta-isobutyrylfuranoeremophilane | -0.134 | 0.308 | 0.751 |
| *Ornithinibacillus* | 10beta-Hydroxy-6beta-isobutyrylfuranoeremophilane | -0.122 | 0.352 | 0.739 |
| *CAG-352* | 10beta-Hydroxy-6beta-isobutyrylfuranoeremophilane | -0.216 | 0.097 | 0.663 |
| *Bacteroidales_UCG-001* | 10beta-Hydroxy-6beta-isobutyrylfuranoeremophilane | -0.107 | 0.415 | 0.867 |
| *Christensenellaceae* | 10beta-Hydroxy-6beta-isobutyrylfuranoeremophilane | -0.044 | 0.737 | 0.901 |
| *Enterorhabdus* | 10beta-Hydroxy-6beta-isobutyrylfuranoeremophilane | 0.158 | 0.228 | 0.403 |
| *Lachnospiraceae_UCG-006* | 10beta-Hydroxy-6beta-isobutyrylfuranoeremophilane | 0.493 | 0.001 | 0.038 |
| *Neisseriaceae* | 10beta-Hydroxy-6beta-isobutyrylfuranoeremophilane | 0.493 | 0.041 | 0.062 |
| *Pasteurellaceae* | 10beta-Hydroxy-6beta-isobutyrylfuranoeremophilane | 0.565 | 0.003 | 0.043 |
| *Pseudobutyrivibrio* | 10beta-Hydroxy-6beta-isobutyrylfuranoeremophilane | 0.417 | 0.096 | 0.660 |
| *Lachnobacterium* | 10beta-Hydroxy-6beta-isobutyrylfuranoeremophilane | 0.493 | 0.481 | 0.864 |
| *Ralstonia* | 10beta-Hydroxy-6beta-isobutyrylfuranoeremophilane | 0.546 | 0.022 | 0.426 |
| *Moraxella* | 10beta-Hydroxy-6beta-isobutyrylfuranoeremophilane | 0.599 | 0.001 | 0.033 |
| *Syntrophomonas* | 10beta-Hydroxy-6beta-isobutyrylfuranoeremophilane | 0.356 | 0.966 | 0.978 |
| *Herbaspirillum* | 10beta-Hydroxy-6beta-isobutyrylfuranoeremophilane | 0.349 | 0.255 | 0.467 |
| *Gastranaerophilales* | 10beta-Hydroxy-6beta-isobutyrylfuranoeremophilane | 0.589 | 0.001 | 0.031 |
| *Prevotella_1* | 12-oxo-20-dihydroxy-leukotriene B4 | -0.220 | 0.091 | 0.669 |
| *Mollicutes_RF39* | 12-oxo-20-dihydroxy-leukotriene B4 | -0.340 | 0.761 | 0.902 |
| *Lachnospiraceae* | 12-oxo-20-dihydroxy-leukotriene B4 | -0.304 | 0.118 | 0.662 |

Abbreviations: R correlation coefficients, FDR false discovery rate.

Table S16 Continued

| Items | Items | R | *P*-value | FDR |
| --- | --- | --- | --- | --- |
| *Bifidobacterium* | 12-oxo-20-dihydroxy-leukotriene B4 | -0.278 | 0.174 | 0.670 |
| *Izimaplasmatales* | 12-oxo-20-dihydroxy-leukotriene B4 | -0.361 | 0.044 | 0.621 |
| *Bacteroidales* | 12-oxo-20-dihydroxy-leukotriene B4 | -0.328 | 0.080 | 0.667 |
| *Ruminiclostridium_9* | 12-oxo-20-dihydroxy-leukotriene B4 | -0.229 | 0.326 | 0.745 |
| *Ornithinibacillus* | 12-oxo-20-dihydroxy-leukotriene B4 | -0.193 | 0.481 | 0.859 |
| *CAG-352* | 12-oxo-20-dihydroxy-leukotriene B4 | -0.284 | 0.160 | 0.648 |
| *Bacteroidales_UCG-001* | 12-oxo-20-dihydroxy-leukotriene B4 | -0.187 | 0.508 | 0.873 |
| *Christensenellaceae* | 12-oxo-20-dihydroxy-leukotriene B4 | -0.118 | 0.889 | 0.944 |
| *Enterorhabdus* | 12-oxo-20-dihydroxy-leukotriene B4 | 0.147 | 0.262 | 0.733 |
| *Lachnospiraceae_UCG-006* | 12-oxo-20-dihydroxy-leukotriene B4 | 0.542 | 0.003 | 0.042 |
| *Neisseriaceae* | 12-oxo-20-dihydroxy-leukotriene B4 | 0.438 | 0.067 | 0.653 |
| *Pasteurellaceae* | 12-oxo-20-dihydroxy-leukotriene B4 | 0.478 | 0.032 | 0.625 |
| *Pseudobutyrivibrio* | 12-oxo-20-dihydroxy-leukotriene B4 | 0.449 | 0.055 | 0.647 |
| *Lachnobacterium* | 12-oxo-20-dihydroxy-leukotriene B4 | 0.337 | 0.296 | 0.738 |
| *Ralstonia* | 12-oxo-20-dihydroxy-leukotriene B4 | 0.420 | 0.013 | 0.205 |
| *Moraxella* | 12-oxo-20-dihydroxy-leukotriene B4 | 0.574 | 0.001 | 0.038 |
| *Syntrophomonas* | 12-oxo-20-dihydroxy-leukotriene B4 | 0.035 | 0.790 | 0.902 |
| *Herbaspirillum* | 12-oxo-20-dihydroxy-leukotriene B4 | 0.165 | 0.208 | 0.707 |
| *Gastranaerophilales* | 12-oxo-20-dihydroxy-leukotriene B4 | 0.561 | 0.001 | 0.037 |
| *Prevotella_1* | Methenamine | -0.359 | 0.005 | 0.517 |
| *Mollicutes_RF39* | Methenamine | -0.183 | 0.161 | 0.645 |
| *Lachnospiraceae* | Methenamine | -0.297 | 0.021 | 0.431 |
| *Bifidobacterium* | Methenamine | -0.187 | 0.152 | 0.874 |
| *Izimaplasmatales* | Methenamine | -0.577 | 0.001 | 0.041 |
| *Bacteroidales* | Methenamine | -0.234 | 0.071 | 0.662 |
| *Ruminiclostridium_9* | Methenamine | -0.117 | 0.895 | 0.942 |
| *Ornithinibacillus* | Methenamine | 0.153 | 0.686 | 0.901 |
| *CAG-352* | Methenamine | -0.460 | 0.005 | 0.061 |
| *Bacteroidales_UCG-001* | Methenamine | 0.003 | 0.982 | 0.994 |
| *Christensenellaceae* | Methenamine | -0.337 | 0.069 | 0.881 |
| *Enterorhabdus* | Methenamine | 0.309 | 0.110 | 0.674 |
| *Lachnospiraceae_UCG-006* | Methenamine | 0.155 | 0.678 | 0.894 |
| *Neisseriaceae* | Methenamine | 0.303 | 0.121 | 0.665 |
| *Pasteurellaceae* | Methenamine | 0.338 | 0.067 | 0.096 |
| *Pseudobutyrivibrio* | Methenamine | 0.116 | 0.904 | 0.954 |
| *Lachnobacterium* | Methenamine | -0.392 | 0.484 | 0.839 |
| *Ralstonia* | Methenamine | 0.334 | 0.072 | 0.087 |
| *Moraxella* | Methenamine | 0.430 | 0.010 | 0.012 |
| *Syntrophomonas* | Methenamine | 0.210 | 0.404 | 0.753 |
| *Herbaspirillum* | Methenamine | 0.255 | 0.237 | 0.708 |

Abbreviations: R correlation coefficients, FDR false discovery rate.

Table S16 Continued

| Items | Items | R | *P*-value | FDR |
| --- | --- | --- | --- | --- |
| *Gastranaerophilales* | Methenamine | 0.430 | 0.010 | 0.430 |
| *Prevotella_1* | 5-Hydroxymethyl-2-furancarboxaldehyde | -0.388 | 0.003 | 0.056 |
| *Mollicutes_RF39* | 5-Hydroxymethyl-2-furancarboxaldehyde | -0.140 | 0.286 | 0.704 |
| *Lachnospiraceae* | 5-Hydroxymethyl-2-furancarboxaldehyde | -0.183 | 0.162 | 0.644 |
| *Bifidobacterium* | 5-Hydroxymethyl-2-furancarboxaldehyde | -0.273 | 0.035 | 0.561 |
| *Izimaplasmatales* | 5-Hydroxymethyl-2-furancarboxaldehyde | -0.309 | 0.016 | 0.075 |
| *Bacteroidales* | 5-Hydroxymethyl-2-furancarboxaldehyde | -0.115 | 0.380 | 0.768 |
| *Ruminiclostridium_9* | 5-Hydroxymethyl-2-furancarboxaldehyde | 0.544 | 0.385 | 0.765 |
| *Ornithinibacillus* | 5-Hydroxymethyl-2-furancarboxaldehyde | 0.236 | 0.783 | 0.928 |
| *CAG-352* | 5-Hydroxymethyl-2-furancarboxaldehyde | -0.122 | 0.352 | 0.810 |
| *Bacteroidales_UCG-001* | 5-Hydroxymethyl-2-furancarboxaldehyde | -0.237 | 0.778 | 0.902 |
| *Christensenellaceae* | 5-Hydroxymethyl-2-furancarboxaldehyde | -0.033 | 0.802 | 0.930 |
| *Enterorhabdus* | 5-Hydroxymethyl-2-furancarboxaldehyde | 0.527 | 0.001 | 0.047 |
| *Lachnospiraceae_UCG-006* | 5-Hydroxymethyl-2-furancarboxaldehyde | 0.286 | 0.515 | 0.846 |
| *Neisseriaceae* | 5-Hydroxymethyl-2-furancarboxaldehyde | 0.057 | 0.666 | 0.875 |
| *Pasteurellaceae* | 5-Hydroxymethyl-2-furancarboxaldehyde | 0.286 | 0.155 | 0.652 |
| *Pseudobutyrivibrio* | 5-Hydroxymethyl-2-furancarboxaldehyde | 0.283 | 0.527 | 0.871 |
| *Lachnobacterium* | 5-Hydroxymethyl-2-furancarboxaldehyde | 0.208 | 0.954 | 0.979 |
| *Ralstonia* | 5-Hydroxymethyl-2-furancarboxaldehyde | 0.307 | 0.113 | 0.657 |
| *Moraxella* | 5-Hydroxymethyl-2-furancarboxaldehyde | 0.258 | 0.047 | 0.624 |
| *Syntrophomonas* | 5-Hydroxymethyl-2-furancarboxaldehyde | 0.101 | 0.442 | 0.774 |
| *Herbaspirillum* | 5-Hydroxymethyl-2-furancarboxaldehyde | 0.156 | 0.233 | 0.714 |
| *Gastranaerophilales* | 5-Hydroxymethyl-2-furancarboxaldehyde | 0.258 | 0.047 | 0.627 |
| *Prevotella_1* | 6-Methoxymellein | -0.349 | 0.006 | 0.149 |
| *Mollicutes_RF39* | 6-Methoxymellein | -0.075 | 0.567 | 0.875 |
| *Lachnospiraceae* | 6-Methoxymellein | -0.263 | 0.043 | 0.621 |
| *Bifidobacterium* | 6-Methoxymellein | -0.128 | 0.330 | 0.739 |
| *Izimaplasmatales* | 6-Methoxymellein | -0.499 | 5.03×10-5 | 0.002 |
| *Bacteroidales* | 6-Methoxymellein | -0.223 | 0.087 | 0.333 |
| *Ruminiclostridium_9* | 6-Methoxymellein | -0.085 | 0.519 | 0.875 |
| *Ornithinibacillus* | 6-Methoxymellein | 0.119 | 0.365 | 0.932 |
| *CAG-352* | 6-Methoxymellein | -0.247 | 0.057 | 0.131 |
| *Bacteroidales_UCG-001* | 6-Methoxymellein | 0.025 | 0.851 | 0.949 |
| *Christensenellaceae* | 6-Methoxymellein | -0.147 | 0.263 | 0.723 |
| *Enterorhabdus* | 6-Methoxymellein | 0.138 | 0.294 | 0.754 |
| *Lachnospiraceae_UCG-006* | 6-Methoxymellein | 0.090 | 0.494 | 0.851 |
| *Neisseriaceae* | 6-Methoxymellein | 0.161 | 0.219 | 0.709 |
| *Pasteurellaceae* | 6-Methoxymellein | 0.319 | 0.013 | 0.045 |
| *Pseudobutyrivibrio* | 6-Methoxymellein | 0.151 | 0.250 | 0.719 |
| *Lachnobacterium* | 6-Methoxymellein | 0.016 | 0.903 | 0.952 |

Abbreviations: R correlation coefficients, FDR false discovery rate.

Table S16 Continued

| Items | Items | R | *P*-value | FDR |
| --- | --- | --- | --- | --- |
| *Ralstonia* | 6-Methoxymellein | 0.274 | 0.034 | 0.621 |
| *Moraxella* | 6-Methoxymellein | 0.361 | 0.005 | 0.215 |
| *Syntrophomonas* | 6-Methoxymellein | 0.168 | 0.199 | 0.687 |
| *Herbaspirillum* | 6-Methoxymellein | 0.205 | 0.115 | 0.602 |
| *Gastranaerophilales* | 6-Methoxymellein | 0.361 | 0.005 | 0.085 |
| *Prevotella_1* | Xestoaminol C | -0.271 | 0.036 | 0.556 |
| *Mollicutes_RF39* | Xestoaminol C | -0.117 | 0.373 | 0.758 |
| *Lachnospiraceae* | Xestoaminol C | -0.322 | 0.012 | 0.437 |
| *Bifidobacterium* | Xestoaminol C | -0.107 | 0.418 | 0.750 |
| *Izimaplasmatales* | Xestoaminol C | -0.277 | 0.032 | 0.549 |
| *Bacteroidales* | Xestoaminol C | -0.152 | 0.247 | 0.707 |
| *Ruminiclostridium_9* | Xestoaminol C | -0.067 | 0.612 | 0.875 |
| *Ornithinibacillus* | Xestoaminol C | 0.117 | 0.372 | 0.755 |
| *CAG-352* | Xestoaminol C | -0.508 | 0.003 | 0.057 |
| *Bacteroidales_UCG-001* | Xestoaminol C | -0.046 | 0.724 | 0.922 |
| *Christensenellaceae* | Xestoaminol C | -0.237 | 0.069 | 0.652 |
| *Enterorhabdus* | Xestoaminol C | 0.001 | 0.989 | 0.994 |
| *Lachnospiraceae_UCG-006* | Xestoaminol C | 0.015 | 0.912 | 0.966 |
| *Neisseriaceae* | Xestoaminol C | 0.279 | 0.031 | 0.548 |
| *Pasteurellaceae* | Xestoaminol C | 0.232 | 0.074 | 0.668 |
| *Pseudobutyrivibrio* | Xestoaminol C | -0.086 | 0.513 | 0.876 |
| *Lachnobacterium* | Xestoaminol C | -0.243 | 0.061 | 0.650 |
| *Ralstonia* | Xestoaminol C | 0.161 | 0.220 | 0.709 |
| *Moraxella* | Xestoaminol C | 0.340 | 0.008 | 0.243 |
| *Syntrophomonas* | Xestoaminol C | -0.132 | 0.314 | 0.714 |
| *Herbaspirillum* | Xestoaminol C | 0.071 | 0.591 | 0.876 |
| *Gastranaerophilales* | Xestoaminol C | 0.340 | 0.008 | 0.679 |
| *Prevotella_1* | Cinnamic acid | -0.134 | 0.796 | 0.910 |
| *Mollicutes_RF39* | Cinnamic acid | -0.236 | 0.300 | 0.759 |
| *Lachnospiraceae* | Cinnamic acid | -0.460 | 0.005 | 0.621 |
| *Bifidobacterium* | Cinnamic acid | -0.189 | 0.500 | 0.804 |
| *Izimaplasmatales* | Cinnamic acid | -0.292 | 0.142 | 0.651 |
| *Bacteroidales* | Cinnamic acid | -0.258 | 0.228 | 0.714 |
| *Ruminiclostridium_9* | Cinnamic acid | 0.323 | 0.087 | 0.655 |
| *Ornithinibacillus* | Cinnamic acid | -0.083 | 0.528 | 0.875 |
| *CAG-352* | Cinnamic acid | -0.334 | 0.072 | 0.657 |
| *Bacteroidales_UCG-001* | Cinnamic acid | -0.026 | 0.963 | 0.995 |
| *Christensenellaceae* | Cinnamic acid | -0.076 | 0.564 | 0.875 |
| *Enterorhabdus* | Cinnamic acid | -0.232 | 0.315 | 0.704 |
| *Lachnospiraceae_UCG-006* | Cinnamic acid | -0.353 | 0.244 | 0.664 |

Abbreviations: R correlation coefficients, FDR false discovery rate.

Table S16 Continued

| Items | Items | R | *P*-value | FDR |
| --- | --- | --- | --- | --- |
| *Neisseriaceae* | Cinnamic acid | -0.380 | 0.169 | 0.650 |
| *Pasteurellaceae* | Cinnamic acid | 0.185 | 0.519 | 0.876 |
| *Pseudobutyrivibrio* | Cinnamic acid | -0.183 | 0.528 | 0.875 |
| *Lachnobacterium* | Cinnamic acid | -0.233 | 0.310 | 0.746 |
| *Ralstonia* | Cinnamic acid | -0.296 | 0.466 | 0.816 |
| *Moraxella* | Cinnamic acid | -0.115 | 0.912 | 0.957 |
| *Syntrophomonas* | Cinnamic acid | -0.062 | 0.637 | 0.882 |
| *Herbaspirillum* | Cinnamic acid | -0.215 | 0.380 | 0.758 |
| *Gastranaerophilales* | Cinnamic acid | -0.115 | 0.912 | 0.952 |
| *Prevotella_1* | Lentialexin | -0.169 | 0.599 | 0.874 |
| *Mollicutes_RF39* | Lentialexin | -0.247 | 0.263 | 0.710 |
| *Lachnospiraceae* | Lentialexin | -0.412 | 0.015 | 0.118 |
| *Bifidobacterium* | Lentialexin | -0.201 | 0.441 | 0.788 |
| *Izimaplasmatales* | Lentialexin | -0.343 | 0.061 | 0.652 |
| *Bacteroidales* | Lentialexin | -0.270 | 0.193 | 0.441 |
| *Ruminiclostridium_9* | Lentialexin | 0.286 | 0.155 | 0.670 |
| *Ornithinibacillus* | Lentialexin | -0.154 | 0.684 | 0.901 |
| *CAG-352* | Lentialexin | -0.257 | 0.231 | 0.711 |
| *Bacteroidales_UCG-001* | Lentialexin | 0.136 | 0.786 | 0.928 |
| *Christensenellaceae* | Lentialexin | -0.130 | 0.943 | 0.981 |
| *Enterorhabdus* | Lentialexin | -0.341 | 0.284 | 0.750 |
| *Lachnospiraceae_UCG-006* | Lentialexin | -0.243 | 0.276 | 0.744 |
| *Neisseriaceae* | Lentialexin | -0.252 | 0.245 | 0.716 |
| *Pasteurellaceae* | Lentialexin | 0.208 | 0.414 | 0.774 |
| *Pseudobutyrivibrio* | Lentialexin | -0.145 | 0.732 | 0.901 |
| *Lachnobacterium* | Lentialexin | -0.180 | 0.541 | 0.872 |
| *Ralstonia* | Lentialexin | -0.146 | 0.728 | 0.902 |
| *Moraxella* | Lentialexin | 0.028 | 0.890 | 0.954 |
| *Syntrophomonas* | Lentialexin | -0.101 | 0.993 | 0.998 |
| *Herbaspirillum* | Lentialexin | -0.209 | 0.406 | 0.704 |
| *Gastranaerophilales* | Lentialexin | 0.218 | 0.890 | 0.952 |
| *Prevotella_1* | 2-Phenylbutyric acid | 0.572 | 0.001 | 0.040 |
| *Mollicutes_RF39* | 2-Phenylbutyric acid | 0.123 | 0.350 | 0.742 |
| *Lachnospiraceae* | 2-Phenylbutyric acid | 0.578 | 0.002 | 0.042 |
| *Bifidobacterium* | 2-Phenylbutyric acid | 0.110 | 0.404 | 0.771 |
| *Izimaplasmatales* | 2-Phenylbutyric acid | 0.545 | 0.001 | 0.040 |
| *Bacteroidales* | 2-Phenylbutyric acid | 0.189 | 0.148 | 0.663 |
| *Ruminiclostridium_9* | 2-Phenylbutyric acid | -0.033 | 0.805 | 0.945 |
| *Ornithinibacillus* | 2-Phenylbutyric acid | -0.082 | 0.534 | 0.876 |
| *CAG-352* | 2-Phenylbutyric acid | 0.175 | 0.181 | 0.676 |

Abbreviations: R correlation coefficients, FDR false discovery rate.

Table S16 Continued

| Items | Items | R | *P*-value | FDR |
| --- | --- | --- | --- | --- |
| *Bacteroidales_UCG-001* | 2-Phenylbutyric acid | -0.020 | 0.880 | 0.946 |
| *Christensenellaceae* | 2-Phenylbutyric acid | 0.016 | 0.905 | 0.958 |
| *Enterorhabdus* | 2-Phenylbutyric acid | -0.027 | 0.836 | 0.943 |
| *Lachnospiraceae_UCG-006* | 2-Phenylbutyric acid | -0.133 | 0.309 | 0.751 |
| *Neisseriaceae* | 2-Phenylbutyric acid | -0.061 | 0.643 | 0.857 |
| *Pasteurellaceae* | 2-Phenylbutyric acid | -0.271 | 0.037 | 0.525 |
| *Pseudobutyrivibrio* | 2-Phenylbutyric acid | -0.170 | 0.194 | 0.708 |
| *Lachnobacterium* | 2-Phenylbutyric acid | -0.034 | 0.794 | 0.939 |
| *Ralstonia* | 2-Phenylbutyric acid | -0.202 | 0.122 | 0.663 |
| *Moraxella* | 2-Phenylbutyric acid | -0.321 | 0.012 | 0.432 |
| *Syntrophomonas* | 2-Phenylbutyric acid | 0.069 | 0.598 | 0.875 |
| *Herbaspirillum* | 2-Phenylbutyric acid | -0.158 | 0.227 | 0.707 |
| *Gastranaerophilales* | 2-Phenylbutyric acid | -0.321 | 0.012 | 0.404 |
| *Prevotella_1* | (3R,5Z)-5-Octene-1,3-diol | 0.251 | 0.053 | 0.642 |
| *Mollicutes_RF39* | (3R,5Z)-5-Octene-1,3-diol | -0.205 | 0.117 | 0.661 |
| *Lachnospiraceae* | (3R,5Z)-5-Octene-1,3-diol | 0.124 | 0.346 | 0.740 |
| *Bifidobacterium* | (3R,5Z)-5-Octene-1,3-diol | 0.054 | 0.683 | 0.902 |
| *Izimaplasmatales* | (3R,5Z)-5-Octene-1,3-diol | 0.060 | 0.649 | 0.901 |
| *Bacteroidales* | (3R,5Z)-5-Octene-1,3-diol | 0.087 | 0.509 | 0.875 |
| *Ruminiclostridium_9* | (3R,5Z)-5-Octene-1,3-diol | 0.041 | 0.757 | 0.910 |
| *Ornithinibacillus* | (3R,5Z)-5-Octene-1,3-diol | 0.086 | 0.512 | 0.876 |
| *CAG-352* | (3R,5Z)-5-Octene-1,3-diol | 0.094 | 0.473 | 0.819 |
| *Bacteroidales_UCG-001* | (3R,5Z)-5-Octene-1,3-diol | 0.146 | 0.266 | 0.713 |
| *Christensenellaceae* | (3R,5Z)-5-Octene-1,3-diol | 0.004 | 0.973 | 0.994 |
| *Enterorhabdus* | (3R,5Z)-5-Octene-1,3-diol | -0.119 | 0.367 | 0.739 |
| *Lachnospiraceae_UCG-006* | (3R,5Z)-5-Octene-1,3-diol | -0.330 | 0.010 | 0.066 |
| *Neisseriaceae* | (3R,5Z)-5-Octene-1,3-diol | -0.115 | 0.382 | 0.764 |
| *Pasteurellaceae* | (3R,5Z)-5-Octene-1,3-diol | -0.290 | 0.025 | 0.444 |
| *Pseudobutyrivibrio* | (3R,5Z)-5-Octene-1,3-diol | -0.289 | 0.025 | 0.446 |
| *Lachnobacterium* | (3R,5Z)-5-Octene-1,3-diol | -0.189 | 0.148 | 0.664 |
| *Ralstonia* | (3R,5Z)-5-Octene-1,3-diol | -0.198 | 0.129 | 0.662 |
| *Moraxella* | (3R,5Z)-5-Octene-1,3-diol | -0.353 | 0.006 | 0.659 |
| *Syntrophomonas* | (3R,5Z)-5-Octene-1,3-diol | -0.225 | 0.084 | 0.066 |
| *Herbaspirillum* | (3R,5Z)-5-Octene-1,3-diol | -0.346 | 0.007 | 0.647 |
| *Gastranaerophilales* | (3R,5Z)-5-Octene-1,3-diol | -0.353 | 0.006 | 0.642 |
| *Prevotella_1* | N-Acetylcadaverine | 0.276 | 0.033 | 0.565 |
| *Mollicutes_RF39* | N-Acetylcadaverine | -0.274 | 0.034 | 0.561 |
| *Lachnospiraceae* | N-Acetylcadaverine | 0.089 | 0.501 | 0.875 |
| *Bifidobacterium* | N-Acetylcadaverine | 0.075 | 0.571 | 0.875 |
| *Izimaplasmatales* | N-Acetylcadaverine | 0.125 | 0.340 | 0.762 |
| *Bacteroidales* | N-Acetylcadaverine | 0.094 | 0.474 | 0.798 |

Abbreviations: R correlation coefficients, FDR false discovery rate.

Table S16 Continued

| Items | Items | R | *P*-value | FDR |
| --- | --- | --- | --- | --- |
| *Ruminiclostridium_9* | N-Acetylcadaverine | 0.166 | 0.206 | 0.736 |
| *Ornithinibacillus* | N-Acetylcadaverine | 0.076 | 0.562 | 0.874 |
| *CAG-352* | N-Acetylcadaverine | 0.103 | 0.436 | 0.804 |
| *Bacteroidales_UCG-001* | N-Acetylcadaverine | 0.137 | 0.298 | 0.733 |
| *Christensenellaceae* | N-Acetylcadaverine | -0.016 | 0.901 | 0.957 |
| *Enterorhabdus* | N-Acetylcadaverine | -0.092 | 0.483 | 0.754 |
| *Lachnospiraceae_UCG-006* | N-Acetylcadaverine | -0.515 | 0.005 | 0.082 |
| *Neisseriaceae* | N-Acetylcadaverine | -0.098 | 0.458 | 0.612 |
| *Pasteurellaceae* | N-Acetylcadaverine | -0.556 | 0.001 | 0.036 |
| *Pseudobutyrivibrio* | N-Acetylcadaverine | -0.588 | 7.63×10-5 | 0.010 |
| *Lachnobacterium* | N-Acetylcadaverine | -0.335 | 0.009 | 0.201 |
| *Ralstonia* | N-Acetylcadaverine | -0.296 | 0.022 | 0.461 |
| *Moraxella* | N-Acetylcadaverine | -0.595 | 3.90×10-5 | 0.025 |
| *Syntrophomonas* | N-Acetylcadaverine | -0.244 | 0.060 | 0.661 |
| *Herbaspirillum* | N-Acetylcadaverine | -0.333 | 0.009 | 0.410 |
| *Gastranaerophilales* | N-Acetylcadaverine | -0.585 | 3.90×10-5 | 0.025 |

Abbreviations: R correlation coefficients, FDR false discovery rate.
