# Supplementary material for: Rumen microbiome structure and metabolites activity in dairy cows with clinical and subclinical mastitis
Source: J Anim Sci Biotechnol. 2021 Feb 8;12:36. doi: 10.1186/s40104-020-00543-1 (PMC7869221; doi:10.1186/s40104-020-00543-1)
Supplement: Supplementary file 2 — Additional file 2 Fig. S1 Pan-Species curve of OTU number in the cows with different udder health status. Pan species was the sum of all the species in a sample, which was used to observe the increase in the total number of species as the number of samples increases. H, healthy; SM, subclinical mastitis; CM, clinical mastitis. Fig. S2 Rarefaction curve of OUT number in the cows with different udder health status. H, healthy; SM, subclinical mastiti; CM, clinical mastitis. Fig. S3 Rumen microbial community composition analysis. a At phylum level. b At genus level. H, healthy; SM, subclinical mastitis; CM, clinical mastitis. Fig.S4 Hierarchical cluster analysis (HCA) of rumen bacteria at genus level. Each row in the figure represents a sample, each column represents a genus, and the color indicates the relative abundance of bacteria measured in the group. Red indicates the high relative abundance, and the green indicates low relative abundance. H, healthy; SM, subclinical mastitis; CM, clinical mastitis. Fig. S5 Linear discriminant analysis effect size (LEfse) analysis of multilevel species differences in ruminal microbiota. a Cladogram; b LEfSe Bar graph. H, healthy; SM, subclinical mastitis; CM, clinical mastitis; LDA, linear discriminant analysis. Fig. S6 The total ion chromatograms (TIC) plot of quality control (QC) samples in a positive ion mode and b negative ion mode. Fig. S7 Orthogonal partial least squares discriminant analysis (OPLS-DA) (a, c, e) and response permutation testing (RPT) (b, d, f) of rumen metabolites between H, SM and CM groups in positive ion mode. H, healthy; SM, subclinical mastitis; CM, clinical mastitis. R2X and R2Y represent the interpretation rate of the built model to the X and Y matrix, R2X (cum) and R2Y (cum) represent the cumulative interpretation rate; Q2 indicates the predictive power of the model. Fig. S8 Orthogonal partial least squares discriminant analysis (OPLS-DA) (a, c, e) and response permutation testing (RPT) (b, d, f) pl [file 40104_2020_543_MOESM2_ESM.docx]

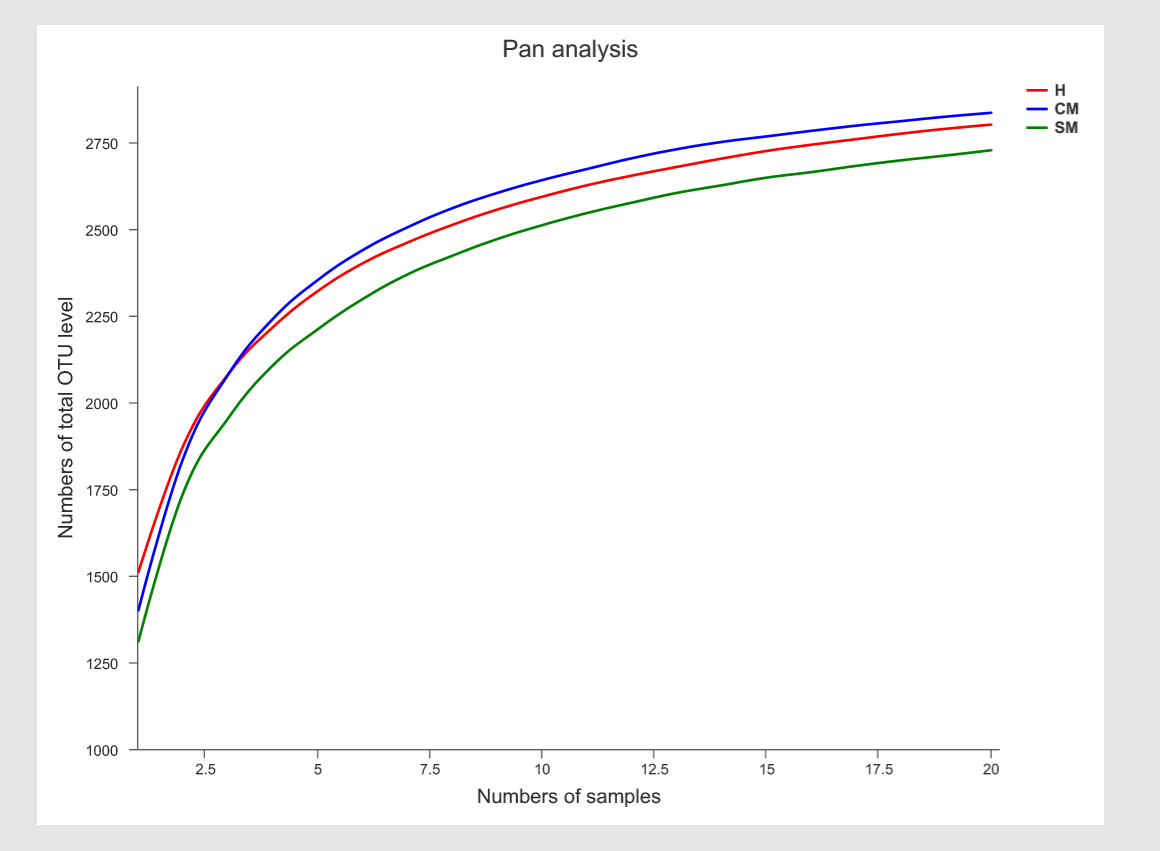
**Fig. S1** Pan-Species curve of OTU number in the cows with different udder health status. Pan species was the sum of all the species in a sample, which was used to observe the increase in the total number of species as the number of samples increases. H, healthy; SM, subclinical mastitis; CM, clinical mastitis.

**
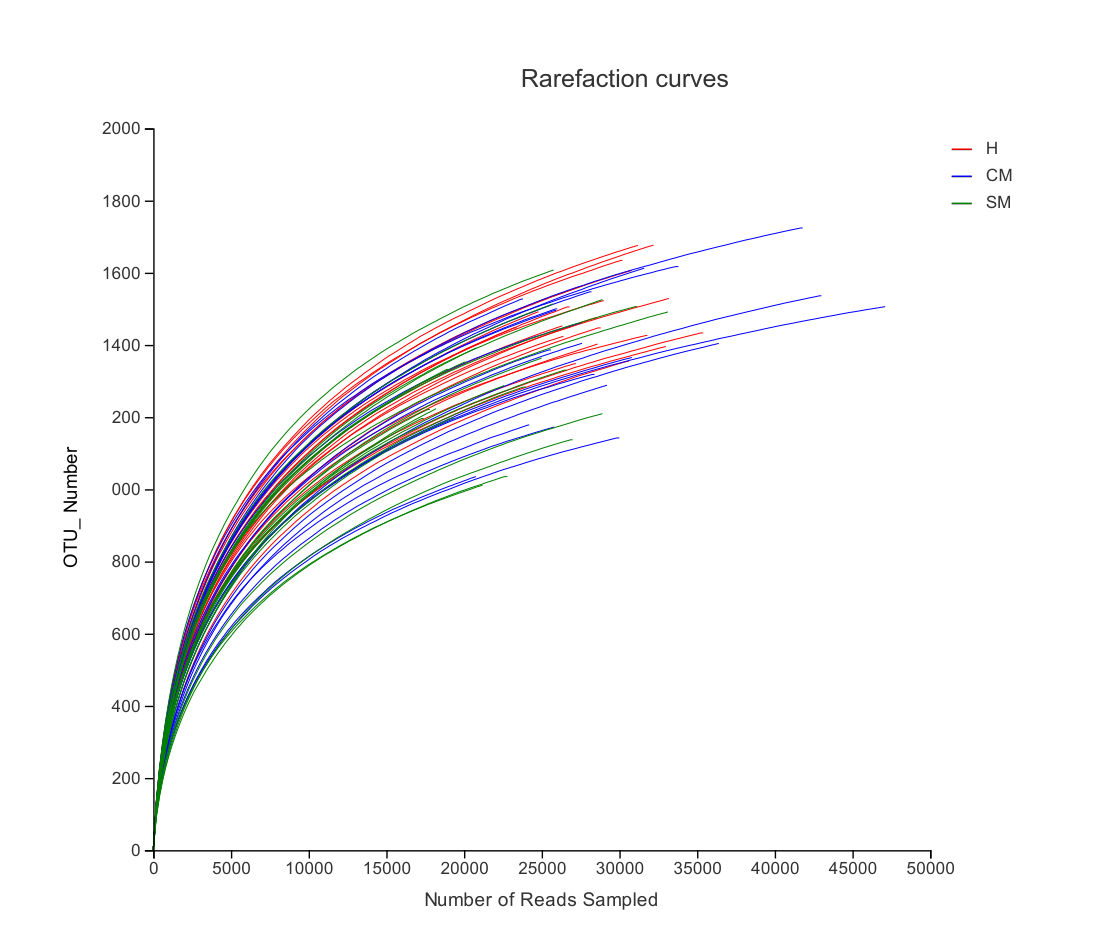
**

**Fig. S2** Rarefaction curve of OUT number in the cows with different udder health status. H, healthy; SM, subclinical mastiti; CM, clinical mastitis.


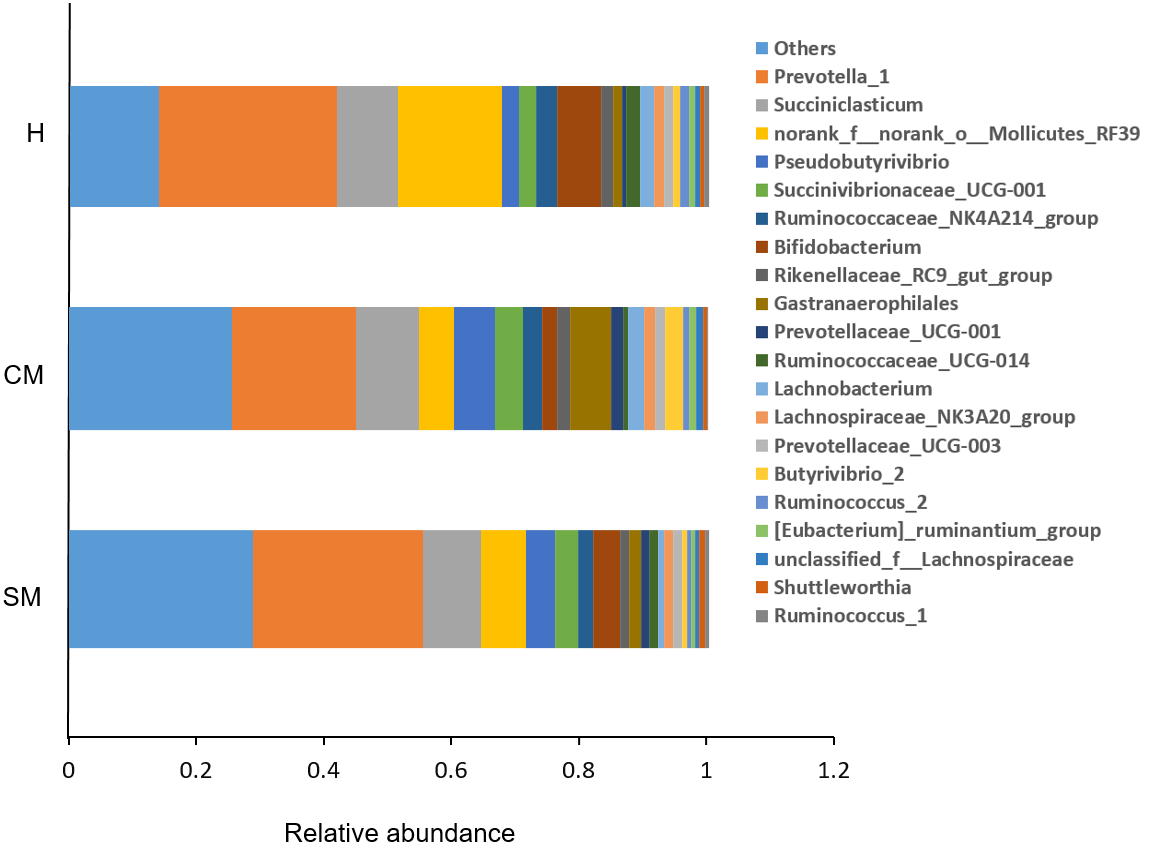

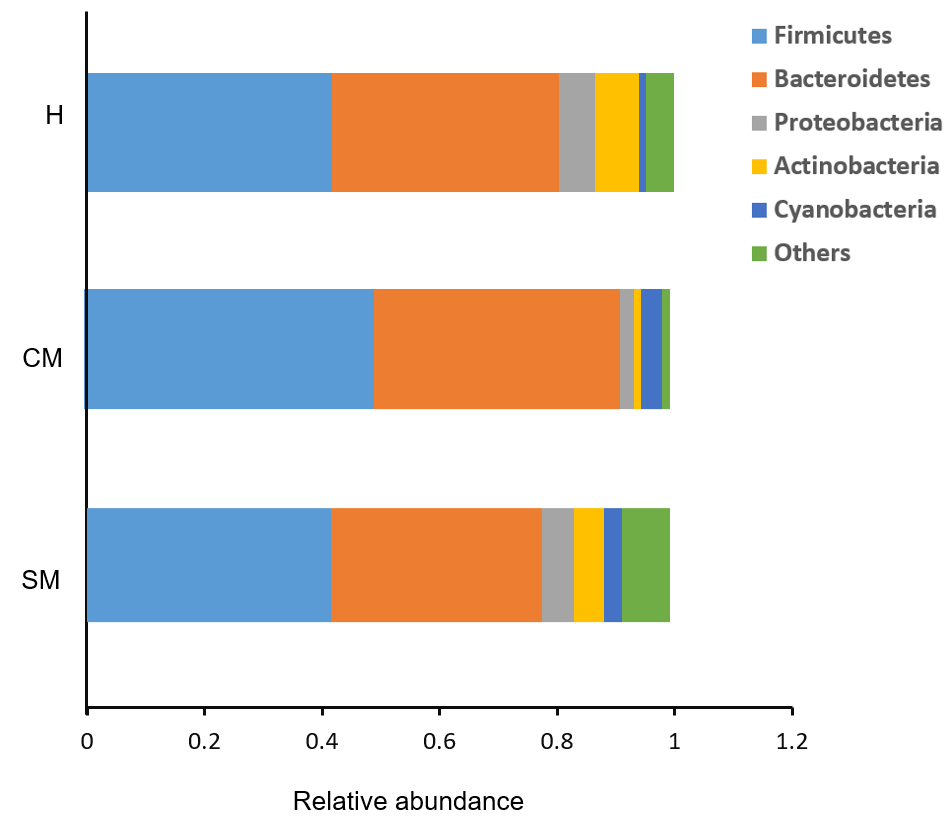


**A**

**B**

**Fig. S3** Rumen microbial community composition analysis. **a** At phylum level. **b** At genus level. H, healthy; SM, subclinical mastitis; CM, clinical mastitis.


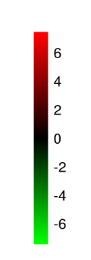

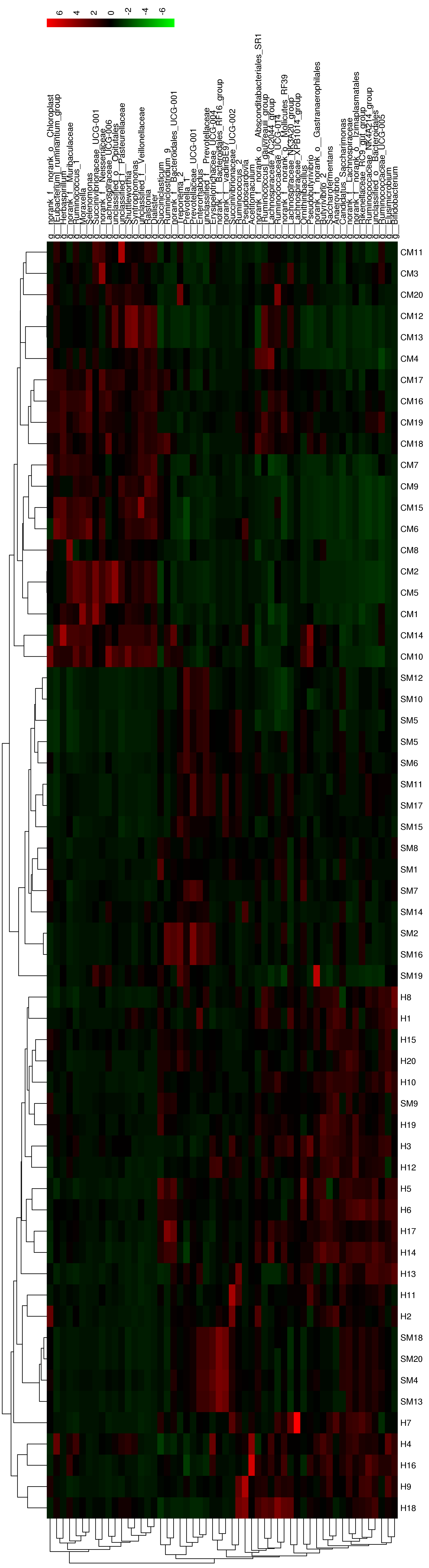


**Fig.S4** Hierarchical cluster analysis (HCA) of rumen bacteria at genus level. Each row in the figure represents a sample, each column represents a genus, and the color indicates the relative abundance of bacteria measured in the group. Red indicates the high relative abundance, and the green indicates low relative abundance. H, healthy; SM, subclinical mastitis; CM, clinical mastitis.

**A**


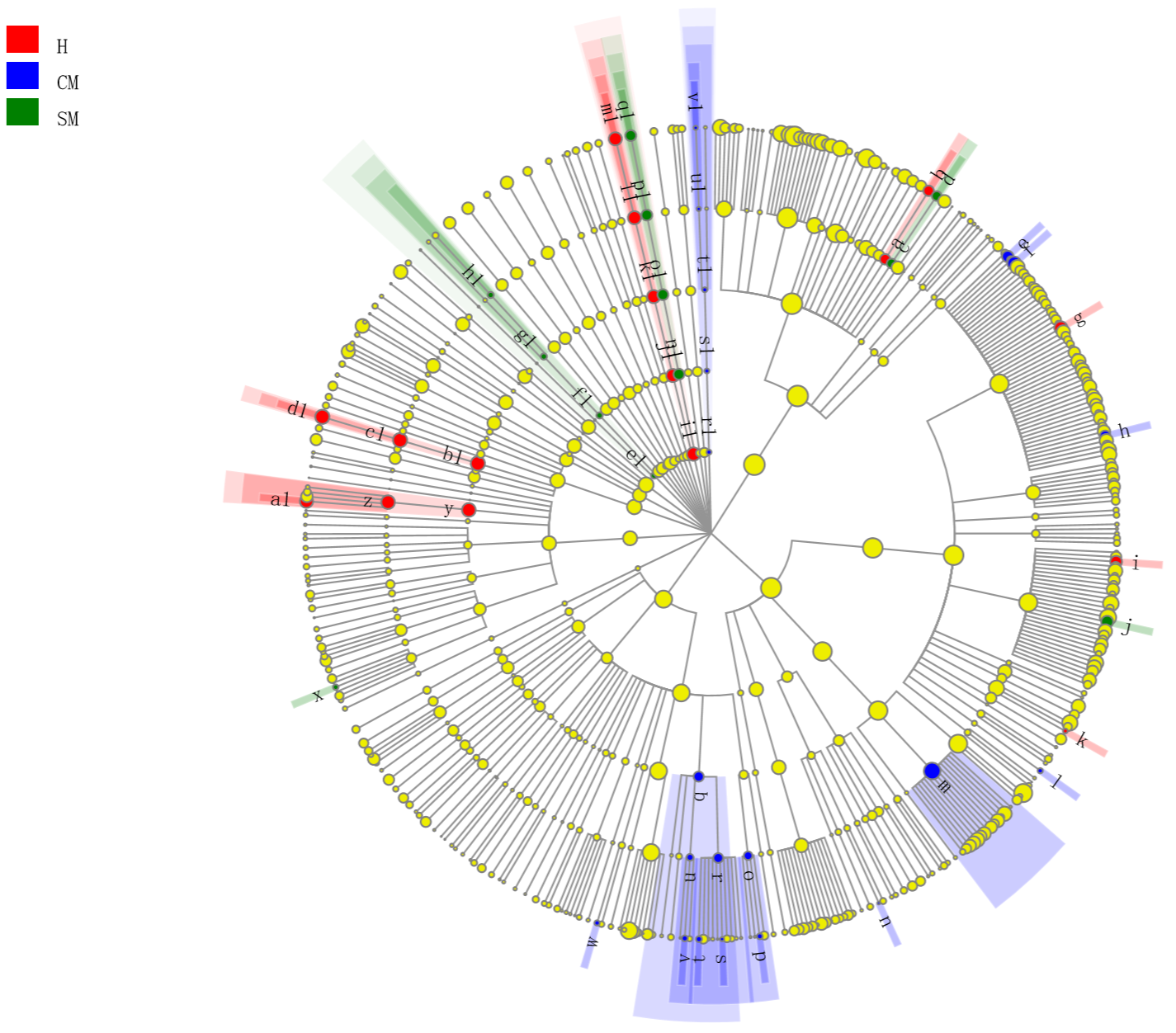

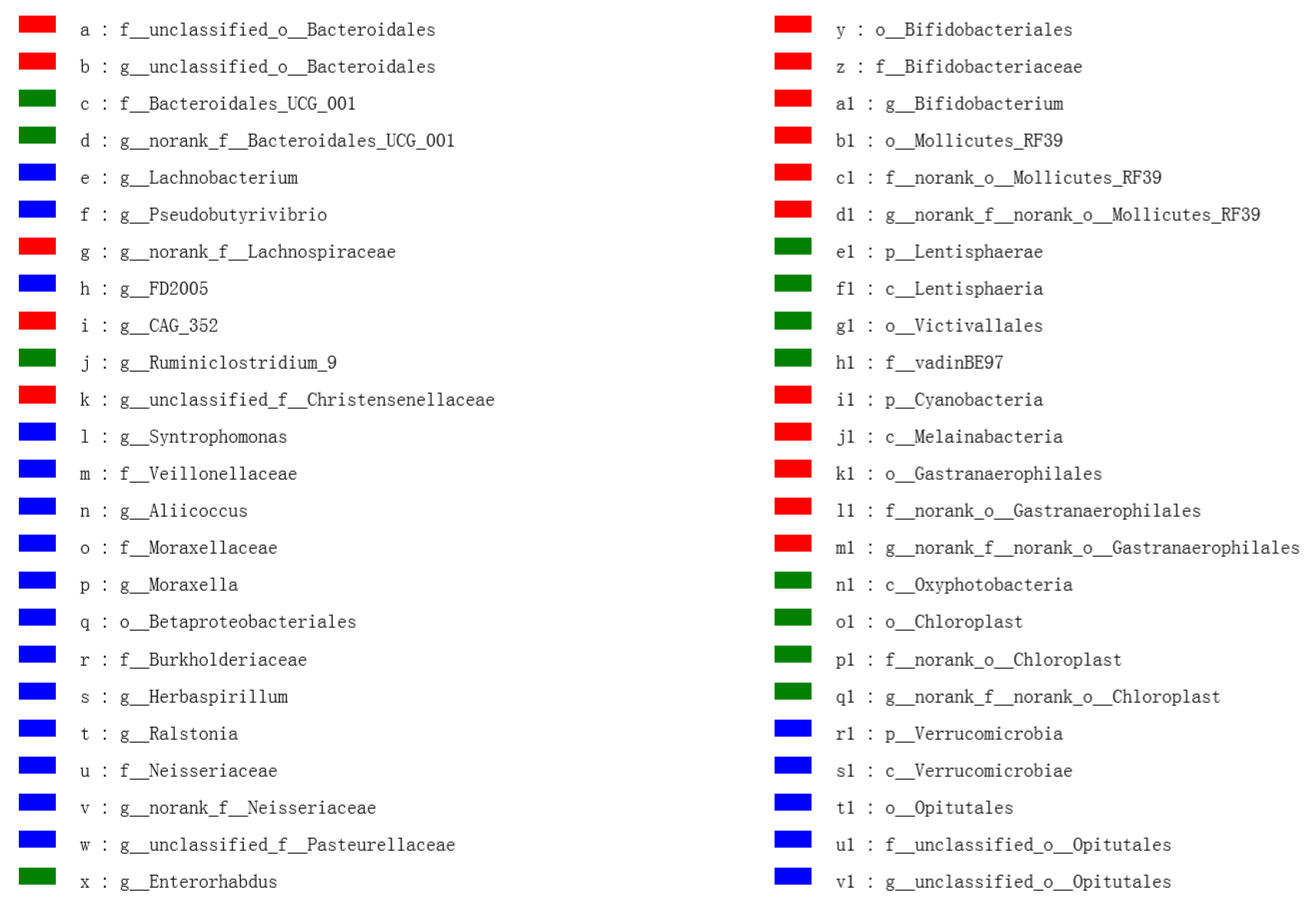

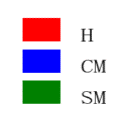


**B**


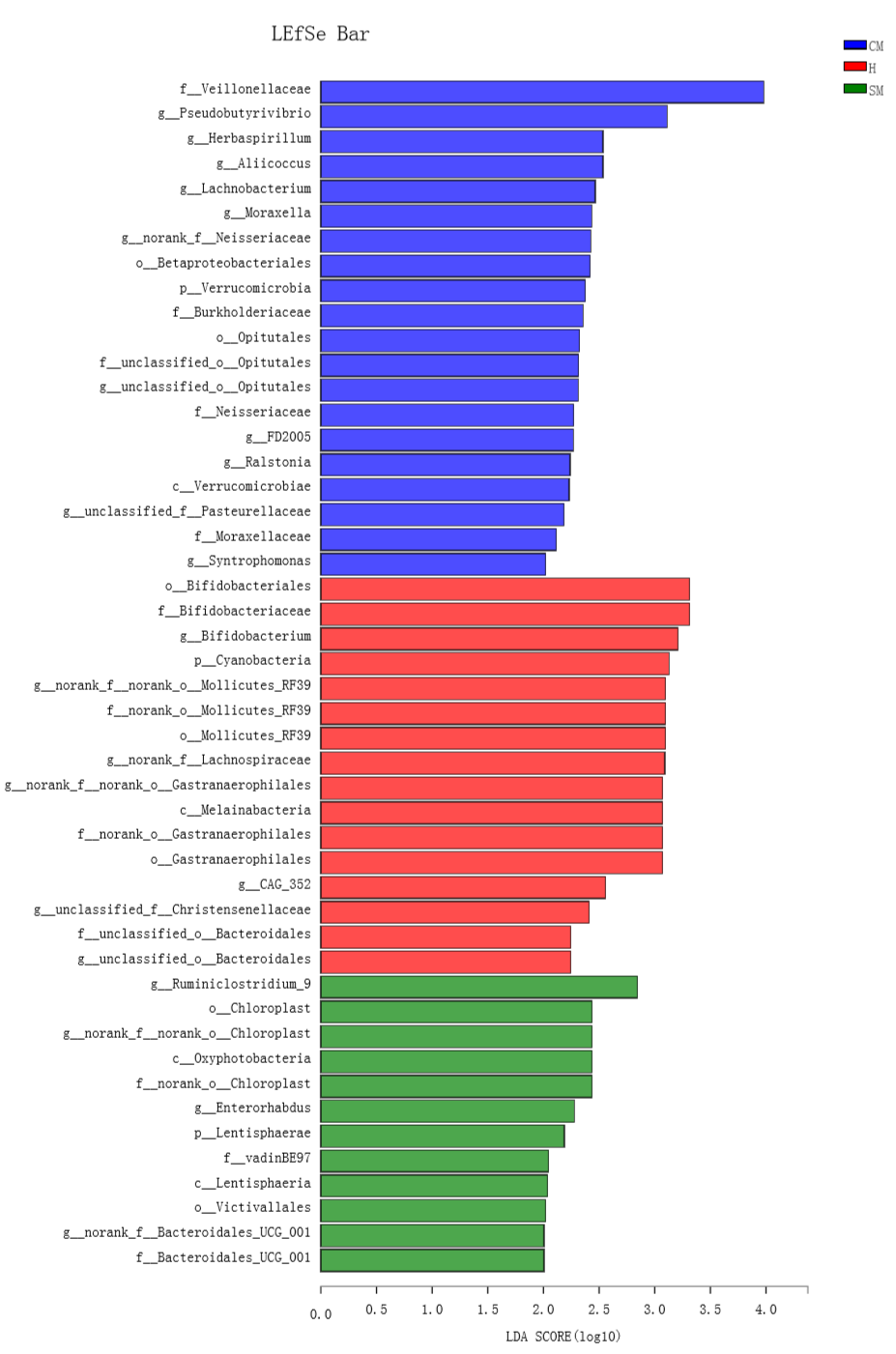


**B)**

**Fig. S5** Linear discriminant analysis effect size (LEfse) analysis of multilevel species differences in ruminal microbiota. **a** Cladogram; **b** LEfSe Bar graph. H, healthy; SM, subclinical mastitis; CM, clinical mastitis; LDA, linear discriminant analysis.


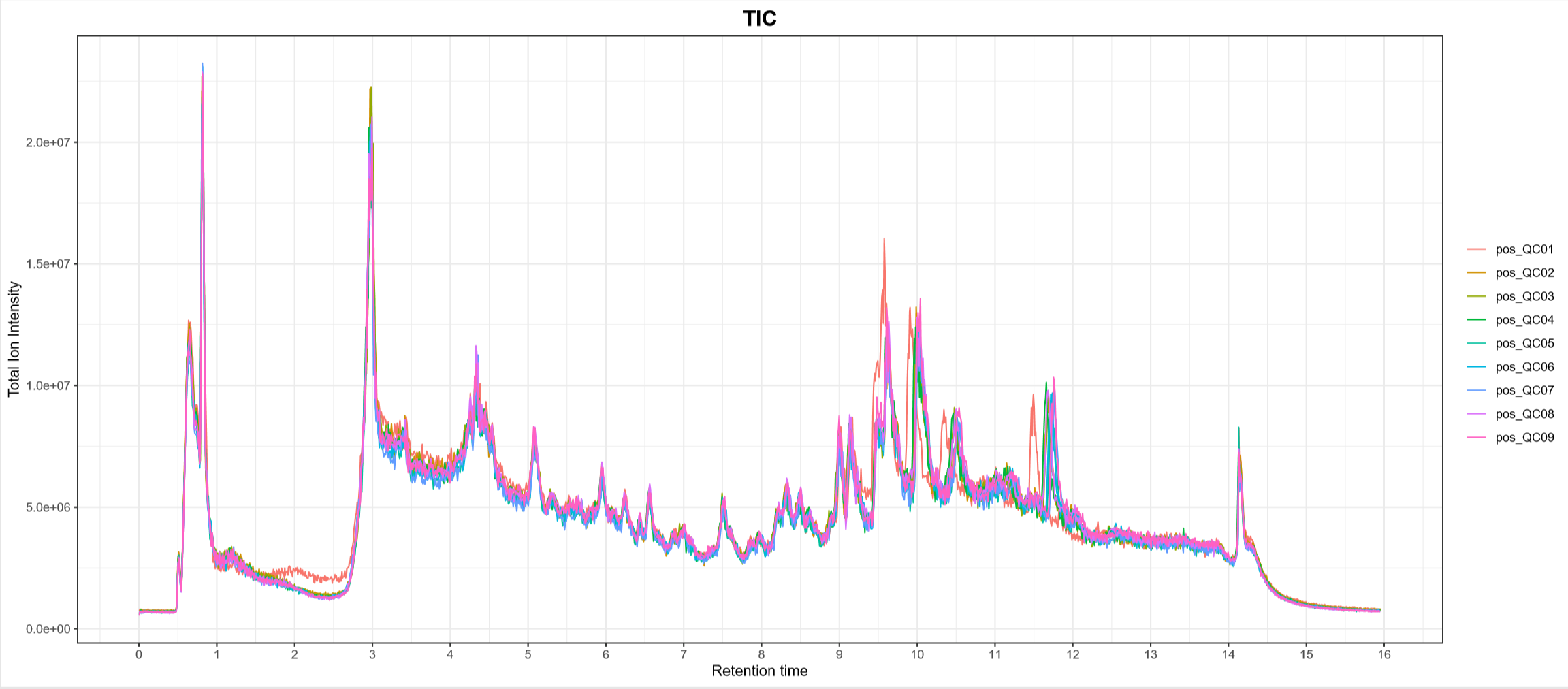
**A**

**B**

**
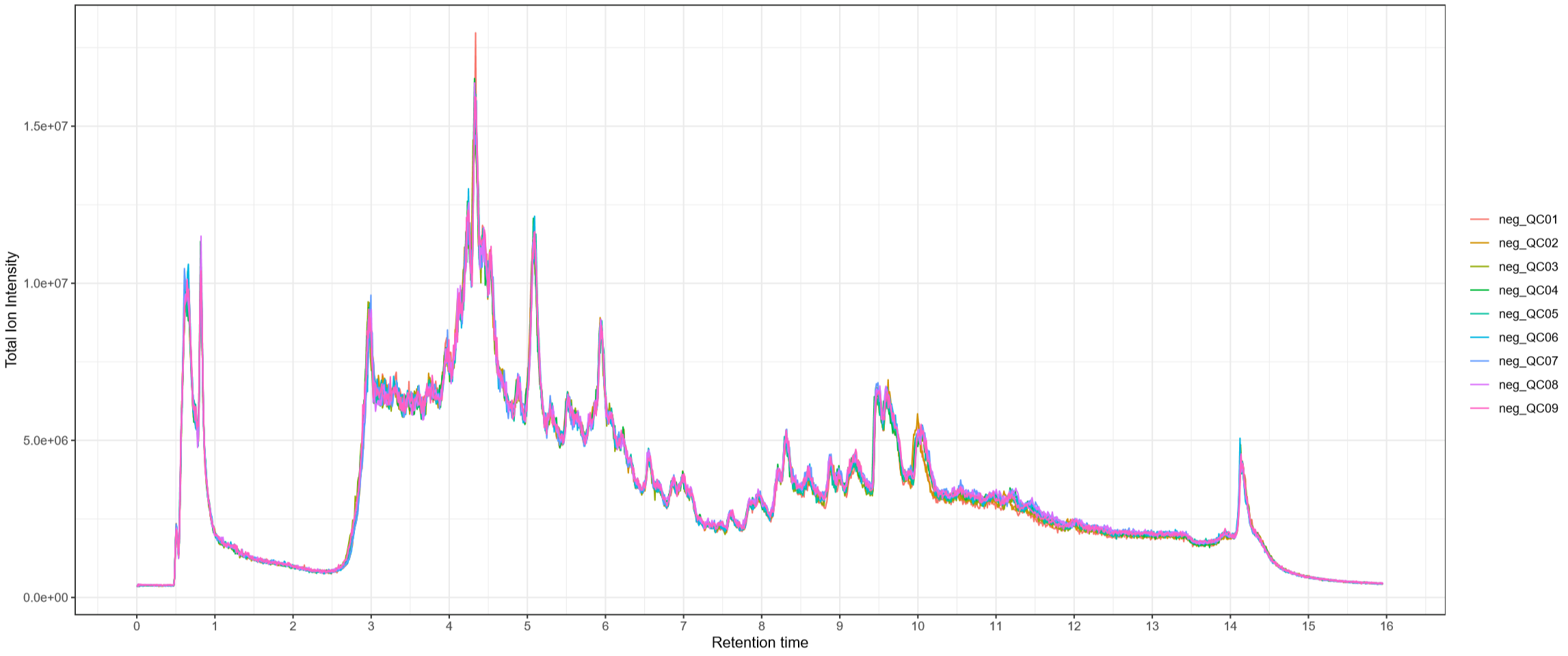
**

**Fig. S6** The total ion chromatograms (TIC) plot of quality control (QC) samples in **a** positive ion mode and **b** negative ion mode.


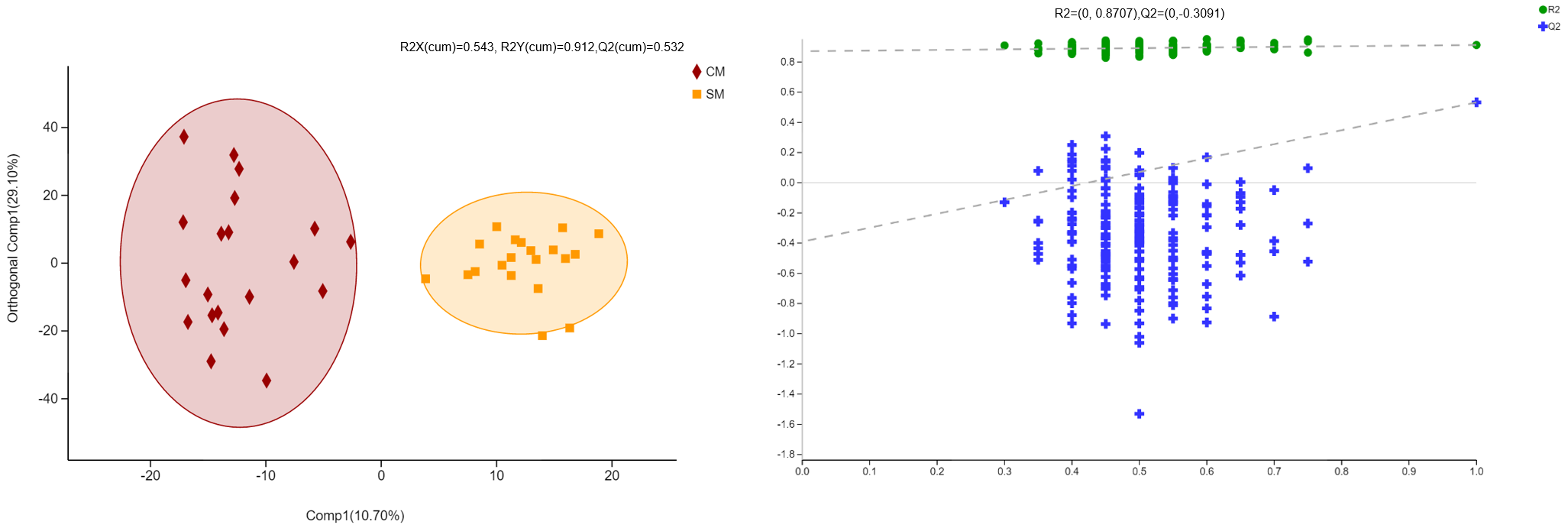

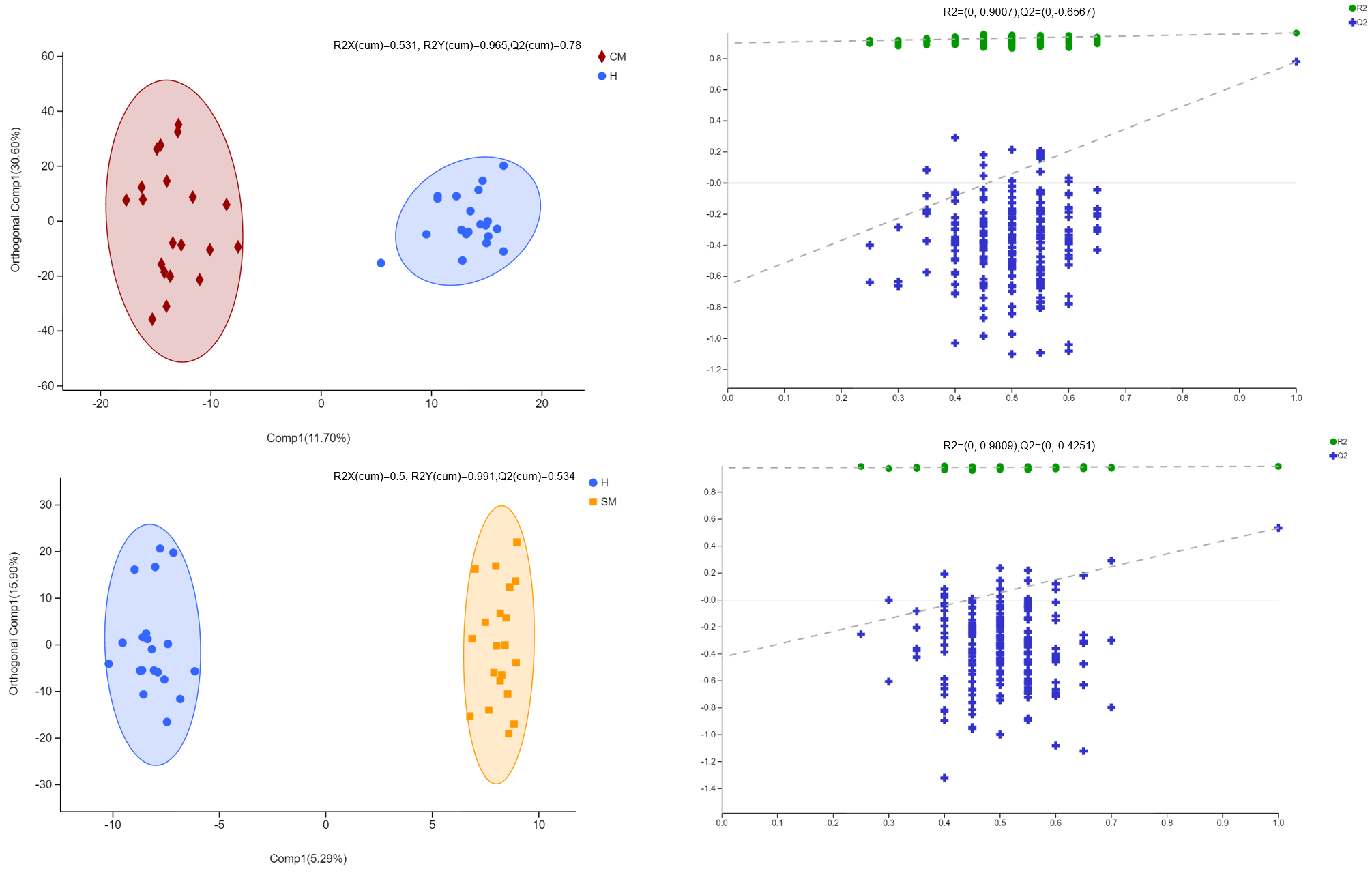


**B**

**D**

**C**

**E**

**F**

**A**

**Fig. S7** Orthogonal partial least squares discriminant analysis (OPLS-DA) (**a**, **c**, **e**) and response permutation testing (RPT) (**b**, **d**, **f**) of rumen metabolites between H, SM and CM groups in positive ion mode. H, healthy; SM, subclinical mastitis; CM, clinical mastitis. R^2^X and R^2^Y represent the interpretation rate of the built model to the X and Y matrix, R^2^X (cum) and R^2^Y (cum) represent the cumulative interpretation rate; Q^2^ indicates the predictive power of the model


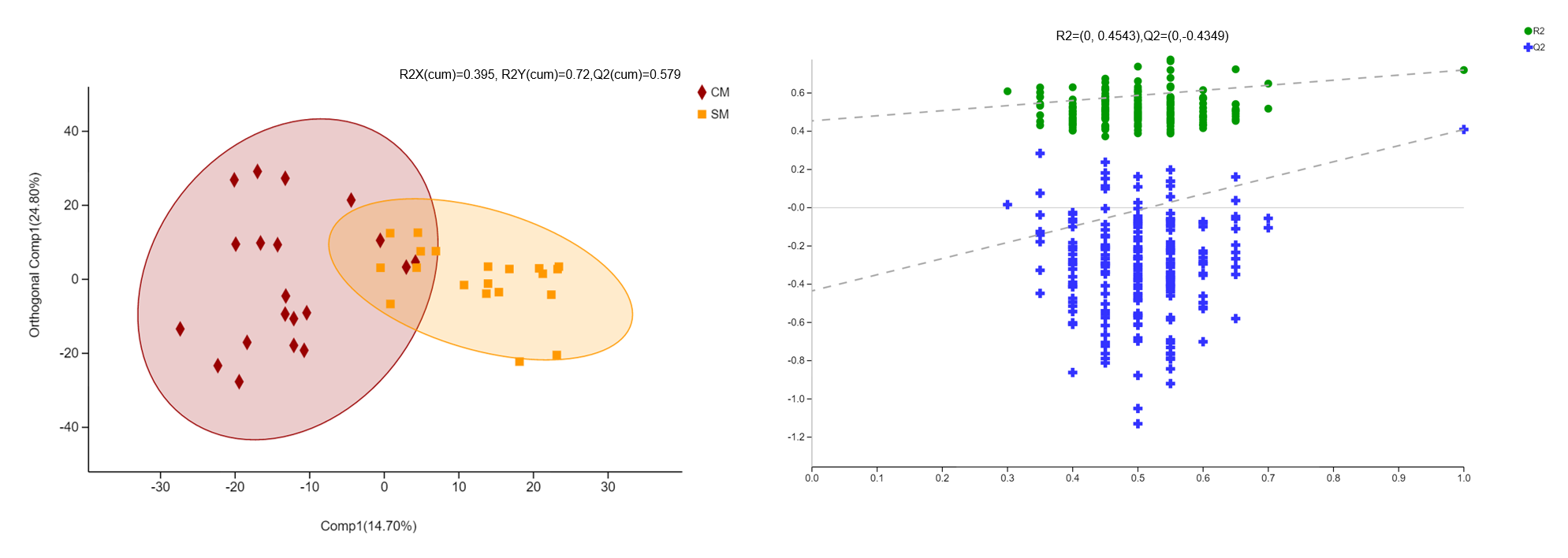

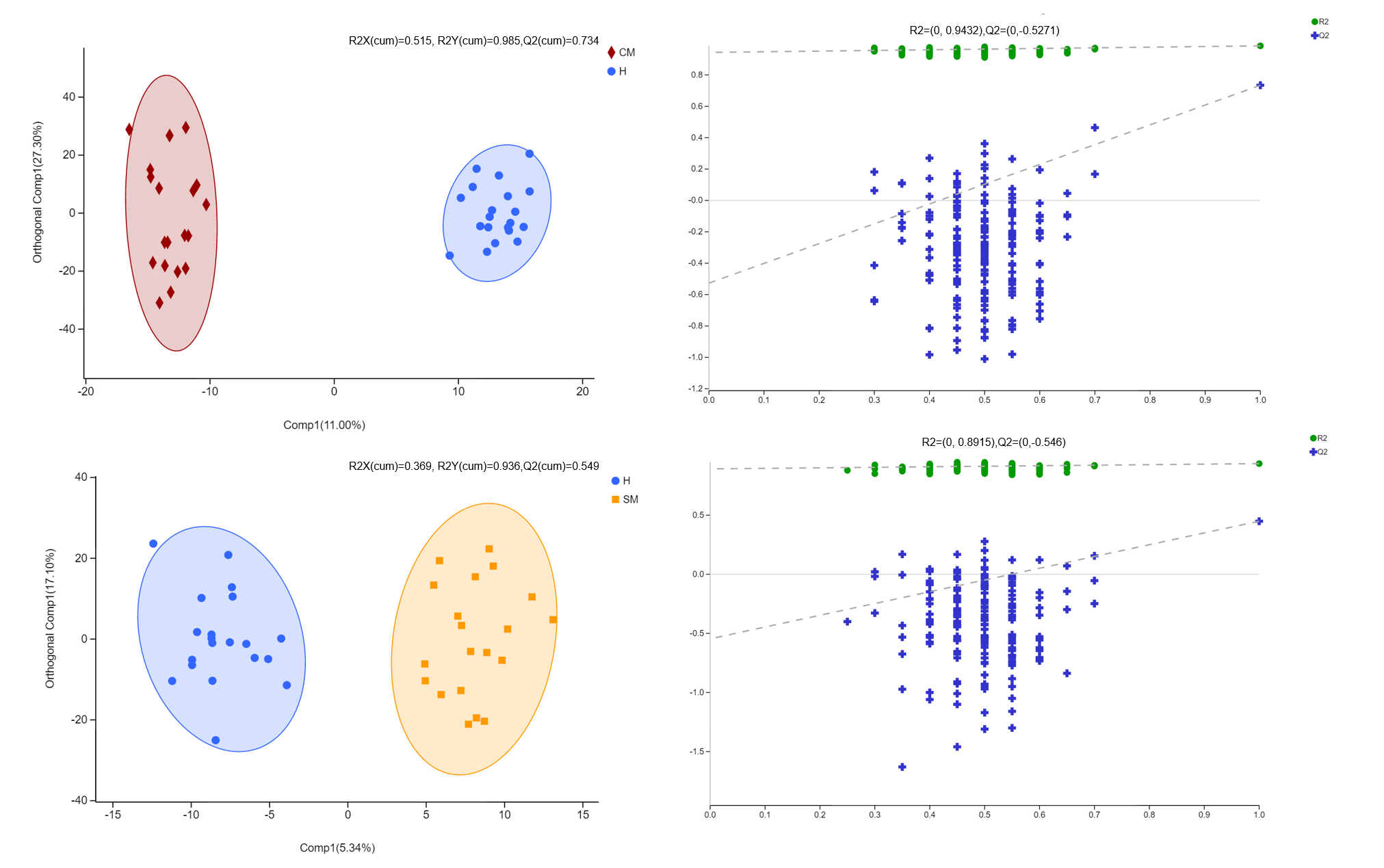
**Fig. S8** Orthogonal partial least squares discriminant analysis (OPLS-DA) (**a**, **c**, **e**) and response permutation testing (RPT) (**b**, **d**, **f**) plots of rumen metabolites between H, SM and CM groups in negative ion mode. H, healthy; SM, subclinical mastitis; CM, clinical mastitis. R^2^X and R^2^Y represent the interpretation rate of the built model to the X and Y matrix, R^2^X (cum) and R^2^Y (cum) represent the cumulative interpretation rate; Q^2^ indicates the predictive power of the model.

**F**

**E**

**D**

**C**

**B**

**A**


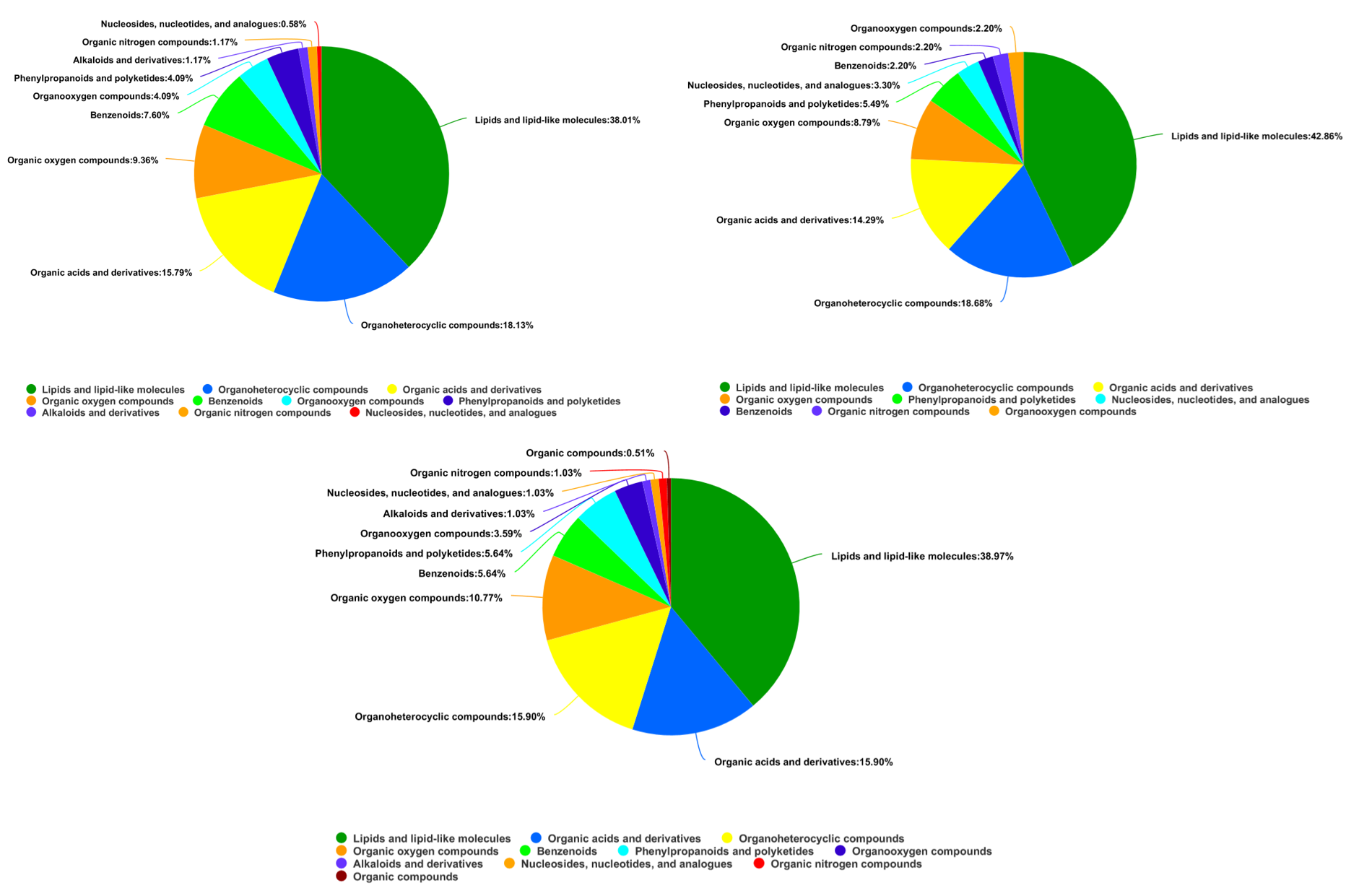


**C**

**B**

**A**

**Fig. S9** HMDB compound classification (Superclass level) of significantly differential metabolites between **a** CM and H groups, **b** SM and H groups and **c** CM and SM groups. HMDB, Human Metabolome Database; H, healthy; SM, subclinical mastitis; CM, clinical mastitis.
